# Degradation of lipid droplets by chimeric autophagy-tethering compounds

**DOI:** 10.1038/s41422-021-00532-7

**Published:** 2021-07-08

**Authors:** Yuhua Fu, Ningxie Chen, Ziying Wang, Shouqing Luo, Yu Ding, Boxun Lu

**Affiliations:** 1 State Key Laboratory of Medical Neurobiology and MOE Frontiers Center for Brain Science, Huashan Hospital, School of Life Sciences, Fudan University, Shanghai, China; 2 Peninsula Schools of Medicine and Dentistry, Institute of Translational and Stratified Medicine, University of Plymouth, Plymouth, UK

**Keywords:** Macroautophagy, Molecular biology

## Abstract

Degrading pathogenic proteins by degrader technologies such as PROTACs (proteolysis-targeting chimeras) provides promising therapeutic strategies, but selective degradation of non-protein pathogenic biomolecules has been challenging. Here, we demonstrate a novel strategy to degrade non-protein biomolecules by autophagy-tethering compounds (ATTECs), using lipid droplets (LDs) as an exemplar target. LDs are ubiquitous cellular structures storing lipids and could be degraded by autophagy. We hypothesized that compounds interacting with both the LDs and the key autophagosome protein LC3 may enhance autophagic degradation of LDs. We designed and synthesized such compounds by connecting LC3-binding molecules to LD-binding probes via a linker. These compounds were capable of clearing LDs almost completely and rescued LD-related phenotypes in cells and in two independent mouse models with hepatic lipidosis. We further confirmed that the mechanism of action of these compounds was mediated through LC3 and autophagic degradation. Our proof-of-concept study demonstrates the capability of degrading LDs by ATTECs. Conceptually, this strategy could be applied to other protein and non-protein targets.

## Introduction

Selective degradation of pathogenic proteins by small-molecule compounds such as PROTACs (proteolysis-targeting chimeras) provides unprecedented opportunities in drug discovery.^1^ Meanwhile, there has been a lack of degrader technologies to target non-protein biomolecules, which may also have important pathological functions. Thus, a strategy to degrade these molecules (or relevant organelles) may open new avenues for drug discovery.

Lipid droplets (LDs) are ubiquitous lipid-storing cellular structures with a neutral lipid core covered by a phospholipid monolayer membrane decorated with proteins.^2^ An abnormal accumulation of LDs is involved in many diseases such as obesity, cardiovascular disease, fatty liver disease, and neurodegeneration,^3–5^ and thus enhancing LD degradation could be desired to treat some of the relevant diseases or provide better health conditions. Small LDs or portions of large LDs could be engulfed into LC3-II-positive membranes and then degraded via autophagy-lysosome pathways such as lipophagy.^6,7^ Meanwhile, lipids are not proteins and cannot be ubiquitinated, and thus most of the existing ubiquitination-dependent degrader technologies such as PROTAC are probably incapable of degrading LDs directly.

We have recently demonstrated the concept of autophagy-tethering compounds (ATTECs) as a potential strategy to harness autophagy to degrade specific disease proteins of interest (POI).^8^ Different from PROTACs, ATTECs function in a ubiquitination-independent manner. ATTECs tether the POI with autophagosomes through their direct binding to the POI and the key autophagosome-associated protein LC3. This proof-of-concept study established a high-throughput screening strategy to identify ATTECs targeting the mutant HTT protein (mHTT), which is the Huntington’s disease (HD)-causing protein.^9^ The study also confirmed that the ATTECs targeted mHTT to the autophagosomes for subsequent degradation without influencing the autophagy activity per se.^8^

LC3 is lipidated to form autophagosomes,^10^ and its lipidated form LC3-II is widely used as a marker for autophagosomes.^11^ Since LDs are also subject to engulfment by LC3-II-positive membranes and subsequent autophagic degradation,^6^ the ATTECs concept could be applied to LDs as well. We hypothesized that ATTECs interacting with both LC3 and LDs may enhance the autophagic degradation of LDs. We designed such compounds (LD·ATTECs) by linking the LC3-binding molecules with LD detection probes, which are known specific LD-binding molecules.^12^ We then investigated the effects of these compounds on LDs and their mechanisms of action. The LD degradation achieved by LD·ATTECs may demonstrate the concept of using ATTECs for selective degradation of non-protein biomolecules, opening new avenues for drug discovery.

## Results

### Compound design and synthesis

**Fig. 1:**
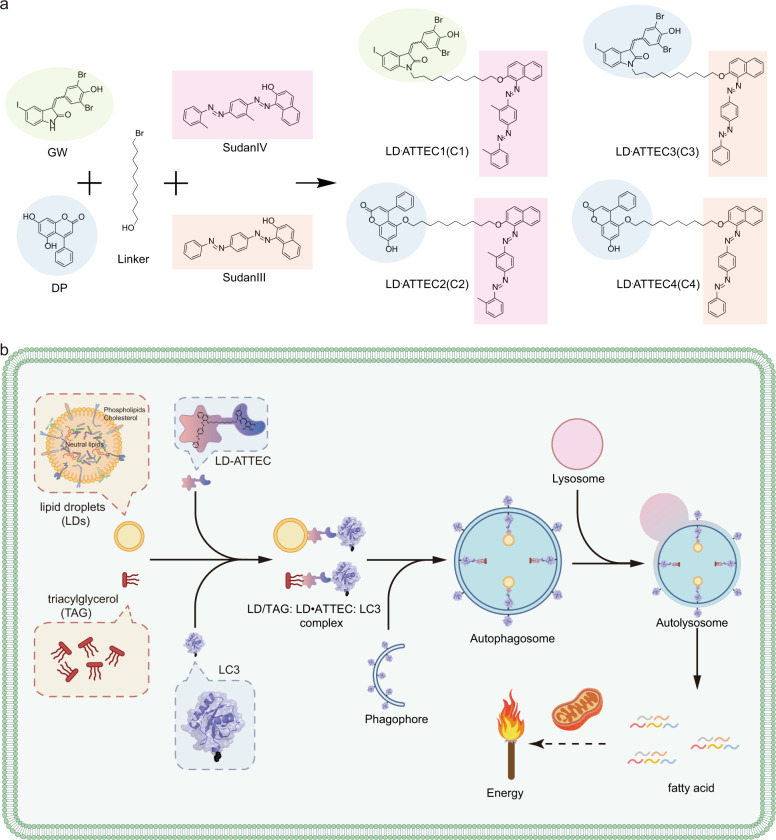
Compound structures and designing/working principle. **a** The simplified synthesis diagram of LD·ATTECs (C1–C4) utilized in this study. **b** A schematic illustration of the mechanism of action of LD·ATTEC-mediated lipid degradation. LD·ATTECs bind to LDs or neutral lipids (using TAG as a typical example) via hydrophobic interactions and the autophagosome protein LC3 simultaneously, leading to formation of the LD/TAG–LD·ATTEC–LC3 ternary complex, engulfment of the complex into autophagosomes, and subsequent autophagic degradation of LD/neutral lipids after autophagosome–lysosome fusion, providing energy source for the cells through mitochondria.

We chose GW5074 (GW) and 5,7-Dihydroxy-4-phenylcoumarin (DP) as the LC3-binding “warheads” in the designed chimeric LD·ATTECs, because we identified and validated these two compounds as LC3B-binding compounds that do not influence the overall autophagy functions.^8^ For the LD-binding chemical moiety, we initially planned to use the oil red O (ORO), because it is a triacylglycerol (TAG)-binding compound that is widely used as a specific probe for LD detection.^12^ However, we failed to obtain commercially available ORO with sufficient purity for subsequent chemical synthesis of chimeric compounds, and thus switched to use highly similar probes, Sudan IV (SIV, 1-2-methyl-4-[(2-methylphenyl)azo]phenylazo]-2-naphthalenol) or Sudan III (SIII, 1-[4-(phenylazo)phenyl]azo]-2-naphthalenol) for the synthesis.^13–16^ We used a chemical linker to connect the LC3-binding and the LD-targeting chemical moieties (Fig. 1a), so that the assembled chimeric LD·ATTECs can interact with both LC3 and LDs simultaneously, expectedly leading to autophagic degradation of LDs or neutral lipids such as TAGs that form the core of LDs (Fig. 1b). The molecular weights of these compounds are 1039.65 Da, 772.95 Da, 1011.60 Da, and 744.89 Da, respectively (see Supplementary information, Data S1). The structures of these compounds were validated by high-resolution mass spectrometry, 1H, 13C, HSQC nuclear magnetic resonance (NMR) spectroscopy and NOESY (see Supplementary information, Data S1, S2).

### LD clearance by LD·ATTECs via autophagy

**Fig. 2:**
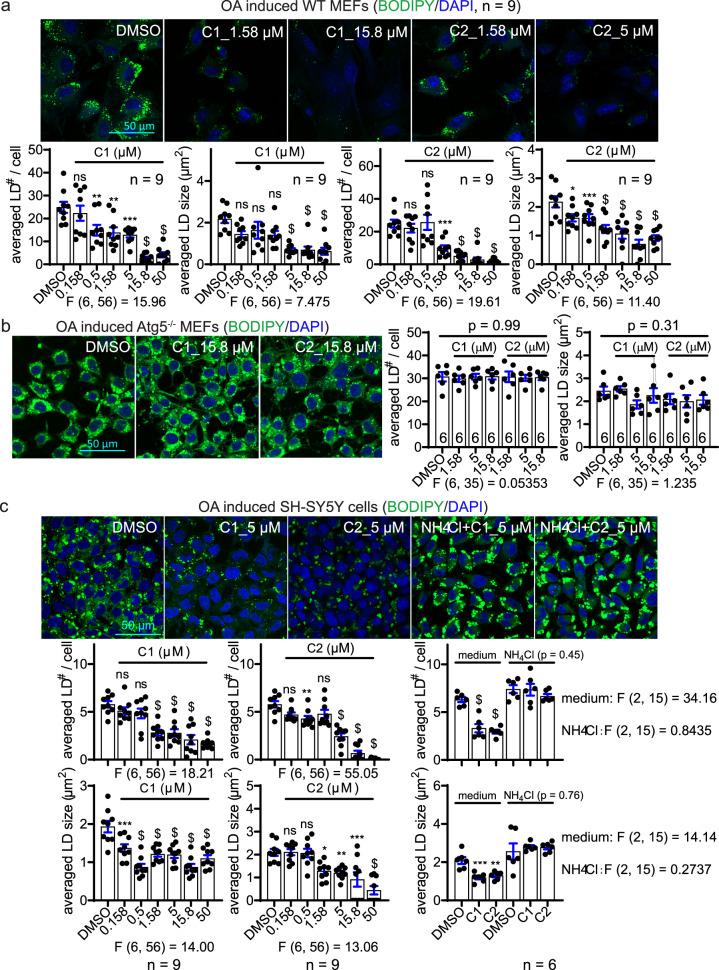
LD·ATTECs significantly reduced LDs in an autophagy-dependent manner. **a** Representative images and quantifications of OA-induced LDs in WT MEFs treated with the indicated compounds (*n* = 9, independently plated and treated wells; note that the DMSO group was the same for both the C1 and C2 plots). **b** Similar to **a**, but in Atg5^–/–^ MEFs. **c** Similar to **a**, but in SH-SY5Y cells. Note that the DMSO group was the same for both the C1 and C2 plots. For all plots, bars indicate means ± SEM. The LD number per cell and averaged LD size in each cell were quantified by ImageJ (particle analysis) in a blinded manner. The replicate number indicates the number of independently plated wells. ns, *P* > 0.05; *P < 0.05; **P < 0.01; ***P < 0.001; ^$^*P* < 0.0001 by one-way ANOVA (the F and degree of freedom values have been indicated for each plot) and Dunnett’s post hoc tests (compared to the DMSO group).

We then investigated whether the designed LD·ATTECs may lower LDs as expected. We visualized LDs by a widely-used LD-detecting probe BODIPY (BODIPY 493/503, i.e., 4,4-Difluoro-1,3,5,7,8-Pentamethyl-4-Bora-3a,4a-Diaza-s-Indacene).^17^ In wild-type (WT) mouse embryonic fibroblasts (MEFs), we utilized the standard protocol to induce LDs by extracellular oleic acid (OA) treatment^18^ (Supplementary information, Fig. S1a). The LDs were formed rapidly within 0.5 h, reaching a plateau at ~5 h after treatment, and remained stable within 24 h after induction (Supplementary information, Fig. S1b). Thus, we treated the cells with the LD·ATTEC1 (C1) or LD·ATTEC2 (C2) (Fig. 1a) 6 h after induction, when the LDs have reached the plateau already. We observed a dose-dependent reduction of both the number and size of LDs by treatment with either of the two compounds for 24 h, reaching a near complete removal of LDs at concentrations of ~5–15 μM (Fig. 2a). The observed lowering of LDs is autophagy dependent because the effects of these compounds were abolished in the cells lacking autophagy, e.g., Atg5-knockout (Atg5^–/–^) MEFs (Fig. 2b). The lack of Atg5 will prevent the LC3 lipidation and thus inhibit autophagy.^19^ A similar autophagy-dependent LD lowering phenomenon was observed in SH-SY5Y cells (Fig. 2c), a neuroblastoma cell line that has been used for LD studies previously.^20^ Interestingly, only marginal changes in LDs were observed by enhancing or inhibiting global autophagy in MEFs by starvation or NH_4_Cl treatment, respectively (Supplementary information, Fig. S2a), suggesting that selectively targeting LDs to autophagy is more efficient than enhancing global autophagy for LD degradation, at least in the OA-induced models.

We then investigated alternative mechanisms that may lead to other explanations of our observation. We had the concern that the LD-binding moiety of LD·ATTECs might have induced the lowering of BODIPY signals through a competitive binding mechanism. This scenario was excluded due to several reasons. First, the effects of these compounds were autophagy dependent (Fig. 2b, c), whereas the competition between LD·ATTECs and BODIPY should not have been. Second, we stained LDs by BODIPY in the presence of SIV (up to 50 μM) versus the DMSO control and observed no change in the BODIPY signals in the OA-induced SH-SY5Y cells (Supplementary information, Fig. S2b), suggesting that the LD-binding SIV did not compete with BODIPY for LD detection even at the highest concentration tested. This also confirms that the LD-binding moieties alone (SIV) is incapable of degrading LDs. Another alternative mechanism is that the LC3-binding chemical moieties of LD·ATTECs induced LD lowering. This was not the case because neither GW nor DP, the two LC3-binding “warheads” used for the synthesis of LD·ATTECs, induced any LD lowering (Supplementary information, Fig. S2c, d). Taken together, the connection between the LD-detecting probe and the LC3-binding moiety in the chimeric compounds is required for LD lowering, because neither of the two parts alone was capable of inducing LD lowering.

While autophagy induction alone had relatively mild effects on LDs (Supplementary information, Fig. S2a), it enhances the LD lowering by LD·ATTECs at a relatively low concentration (Supplementary information, Figs. S2e, 1 μM), including C1 and C2, as well as two similar compounds C3 and C4, which utilized SIII as the LD-binding moiety (Fig. 1). These data further supported that LD·ATTECs lowered LDs via autophagy, and may have synergistic effects with autophagy enhancers. We further confirmed the autophagy dependence for C3 and C4 by Atg5 knockout or knockdown (Supplementary information, Fig. S3a, b). We also confirmed that these compounds did not cause cytotoxicity (Supplementary information, Fig. S3c). The LD lowering by these compounds was also confirmed by imaging-independent biochemical measurements (Supplementary information, Fig. S3d).

**Fig. 3:**
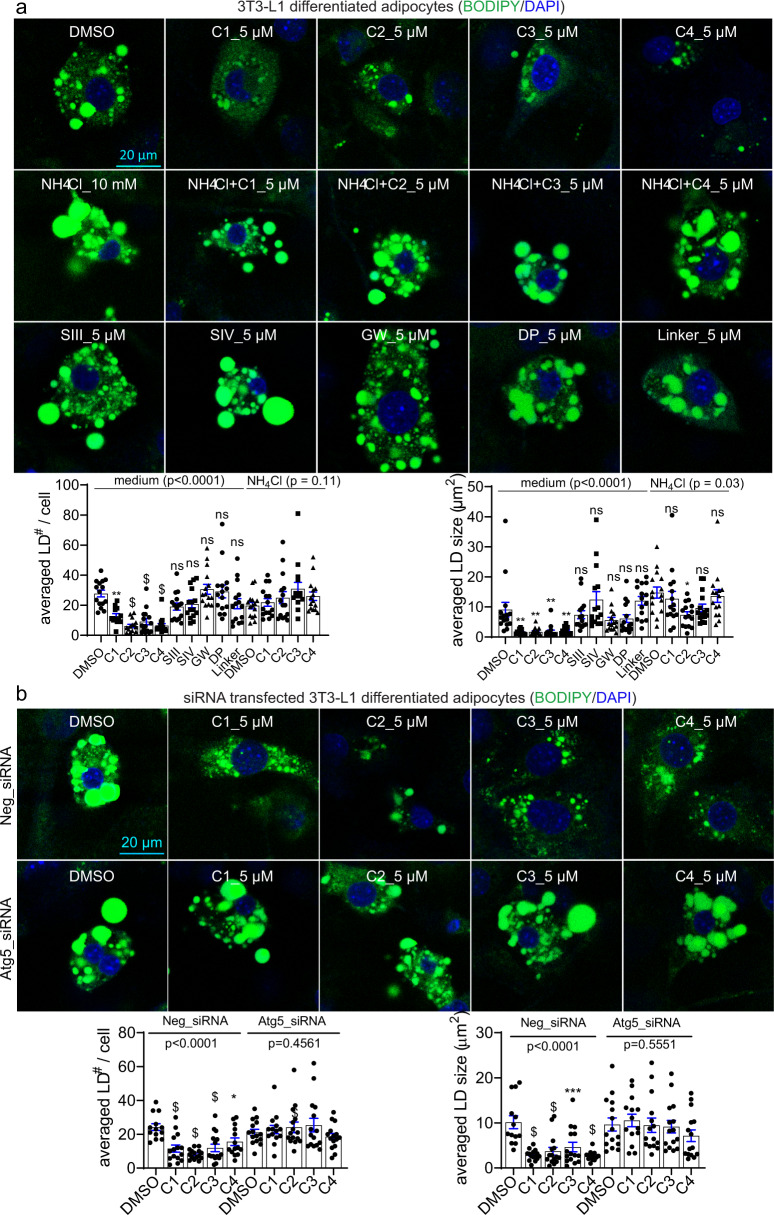
LD·ATTECs significantly reduced endogenous LDs in 3T3-L1 differentiated adipocytes. **a** Representative images and quantifications of the BODIPY493/503 staining of the endogenous LDs in the 3T3-L1 differentiated adipocytes treated with the indicated compounds (LD·ATTECs: C1, C2, C3 and C4). Bars indicate means ± SEM. The LD number per cell and averaged LD size in each field were quantified by ImageJ (particle analysis) in a blinded manner. ns, *P* > 0.05; *P < 0.05; **P < 0.01; ***P < 0.001; ^$^*P* < 0.0001 by one-way ANOVA (*P* values have been indicated) and Dunnett’s post hoc tests (compared to the DMSO group, if the ANOVA test showed significance). **b** Similar to **a**, but in cells transfected with the indicated siRNAs 48 h before treatment with compounds.

To further investigate the effects of these compounds on endogenous LDs, we cultured 3T3-L1 differentiated adipocytes, and observed significant LD-lowering effects of different LD·ATTECs (Fig. 3a). Consistent with the observation in OA-induced cells (Fig. 2c), the lowering of endogenous LDs by LD·ATTECs was also largely blocked by the autophagy inhibitor NH_4_Cl (Fig. 3a) or Atg5 knockdown (Fig. 3b), confirming its autophagy dependence. The unconjugated LC3-binding moieties or LD-binding moieties alone had no LD-lowering effects either (Fig. 3a).

### LD·ATTECs target LDs to autophagosomes without influencing global autophagy

**Fig. 4:**
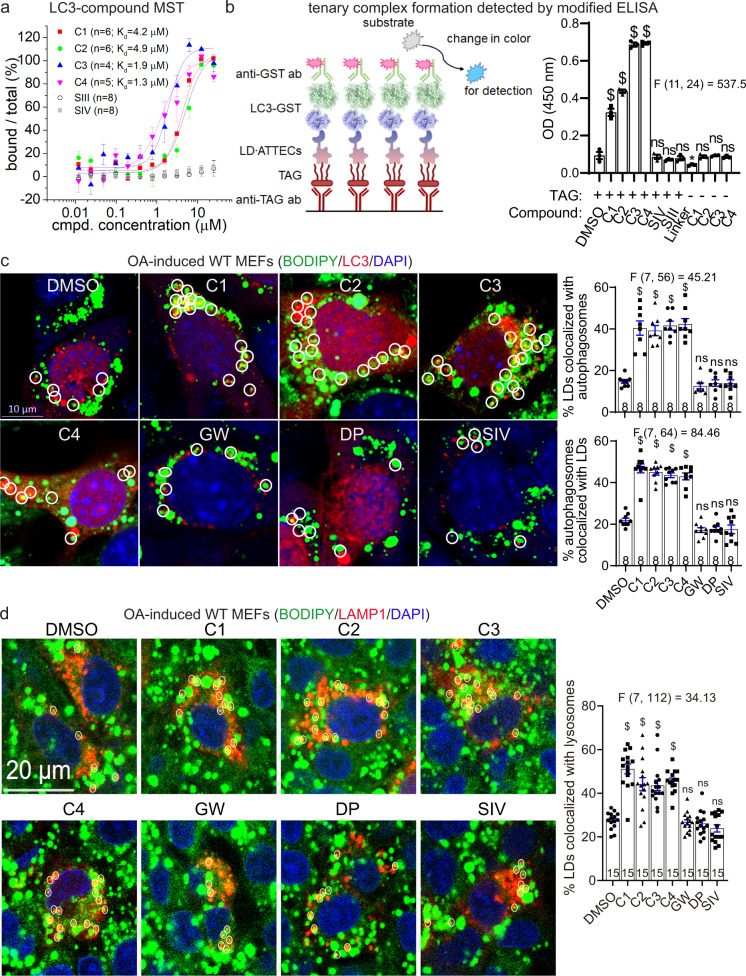
Formation of the LC3B–ATTEC–LD ternary complex and colocalization between LC3B and LD. **a** Measurements of the C1–LC3B, C2–LC3B, C3–LC3B, C4–LC3B, SIII–LC3B and SIV–LC3B binding affinity by MST. Submicromolar to micromolar *K*_d_ values were observed for the interaction between LC3B and LD·ATTECs (C1–C4), but not LD probes (SIII and SIV). **b** Left: schematic illustration of the measurements of the ternary complex formation using modified ELISA assays; Right: the blank-corrected ELISA signals of the indicated samples (*n* = 3, independent assay wells). All samples were added with recombinant purified LC3-GST for the final detection with the GST antibody, and the wells containing recombinant purified GST alone were used as the blank control. **c** Representative images and quantifications of mCherry-LC3B-transfected MEFs showing LDs (stained with BODIPY 493/503, green), autophagosomes (LC3B puncta, red) and nuclei (DAPI, blue). LD·ATTECs (C1–C4) but not the control compounds (all at 5 μM) induced significant partial colocalizations of LD and autophagosomes. The percentage of LDs that are partially colocalized with autophagosomes in each sample was analyzed by visual counting in a blinded manner. The replicate number indicates the number of fields from 2 independent batches of transfections. **d** Similar to **c**, but in LAMP1-mCherry-transfected MEFs showing colocalization between LDs and lysosomes (LAMP1). Bars indicate means ± SEM. ns, *P* > 0.05; ^$^*P* < 0.0001 by one-way ANOVA (the F and degree of freedom values have been indicated for each plot) and Dunnett’s post hoc tests (compared to the DMSO group).

ATTECs are predicted to interact with both LC3 and the target, and thus tether the target molecules to autophagosomes for subsequent degradation^8^ (see also Fig. 1b). To confirm this, we measured the affinity of LD·ATTECs to the recombinant purified LC3B protein by microscale thermophoresis (MST) and confirmed their interactions (Fig. 4a). As controls, no SIII–LC3B or SIV–LC3B interaction was detected (Fig. 4a), confirming that the LC3B-binding “warhead” is required for the LD·ATTEC–LC3 interaction. We then investigated whether LD·ATTECs enhance the interaction between LC3 and TAG, a core lipid component of LDs that may recruit SIII and SIV for LD staining.^13–16^ The hydrophobic interaction between TAG and SIII or SIV could be sufficient to tether LDs to autophagosomes in the cells and recapitulated in vitro by ELISA assay. We confirmed the formation of TAG–LD·ATTEC–LC3 ternary complex by a modified ELISA assay (Fig. 4b), suggesting that the synthesized LD·ATTECs tethered TAG to LC3B in vitro, and thus may enhance engulfment of TAG or TAG-containing LDs by autophagosomes in the cells.

To validate this predicted cellular process directly, we investigated whether LD·ATTECs could enhance the engulfment of LDs by autophagosomes through tethering LDs to LC3. We visualized autophagosomes by exogenously expressed mCherry-LC3B in the MEFs and LDs by BODIPY493/503 staining (Fig. 4c). Significant colocalization between autophagosomes and LDs was observed in these cells 2 h after adding LD·ATTECs, but not the controls (Fig. 4c), supporting the predicted mechanism of action that LD·ATTECs enhanced the recognition of LDs by autophagosomes. Consistent with the involvement of the autophagy-lysosome pathway in LD degradation, we also observed significant increase in colocalization between lysosomes (detected by LAMP1-mCherry) and LDs (Fig. 4d). Taken together, the synthesized LD·ATTECs induced the TAG–LC3B interaction and enhanced engulfment of LDs by autophagosomes.

**Fig. 5:**
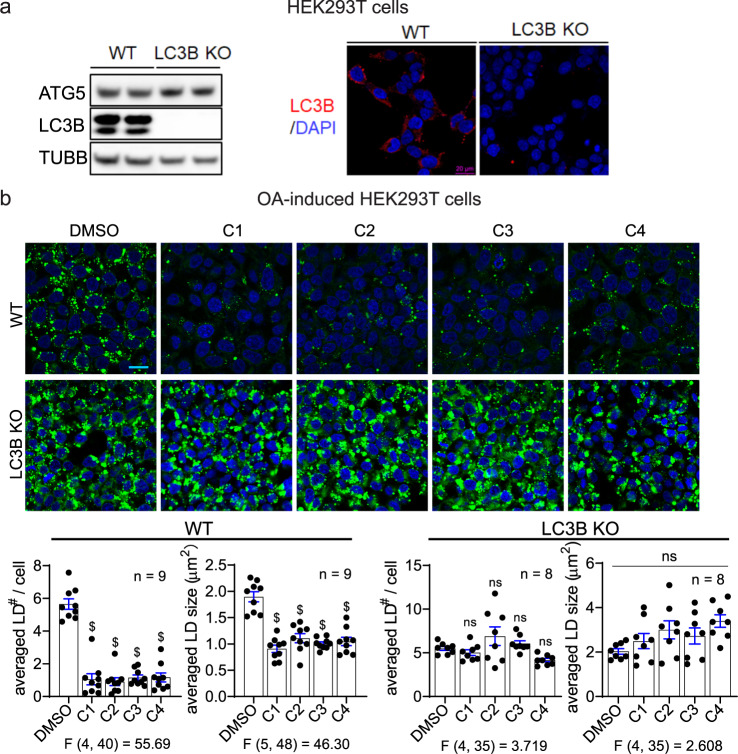
LC3B knockout abolished the effects of LD·ATTECs. **a** Representative LC3B western blot and immunofluorescence for the WT versus LC3B-knockout HEK293T cells. Note that different LC3B antibodies were used for western blot and immunofluorescence as indicated in “Material and Methods”. **b** Representative images and quantifications of the BODIPY493/503 staining of the OA-induced LDs in the WT versus LC3B-knockout HEK293T cells treated with the indicated compounds (5 μM for LD·ATTECs). Scale bar, 20 μm. The LD number per cell and averaged LD size in each field were quantified by ImageJ (particle analysis) in a blinded manner. *n* indicated the number of independently plated wells from two batches of experiments. ns, *P* > 0.05; ^$^*P* < 0.0001 by one-way ANOVA (the F and degree of freedom values have been indicated) and Dunnett’s post hoc tests (compared to the DMSO group).

To further confirm that LD·ATTECs target LDs to LC3 proteins and autophagosomes rather than directly to lysosomes for degradation, we tested the LD·ATTECs in a LC3B-knockout HEK293T cell line (Fig. 5a). The LC3B knockout largely blocked the LD clearance induced by LD·ATTECs (Fig. 5b), supporting the predicted mechanism of action (Fig. 1b). Meanwhile, since LC3B knockout may inhibit macroautophagy, we still cannot exclude the possibility that the blockade of LD clearance is due to autophagy inhibition. Mutagenesis of LD·ATTECs’ binding site of LC3B is desired to confirm the LC3 dependence, but this is technically challenging due to a lack of structural details of the LC3–LD·ATTEC interaction, which will be further investigated in future studies.

While our discovered LD·ATTECs target LDs to autophagosomes (Figs. 4 and 5), we reasoned that they probably did not influence global autophagy, because the LC3-binding chemical moiety of the designed LD·ATTECs did not influence global autophagy.^8^ We further confirmed this in MEFs treated with LD·ATTECs and observed no significant change in numbers of autophagosomes or lysosomes (Supplementary information, Fig. S4a, b). We further measured the autophagosome–lysosome fusion by the mRFP-GFP-LC3 based assay, which allows us to label autophagosomes (green and red) and autolysosomes (red), since the low lysosomal pH in autolysosomes quenches the GFP signals^21^ (Supplementary information, Fig. S4c). We observed no significant change by treatment of LD·ATTECs in this assay either. Finally, we measured the levels of autophagy markers LC3-II and SQSTM1/p62 (Supplementary information, Fig. S4d). Noticeably, the increase of LC3-II and SQSTM1/p62 levels induced by the lysosome inhibitor bafilomycin A1 (BafA) was not affected by LD·ATTECs either, confirming that autophagy flux was not altered.

**Fig. 6:**
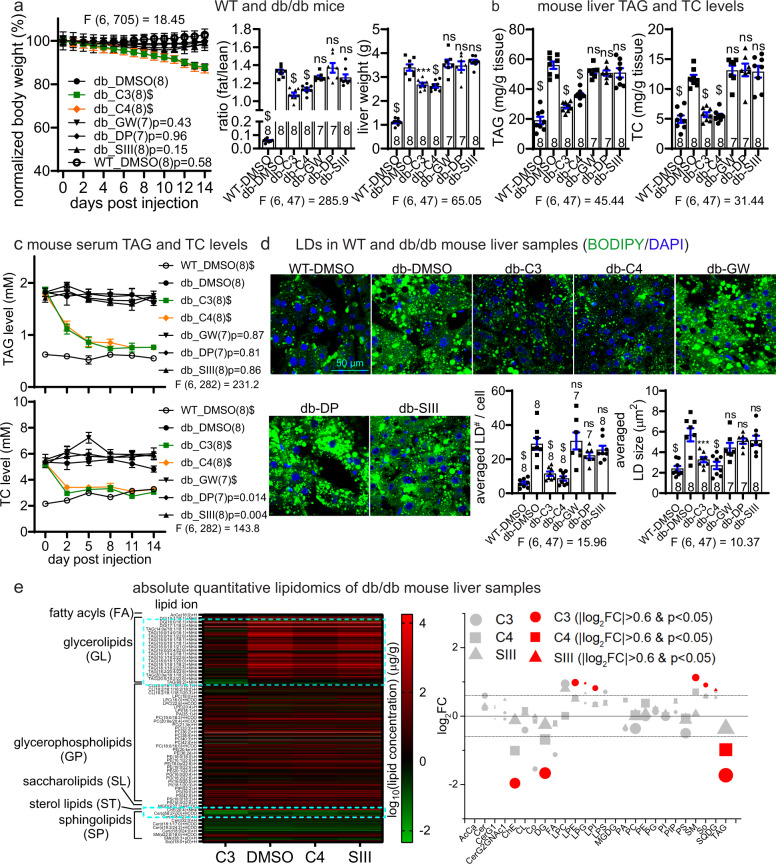
LD·ATTECs lowered body fat, neutral lipids and LDs in vivo in db/db mice. The db/db mice (db) were injected with the indicated compounds at one dosage per day for the indicated days. The WT mice fed on chow diet (WT) were used as the baseline control. **a** Measurements of body weight (measured each day and normalized to the averaged weight of day 0, left panel), fat/lean ratio (after injection for 12 days, middle panel) and liver weight (endpoint measurement after injection for 14 days, right panel) in the indicated mice injected with the indicated compounds. **b** The endpoint measurements (after injection for 14 days) of the TAG and TC levels of liver samples in the indicated mice injected with the indicated compounds. **c** Measurements of the serum TAG and TC levels in db/db mice injected with the indicated compounds. Serum was collected at several different time points after injection as indicated. **d** Representative images and quantifications of the BODIPY493/503 staining of the endogenous LDs in the liver slice samples from the mice injected with the indicated compounds for 14 days. For quantifications, images of each mouse (from at least 3 slices) were averaged. **e** Absolute quantitative lipidomics of liver in db/db mice (4 per group). Left: the averaged log_10_ expression of the lipids in the samples. The expression rather than the fold change was plotted to avoid over-representation of low-expression lipids, which typically give high fold changes. Darker red or brighter green indicates lower concentrations. The major different regions were highlighted by the dashed cyan box. The DMSO was placed in the middle for easier comparison. Right: log_2_FC compared to the DMSO group are plotted for each lipid class (X axis). A few lipid classes with extremely low abundance (total concentration < 1 μg/g) were neglected. The area of each symbol represents the abundance of the lipid class in the DMSO group. The red color indicates significant changes (|log_2_FC| > 0.6 and *P* value < 0.05 by the two-tail unpaired *t*-test). See Supplementary information, Data S3 for the detailed list of detected lipids and the table of abbreviations of lipid categories and classes. For all plots, data were plotted as means ± SEM. The replicate number indicates the number of mice. ns, *P* > 0.05; ^$^*P* < 0.0001; exact *P* values are shown if space allows. For measurements at multiple time points, two-way ANOVA (the F and degree of freedom values for the treatment factor have been indicated) and Dunnett’s post hoc tests (compared to the DMSO-treated db/db group) were performed. For endpoint measurements for multiple groups, one-way ANOVA (the F and degree of freedom values have been indicated) and Dunnett’s post hoc tests (compared to the DMSO-treated db/db group) were performed.

Finally, while LD·ATTECs lowered LDs, they did not influence the integrity of plasma or nuclear membranes (Supplementary information, Fig. S5a, b). In addition, the mitochondrial function was not impaired by the treatment of LD·ATTECs (Supplementary information, Fig. S5c), suggesting that the mitochondrial membranes were not disrupted by these compounds. This selectivity is explained by the fact that LDs contain mainly neutral lipids but not polar lipids.^22^ LD·ATTECs may selectively interact with the neutral lipids via the LD-interacting moiety. Polar lipids mainly comprise the plasma and intracellular membranes,^23^ which were intact upon LD·ATTEC treatment (Supplementary information, Fig. S5a–c). Collectively, these data suggest that LD·ATTECs did not affect the integrity and function of cellular membranes because they probably reduced mainly the neutral lipids, consistent with the lipidomics data (Fig. 6e).

### In vivo efficacy of LD·ATTECs

To investigate the effects of LD·ATTECs in vivo, we injected these compounds versus controls intraperitoneally (i.p.) into two different mouse models of metabolic disorders. One is a genetic model (db/db mice, C57BL/6J-Lepr^db^/Lepr^db^) with obesity,^24–26^ and the other is non-alcoholic steatohepatitis (NASH) mouse model generated by a choline-deficient, l-amino acid-defined, high-fat diet (CDAHFD, 60 kcal % fat) feeding for 10 weeks.^27^ We chose C3 and C4 for the in vivo injections, because they have lower molecular weights and higher affinities to LC3B (Figs. 1 and 4a). We determined 30 mg/kg as a suitable injection dose, which can reach the ~μM concentration range in the liver after i.p. injection (Supplementary information, Fig. S6a).

For both models, we injected one dose of C3 or C4 every day and monitored the animal weight. When feasible, we also collected blood serum from tail tips before injections for measurements of TAG, total cholesterol (TC), and free fatty acid (FFA) levels. After 12 days, we measured the body fat versus lean mass by NMR. Fourteen days after injection, we performed endpoint maxillofacial blood sampling for each mouse and then sacrificed the mice to isolate liver tissues for further analysis. db/db or NASH mice injected with DMSO, SIII, or LC3-binding compounds GW or DP were used as negative controls to confirm the necessity of the linkage between LC3-binding and LD-binding moieties. The WT mice at the same age fed on a chow (10 kcal % fat) diet were used as the baseline control. To exclude the possible effects of DMSO, we tested the mice injected with DMSO-containing versus non DMSO-containing vehicle and observed no significant difference (Supplementary information, Fig. S6b, c).

In the db/db mice, the injection of C3 or C4 significantly reduced the whole body weight gradually compared to the DMSO injection, causing ~15% weight loss within two weeks (Fig. 6a). Such effects were not observed in any control group (Fig. 6a). The fat/lean ratio and liver weight were also significantly reduced (Fig. 6a). Consistent with these observations, the TAG and TC levels in the mouse livers and sera were significantly reduced, reaching a level comparable to those of the WT controls (Fig. 6b, c), confirming the effects of these compounds in vivo. Such effects were unlikely due to changes in food and water consumption, because the weight-normalized food and water intake was not significantly decreased by C3 or C4 injections (Supplementary information, Fig. S6d). The FFA levels were also significantly lowered in the serum and liver (Supplementary information, Fig. S6e, f). Consistent with the biochemical assay results, visualization of liver LDs by BODIPY493/503 staining confirmed a significant decrease in the LD number and size by treatment of C3 or C4 (Fig. 6d).

To obtain a more complete spectrum of changes of lipids by these compounds, we performed the absolute quantitative lipidomic analysis of the liver samples of the tested animals (see Supplementary information, Data S3). The total lipid concentration in the liver was lowered in the C3 and C4 groups by 62.0% ± 23.2% and 41.5% ± 13.9%, respectively (calculation was based on the sum of detected lipids in Supplementary information, Data S3). C3 or C4 injections lowered most glycerolipids, sterol lipids, and neutral sphingolipids (Fig. 6e, left panel, see dashed cyan boxes: darker red or brighter green indicates lower levels). This observation is consistent with the predicted mechanism that LD·ATTECs tether neutral lipids or LDs with LC3 to target them for autophagic degradation (Fig. 1b). In general, LD·ATTECs did not lower polar lipids such as glycerophospholipids (Fig. 6e, left panel), and this is consistent with the fact that the LD·ATTECs did not impair intracellular or cellular membrane integrity (Supplementary information, Fig. S5). We then analyzed different lipid classes (see the X-axis of the right panel, Fig. 6e), and calculated log2 fold changes (log_2_FC) and statistical significance (*P* values) compared to the DMSO group. The TAG concentration was significantly lowered by C3 or C4, but not SIII (Fig. 6e, right panel). Other major neutral lipid classes such as cholesterol ester (ChE) and diglyceride (DG) were also significantly lowered by C3 and somewhat lowered by C4, but not SIII (Fig. 6e, right panel). The rest of lipid classes were either unchanged or had very low abundance (illustrated by small symbol size). Noticeably, the low-abundance lipid classes including lysophosphatidylethanolamine (LPE), lysophosphatidylinositol (LPI), lysophosphatidylglycerol (LPG), sphingomyelin (SM) and sphingosine (So) were increased in the C3-treated group (Fig. 6e, right panel). The increase of these lipid classes could be due to secondary metabolic effects or partial degradation of other lipids, and the mechanisms remain to be further studied. In addition, the abundance of these lipids is relatively low, and thus the measurement could be less accurate compared to the high-abundance lipid classes. We performed a detailed analysis of the lipidomics data focusing on a few key exemplar lipids including the neutral lipids TAG and ChE, as well as the polar lipids phosphatidylethanolamine (PE) and phosphatidylinositol (PI). All TAG and ChE with different chain carbon numbers or saturated bond numbers were lowered by C3 or C4, whereas PE and PI were not affected (Supplementary information, Fig. S7a, b).

Besides the lipids, LD·ATTECs may influence proteins through lowering LDs or interacting with other proteins. We performed proteomic analyses to investigate which proteins were influenced by treatment of LD·ATTECs in vivo using the liver tissues from injected mice, and observed significantly lowering of the LD marker protein Plin2 by treatment of LD·ATTECs C3 or C4, but not SIII (Supplementary information, Fig. S8 and Data S4). While several Plin family proteins are present in the LDs, Plin2 is the only constitutive and ubiquitously expressed protein that has been used as a protein marker for LDs.^28^ None of lipid synthetases or lipases or their cofactors were significantly changed. Autophagic substrates or core pathway genes were not affected either (Supplementary information, Fig. S8b and Data S4). Thus, the LD-lowering effects were unlikely due to altered lipid synthesis/hydrolysis and/or global autophagy. Interestingly, the protein changes induced by the two LD·ATTECs showed substantial overlap including changes of 8 proteins that were not changed by SIII (Supplementary information, Fig. S8b, Venn diagram, and Data S4). Some of these protein changes may represent the outcome of LD lowering. For example, Gstm6 and Gstp1 are downregulated in livers from mice treated with intravenous lipid emulsions or fed with high-fat diet,^29,30^ and were upregulated in LD·ATTEC-treated groups, possibly as a consequence of LD lowering. Thus, LD·ATTECs did not directly perturb protein levels on the whole, but indirect and cascading effects on protein levels cannot be entirely ruled out.

**Fig. 7:**
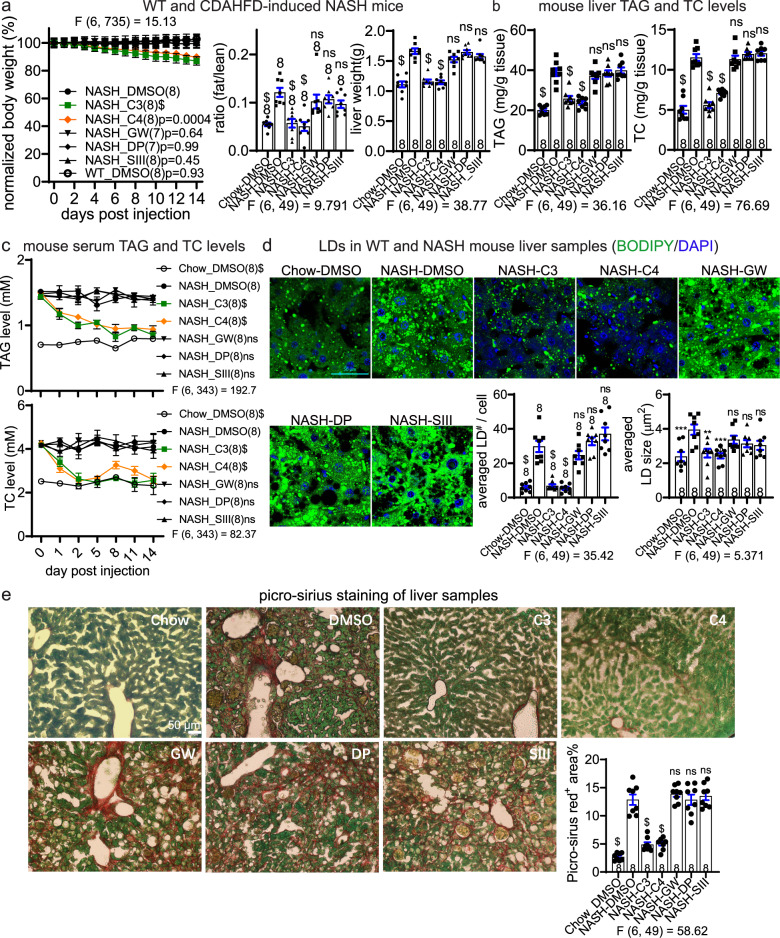
LD·ATTECs were effective in vivo in CDAHFD-induced NASH mice. Similar to Fig. 6, but using the CDAHFD-induced NASH mice. Note that the same group of WT mice fed on chow diet (WT) were used as the baseline control. **a** Measurements of body weight (measured each day and normalized to the averaged weight of day 0, left panel), the fat/lean ratio (after injection for 12 days, middle panel), and liver weight (endpoint measurement after injection for 14 days, right panel) in the indicated mice injected with the indicated compounds. **b** The endpoint measurements (after injection for 14 days) of the TAG and TC levels of liver samples in the indicated mice injected with the indicated compounds. **c** Measurements of the serum TAG and TC levels in NASH mice injected with the indicated compounds. Serum was collected at several different time points after injection as indicated. **d** Representative images and quantifications of the BODIPY493/503 staining of the endogenous LDs in the liver slice samples from the mice injected with the indicated compounds for 14 days. Scale bar, 50 μm. **e** Representative images and quantifications of picro-sirius staining to evaluate the interstitial fibrosis in the liver samples from the mice injected with the indicated compounds for 14 days. The red^+^ area normalized to green^+^ area was quantified to evaluate the degree of liver fibrosis. For all plots, data were plotted as means ± SEM. The replicate number indicates the number of mice. For measurements at multiple time points, two-way ANOVA and Dunnett’s post hoc tests (compared to the DMSO-treated NASH group) were performed. For endpoint measurements for multiple groups, one-way ANOVA and Dunnett’s post hoc tests (compared to the DMSO-treated NASH group) were performed. F and degree of freedom values have been indicated, and exact *P* values are shown if space allows.

Consistent observations were made in the NASH mice induced by feeding with CDAHFD (Fig. 7). Similar to the results obtained from db/db mice, the body weight loss induced by LD·ATTECs was evident (Fig. 7a). The fat/lean ratio and the liver weight were also significantly lowered to a level close to the chow group by injection of LD·ATTECs (Fig. 7a; note that the chow group is the same as the WT group in Fig. 6). At the molecular and cellular level, the serum and liver TAG and TC levels were also lowered (Fig. 7b, c), and the liver LDs were significantly reduced (Fig. 7d). These NASH mice also develop liver fibrosis, which was further evaluated by picro-sirius staining (see “Material and Methods”). Interstitial fibrosis in the liver tissues was significantly alleviated by C3 or C4 compared to the control compounds (Fig. 7e), further suggesting potential beneficial effects of LD ∙ ATTECs.

Taken together, these data demonstrate the in vivo efficacy of LD·ATTECs in two different mouse models.

## Discussion

We have demonstrated the LD-lowering effects of LD·ATTECs, which are designed by attaching an LC3-binding compound to an LD detection probe, such as SIII or SIV (Fig. 1). These LD·ATTECs tether LDs to autophagosomes (Figs. 4, 5) and enhance their subsequent degradation via autophagy (Figs. 2, 3, 6 and 7). The LD-binding moieties of our designed LD·ATTECs are SIII or SIV, which were previously used as food additives and later banned due to safety concerns.^31^ The SIII and SIV analog, Sudan Red dye Sudan I (SI), was associated with a significant increase in neoplastic nodules in both male and female rats, while there were no substance-related clinical signs of toxicity or cases of death.^32^ Thus, prolonged high-concentration treatment of our designed LD·ATTECs may have safety concerns that need to be addressed before any clinical use. Meanwhile, our dosage (30 mg/kg for 14 days) is well below the potentially carcinogenic dosage of SI (60 or 120 mg/kg for 103 weeks), and thus the chance of inducing tumor under our experimental conditions is extremely low. In fact, the compound concentrations were relatively high in the liver and white tissues after injection for 14 days in the NASH mice (Supplementary information, Fig. S9), suggesting that injection at lower concentrations may still work. Our current study is yet a proof of concept, and the LD-binding moiety of LD·ATTECs could be modified with other safer LD-detecting probes,^17^ or even analogs of small-molecule neutral lipids such as cholesterol or steroid, which may enter LDs and recruit LC3 to enhance engulfment of LDs by autophagosomes.

Degradation of LDs via autophagy may produce FFA through hydrolysis of neutral lipids^6,33^; however, LD·ATTECs actually lowered FFA levels (Supplementary information, Fig. S6e, f), likely because the produced FFAs were consumed via mitochondria to provide energy or undergo efflux before reincorporation into cellular LDs, as suggested by recent studies.^34^ Consistent with this, previous studies also demonstrated that forced lipophagy led to decreased LD numbers and sizes.^35^ In addition, we observed lowered FFA levels and increased β-oxidation in the cells after treatment with LD·ATTECs, whereas the FFA level in the culture medium was not significantly influenced (Supplementary information, Fig. S10), suggesting that the LD·ATTEC-induced hydrolysis of LDs may enhance FFA consumption.

It is important to explore the impact of linker length on ATTEC efficacy. We have synthesized 24 additional LD·ATTECs with different linkers and/or warhead, and we are in the middle of testing their efficacies. Whether LD-ATTECs have lower or higher requirements in linkerology design would provide useful insights into the development of different ATTECs.

In a broader spectrum, lowering non-protein pathogenic substrates is highly desired as a potential therapeutic strategy, but has been extremely challenging. A recent study presented the autophagy-targeting chimera (AUTAC) system, which triggers K63 ubiquitination of the target protein and induces its degradation via selective autophagy.^36^ The authors successfully utilized AUTACs to degrade mitochondria by targeting the fused tag of an exogenously expressed mitochondrial outer membrane protein.^36^ While AUTAC has the potential of degrading organelles, it still requires a target protein because it is dependent on K63 ubiquitination of the protein. In the published study, an artificial protein had to be used as the target for mitochondria degradation. In comparison, ATTECs directly tether the target to autophagosomes for degradation via a ubiquitination-independent mechanism and are able to target endogenous non-protein targets directly in cells and in vivo (Figs. 2, 3, 6 and 7).

Our study demonstrates the concept of degrading these targets selectively via autophagy by designed chimeric ATTECs. Autophagy is a powerful cellular degradation machinery capable of degrading proteins as well as many types of non-protein substrates such as lipids, organelles, and nucleic acids.^6,37,38^ Conceptually, all autophagy substrates could be targeted for degradation by designed ATTECs,^39^ opening a new window in biomedical research.

## Material and Methods

### Cell lines and 3T3-L1 differentiated adipocytes

Atg5^+/+^ (WT) and Atg5^–/–^ (Atg5 KO) MEFs were kindly provided by Dr. N. Mizushima.^19^ The SH-SY5Y cells were originally obtained from American Type Culture Collection (ATCC, Cat# ATCC® CRL-2266™, RRID: CVCL_0019), 3T3-L1 (ATCC, Cat# ATCC® CL-173™, RRID: CVCL_0123). The WT and LC3B homozygous knockout HEK293T cells were purchased from ABclonal Technology Inc. (Cat# RM09015) and validated by western blot. All the mammalian cell lines were cultured in DMEM (Life Technologies, Cat# 11965) with 10% (vol/vol) FBS (Life Technologies, Cat# 10099-141) and were maintained in a 37 °C incubator with 5% CO_2_. The cells were tested every two months by a TransDetect PCR Mycroplasma Detection Kit (Transgen Biotech, Cat# FM311-01) to ensure that they are mycoplasma free.

For 3T3-L1 differentiated adipocytes, the 3T3-L1 cells were differentiated into adipocytes according to the protocol described everywhere. Briefly, for preadipocyte differentiation stage, confluent cultures of 3T3-L1 cells were exposed to the induction medium containing 0.5 mM 3-isobutyl-1-methylxanthine (Sigma-Aldrich, Cat# I5879), 0.25 μM dexamethasone (Sigma-Aldrich, Cat# D4902), 50 μg/mL insulin (YEASEN Biotech, Cat# 40107ES25) (Day 0). After 48 h (Day 2), the medium was changed to mature medium containing 5 μg/mL insulin for 48 h (Day 4). On day 7, the medium was changed to normal culture media until the cells achieved the mature adipocyte phenotype. Cells were fully differentiated on days 10–12 and then employed for the following experiments.

### Commercially available compounds and compound treatment in cells

Commercially available compounds were purchased as follows: DMSO (Sigma-Aldrich, Cat# D2650), NH_4_Cl (Sigma-Aldrich, Cat# A9434), Bafilomycin A1 (Sigma-Aldrich, Cat# B1793), GW5074 (Selleck, Cat# S2872), Linker (Sigma-Aldrich, Cat# 53463-68-6), DP (Sigma-Aldrich, Cat# 578320), Sudan III (TCI Chemicals, Cat# S0142), Sudan IV (Admas, Cat# 86726 A), OA (Sigma-Aldrich, Cat# O7501), BODIPY 493/503 (Thermo Fisher Scientific, Cat# D3922). Unless otherwise stated, the compounds were diluted into 10× solution using the culture medium, added to the culture medium and incubated for 24 h. For starvation experiments, the cells were treated with Earle’s Balanced Salt Solution (EBSS) for 4 h before adding the indicated compounds.

### Protein extraction and western blot

The cell pellets were collected and lysed on ice for 30 min in 1× PBS (HyClone, Cat# SH30256.01) + 1% Triton X-100 + 1× complete protease inhibitor (Roche, Cat# 04 693 132 001), sonicated for 10 s, and spun at > 20,000× *g* at 4 °C for 10 min. The supernatants were then loaded and transferred onto nitrocellulose membranes for western blot. Commercially purchased antibodies include anti-SQSTM1/p62 (Abcam, Cat# ab56416, RRID: AB_945626), anti-β-tubulin (Abcam, Cat# ab6046, RRID: AB_2210370), anti-Atg5 (ProteinTech, Cat# 10181-2-AP, RRID: AB_2062045), anti-LC3B for western blot (Thermo Fisher Scientific, Cat# PA1-16930, RRID: AB_2281384), anti-LC3B for immunofluorescence (Thermo Fisher Scientific, Cat# 700712, RRID: AB_2532340). For immunofluorescence assay, LC3B was probed with Dylight 594 donkey anti-rabbit (Jackson ImmunoResearch Lab, Cat# 711-517-003, RRID: AB_2340617). The primary antibodies were detected by AffiniPure peroxidase-conjugated secondary antibodies (Abmart, goat anti mouse IgG HRP, Cat# m21001L, RRID: AB_2713950; goat anti rabbit IgG HRP, Cat# m21002L, RRID: AB_2713951). The blots were developed with SuperSignal™ West Pico PLUS Chemiluminescent Substrate (Thermo Fisher Scientific, Cat# 34580). The specificity of all antibodies has been validated by previous reports or our own knockdown or knockout experiments. The signal intensity of each band was quantified by ImageJ.

### cDNA plasmids and siRNA transfection

The cDNA plasmids mCherry-LC3B (Cat# 40827), mRFP-GFP-LC3B (Cat# 21074), and Lamp1-mCherry (Cat# 45147) were purchased from Addgene. All plasmids were validated by sequencing. For transient transfections, the cells were plated at 60%–70% confluence. After 12 h, the cDNAs were transfected with Lipofectamine 2000 (Thermo Fisher Scientific, Cat# 11668019) using the forward transfection protocol provided by the manufacturer. The siRNA target sequences are as follows: non-targeting control siRNA, 5′-UUCUCCGAACGUGUCACGUTT-3′; human ATG5_siRNA, 5′-GCCUGUAUGUACUGCUUUA-3′; mouse Atg5_siRNA, 5′-CUCUCUAUCAGGAUGAUTT-3′; all the siRNAs have been validated by western blot showing > 80% knockdown of the target.

### Induction of LDs by extracellular OA

The MEFs or the SH-SY5Y cells were plated at 50% confluence and incubated for 24 h before induction. 20 mM OA solution was prepared by adding 18 mg OA into 3 mL MilliQ water and incubating for 10 min at 75 °C to dissolve OA. 20% or 10% BSA solution was made by dissolving BSA (Sigma-Aldrich, Cat# B2064-50G) in 3 mL tissue culture grade distilled water (Thermo Fisher Scientific, Cat# 15230-162). 3 mL 20% BSA was then added to 3 mL of 20 mM OA solution to obtain 10 mM OA-BSA solution. The 10% BSA and 10 mM OA-BSA solution was then filtered by the membrane with 0.22 μm pore size (Milipore, Cat# SLGV033RB). Then OA-BSA solution at a final concentration of 200 μM was used to induce LDs in cells, and 10% BSA was used as the control. Fresh solutions were prepared for every induction assay.

### LD detection by BODIPY 493/503

For cultured cells, they were seeded at 5 × 10^4^ cells on 0.1% gelatin (Sigma-Aldrich, G1890) coated glass coverslips (VWR, Cat# 631-0149) and cultured in 90% DMEM + 10% FBS with 5% CO_2_ for 12 h. Following the indicated transfections and/or compound treatments, cells were fixed in 4% paraformaldehyde (PFA; Sinopharm Chemical Reagent, Cat# 80096628) for 10 min, washed three times in 1× PBS and incubated in PBS with BODIPY 493/503 (1:10,000 from a 1 mg/mL stock solution in DMSO; Thermo Fisher, Cat# D3922) for 15 min and DAPI (1:1000; Beyotime Biotechnology, Cat# C1002) for 10 min at room temperature. Cells were washed twice in 1× PBS and mounted on microscope slides with Vectashield (Vector Laboratories, Cat# H-1000). The number and size of LDs were analyzed by ImageJ software in a blinded manner.

For imaging of LC3B-mCherry- and mRFP-GFP-LC3B-transfected cells, cultured cells were fixed in 4% PFA after being washed with 1× PBS for three times, and then permeablized in 0.5% Triton X-100 for 10 min. The cells were washed three times, stained with DAPI (1:1000; Beyotime Biotechnology, Cat# C1002), and then mounted in the vectashield mounting medium (Vector, Cat# H-1002). For immunofluorescence, cultured cells were fixed and washed in the same way as above. The cells were then blocked with 4% BSA + 0.1% Triton X-100 in 1× PBS for 30 min and incubated overnight at 4 °C with primary antibody, goat polyclonal Lamin B1 (1:200, Santa Cruz Biotechnology, Cat# sc-6216, RRID: AB_648156), and then washed three times with blocking buffer and incubated with secondary antibody (1:500, Alexa Flour488 donkey anti-goat, Jackson immunoResearch Lab, Cat# 705-547-003, RRID: AB_2340431) at room temperature for 1 h. The samples were then washed three times, stained with DAPI for 10 min at room temperature, and then mounted in the vectashield mounting medium.

For tissue BODIPY staining, the experiments were performed similarly as described.^40^ Basically, liver was removed and weighed, dissected into parts, and then immediately fixed with 4% PFA for 48 h at 4 °C. The tissues were incubated in 15% sucrose for ~24 h and then in 30% sucrose for ~48 h at 4 °C, and then liver was frozen with OCT compound (Thermo Fisher, NEG50^TM^, Cat# 6502). 15 μm thick liver cryosections were prepared and mounted on glass slides, and set on room temperature for 10 min. The cryosections were then rapidly immersed into ice-cold 4% PFA in 1× PBS for 1 h for fixation. Right after fixation, the cryosections were then washed with 1× PBS and incubated for 30 min with BODIPY 493/503 (1:5000 from a 1 mg/mL stock solution in DMSO; Thermo Fisher, Cat# D3922) and DAPI (1:1000; Beyotime Biotechnology, Cat# C1002) for 10 min at room temperature. Subsequently, the cryosections were washed in cold 1× PBS and mounted in Vectashield.

All the images were taken at 63× magnification using a confocal scanning laser microscope (LSM 880, Zeiss) with LSM software. The number and size of LDs were analyzed by ImageJ software in a blinded manner.

For LD measurements using the Incucyte technology (Essen Bioscience, IncuCyte S3), the images were automatically taken for each well every 0.5 h inside the incubator. The quantification was performed by the Incucyte Analyzer software, which identified the green fluorescent puncta and quantified total green^+^ area as the total LD area, and quantified cell confluence based on the phase-contrast images.

### Picro-sirius red staining

Fifteen micrometer cryosections were obtained from the liver (see “LD detection by BODIPY 493/503 — for tissue BODIPY staining”), and processed for visualization of fibrosis by picro-sirius red staining as described previously.^41^ The liver cryosections were washed with 1× PBS and then incubated at room temperature for 1 h with the solution containing 0.1% vol/vol Direct Red 80 (Sigma, Cat# 365548, for staining collagen in red as an indicator for liver fibrosis) and 0.04% Fast Green (Sigma-Aldrich, Cat# F7258, for general protein staining as a background) in saturated aqueous solution of picric acid (Sigma, Cat# P6744-1GA). The samples were imaged using an Olympus inverted fluorescence microscope IX73, and the histological fibrosis images were analyzed by the ImageJ software in a blinded manner.

### Cellular staining using LysoTracker, MitoTracker or CellMask

For LysoTracker staining, the medium was removed from the dish and the prewarmed (37 °C) probe-containing culture medium (500 nM, LysoTracker™ Green DND-26, Thermo Fisher, Cat# L7526) was added, the cells were incubated for 3 h, and then Hoechst 33342 (Thermo Fisher, Cat# H3570) was added to incubate for 5 min. The staining solution was removed and the coverslip was rinsed with Live Cell Imaging solution (Thermo Fisher, Cat# A14291DJ) three times. The slide was mounted with the coverslip and the image was immediately acquired using a confocal scanning laser microscope (LSM 880, Zeiss).

MitoTracker staining and CellMask staining were performed in the same way as LysoTracker^TM^ staining, except incubating with MitoTracker™ Green FM (500 nM, Thermo Fisher, Cat# M7514; detecting total mitochondria) and MitoTracker™ Red CMXRos (500 nM, Thermo Fisher, Cat# M7512; detecting healthy mitochondria) for 3 h, or CellMask^TM^ (2000×, CellMask™ Plasma Membrane Stains, Thermo Fisher Scientific, Cat# C10046) for 5 min at 37 °C. The CellTiter-Glo® luminescent cell viability assay was performed to measure cell viability with the indicated compound treatment following the protocol provided by the kit (Promega, Cat# G7572).

### Expression and purification of the GST-LC3B recombinant proteins

The protein was prepared as described previously.^8^ Basically, the human microtubule-associated protein 1 light chain 3-β (*MAP1LC3B* (LC3B)) gene (GenBank: NM_022818.4) was amplified by PCR and cloned into a pGEX-6P1 (GE Healthcare) derived vector pGHT, which is a prokaryotic expression vector reconstructed by adding a His8 tag and a TEV protease cleavage site before the pGEX-6P1 multiple cloning site. After sequencing verification, the expression plasmid pGHT-LC3B was introduced into *Escherichia coli* BL21 (DE3) pLsyS, in which the recombinant GST-LC3B protein was expressed by induction with IPTG. When the bacterial culture reached OD600 = 0.8, its temperature was decreased to 18 °C, and 0.2 mM IPTG was added into the culture for an additional 20 h incubation. The cells were then harvested by centrifugation (6000× *g*, 4 °C, 15 min) and the cell pellet was suspended in 50 mM Tris-HCl buffer, pH 7.5, with 150 mM NaCl and 5% glycerol. Cells were then disrupted by sonication, followed by centrifugation (20,000× *g*, 4 °C, 60 min). The supernatants were then loaded onto a HisTrap HP column (GE Healthcare, Cat# 17524701), and eluted with 50 mM Tris-HCl buffer, pH 7.5, containing 150 mM NaCl, 5% glycerol and 300 mM imidazole. The LC3B eluate was then mixed with TEV protease (Sigma-Aldrich, Cat# T4455; eluted protein:TEV protease = 100:1) and dialysed against the dialysate buffer (50 mM Tris-HCl buffer, pH 7.5, containing 100 mM NaCl) at 4 °C overnight. After TEV protease treatment, the samples were then loaded onto a HisTrap HP column again, and the flow-through fraction which mainly contains tag-removed recombinant LC3B was collected. Afterwards, the proteins were concentrated and further purified by Superose 6 Increase 10/300 GL (GE Healthcare) size-exclusion chromatography. Finally, the purified proteins were concentrated to ~10 mg/mL in 50 mM HEPES buffer with 100 mM NaCl for further analysis.

### Compound–protein interaction measurements by MST

The purified recombinant proteins were dialysed into 1× PBS, and then labeled with the red fluorophore according to the protocol of Protein Labeling Kit RED-NHS (Nanotemper, Cat# L001). All the tested stock compounds (25 mM) were serially diluted into the same buffer (20 mM HEPES, pH 7.5, 150 mM NaCl) with the same final DMSO concentration (2.5%) for the MST assay. The MST experiment was performed using Monolith NT.115 instrument (NanoTemper Technologies). Labeled proteins (500 nM) were mixed with the indicated concentrations of candidate compounds in reaction buffer containing 20 mM HEPES, pH 7.4, 150 mM NaCl. The MST data were then collected under 40% infrared laser power and 20% light-emitting diode power. The data were analyzed by Nanotemper analysis software (v.1.5.41).

### TAG–compound–LC3B ELISA assay

The assay was performed using the Mouse Triglyceride ELISA Kit (Mybiosource, Cat# MBS2516318) based on the manufacturer’s protocol with some modifications. Briefly, the assay plate was pre-coated with an antibody specific mouse TAG. The wash buffer in the kit was added to each well and incubated for 5 min, and then the solution was aspirated. 100 μL TAG-containing solution (30 μL of 3 μg/mL TAG pre-dissolved in the standard solution + 70 μL ddH_2_O) was immediately added to each well. The plate was covered, gently vortexed and then incubated for 1 h at 37 °C. The solution was aspirated from each well, and then 250 μL wash buffer was added to each well. The plate was soaked for 1–2 min and the solution was aspirated or decanted from each well. This wash step was repeated 3 times. 100 μL of the indicated compounds (5 μM for C1–C4, and DMSO as control) diluted in the working solution was immediately added to each well, and then incubated for 1 h at 37 °C. The plate was washed with 250 μL wash buffer 3 times and 100 μL of HRP-conjugated anti-GST antibody (1:2000 in working solution, ProteinTech, Cat# HRP-66001) was added to each well. The plate was covered and then incubated for 30 min at 37 °C. The solution was aspirated or decanted from each well, and then each well was washed for 5 times using 250 μL wash buffer each time. 90 μL of Substrate Reagent was added to each well, and the plate was covered and then incubated for up to 10 min at 37 °C protected from light. The reaction time can be shortened according to the actual color change, but not more than 10 min. 50 μL of stop solution was added to each well in the same order as adding the substrate solution to ensure identical reaction time. The optical density (OD value) of each well was determined with a microplate reader set to 450 nm (BioTek Synergy 2).

### Compound measurements in vivo in liver tissue from i.p. injected mice

The experiments were performed by the SIM-Servier joint laboratory. The male mice, i.p. injected with the indicated compounds, were anesthetized by chloral hydrate (200 μL/kg of 10% stock) at the indicated time points. The heart and portal vein blood was collected by vacuum blood collection tubes. The heart blood samples were further spun at 10,000 rpm for 5 min to generate the heart plasma. The mice were then perfused with 1× PBS to remove the blood. The mice were then euthanized, and the liver samples were dissected. Five times the volume of methanol:acetonitrile (50:50, vol/vol) were added to each sample, which was then homogenized. Following ultrasonic treatment for 15 min, the homogenates were centrifuged for 5 min, and then 20 μL supernatant was mixed with 20 μL water for 30 s before injection. The LC-MS/MS analyses were performed on an Acquity ultra performance liquid chromatography (UPLC) system (Waters Corporation) coupled to a Xevo TQ-S mass spectrometer (Waters Corporation). Chromatographic separation was performed using an Acquity UPLC BEH C18 (1.7 μm, 2.1 mm × 50 mm) column supplied by Waters at a flow of 0.5 mL/min. Gradient elution was used with a mobile phase composed of solvent A (water containing 0.1% formic acid and 5 mM NH_4_Ac) and solvent B (acetonitrile:methanol (9:1, vol/vol) containing 0.1% formic acid). SIM-Servier joint laboratory measured compound distribution in the tissues after 14-day injection.

### Animal experiments for db/db and NASH mouse models

Mice were maintained at the university mouse facility. Mice were group-housed (up to 5 adult mice per cage) in individually vented cages with a 12 h light/dark cycle. The mouse experiments were carried out following the ARRIVE (Animal Research: Reporting of In Vivo Experiments) guidelines, and followed all relevant ethical regulations. The protocol used in animal experiments was approved by The Animal Care and Use Committee of Shanghai Medical College of Fudan University (Approval #202004001S).

For db/db mouse experiments, 19-week-old db/db male (C57BL/6J-Lepr^db^/Lepr^db^) mice^26,42^ were obtained from Shanghai Model Organisms (Shanghai, China) and acclimatized for at least 1 week before experiments. The mice were on ad libitum access to standard chow and water. The mice were randomly divided into seven groups with 8 mice in each group for i.p. injections: (1) vehicle alone (no DMSO) group: 0% DMSO + 39% PEG300 (Selleck, Cat# S6704) + 5% Tween-80 (Selleck, Cat# S6702) + 56% distilled H_2_O, (2) DMSO group: 1% DMSO + 39% PEG300 + 5% Tween-80 + 55% H_2_O (DMSO vehicle), (3) C3 group: LD·ATTEC3 in DMSO vehicle, (4) C4 group: LD·ATTEC4 in DMSO vehicle, (5) SIII group: Sudan III in DMSO vehicle, (6) GW group: GW5074 in DMSO vehicle, (7) DP group: DP in DMSO vehicle. For groups 3–7, compounds (150 μL) were injected to reach 30 mg/kg for each mouse. Compounds were administered once a day by i.p. injections for two weeks. Food intake and water intake was weighted by analytical electronic balance. One mouse in the GW group and one mouse in the DP group died in the middle of experiments, and thus were excluded.

For NASH mouse models, 10-week-old C57BL/6 male mice were purchased from Hangzhou Ziyuan Inc., and provided with ad libitum access to water and CDAHFD containing 60% kcal fat purchased from Shuyishuer Inc (Shuyishuer, Cat# A06071302) for 12 weeks (including the two-week compound injection period) to generate the NASH model.^43^ The experimental design and group assignment were performed in the same way as the db/db mice. For both the db/db and NASH mice, the C57BL/6 male mice fed standard irradiated chow diet (Shuyishuer Inc., Cat# D12450J) were used as the normal control. Blood (30–50 μL) was collected via tail vein every 1–3 days whenever time allows. 24 h after injection at the final time point, all mice were euthanized using cervical dislocation, after which the tissue samples were acquired and stored in Eppendorf tubes at –80 °C until assay.

For NMR-based measurements of lean versus fat mass, each mouse was scanned with a minispec NMR instrument (Bruker LF50 II “Minispec” body composition analyzer, Bruker Optics) designed for experimental animals, and the fat versus lean content was determined by the device based on the measurements of the solid and liquid parts of the sample.

### Measurements of TAG, TC, and FFA levels in cell culture, medium, serum and liver tissue samples

The cultured cells were collected and lysed by 1% Triton X-100 in 1× PBS with 1× protease inhibitor. Whole blood samples were collected via tail vein (30–50 μL), the blood was allowed to clot by leaving it undisturbed at room temperature in the EDTA K2 blood collection tubes (Jiangsu Kangjian Medical Apparatus, Cat# KJ002). The clot was removed by centrifuging at 2000× *g* for 10 min at 4 °C and the supernatant was collected, which is the serum. For liver samples, the livers were dissected and 100 mg of liver tissue was homogenized in 0.9 mL ice-cold absolute alcohol, and centrifuged (2500 rpm, 10 min). Supernatants were collected and stored at –80 °C. The TAG, TC, and FFA levels in the serum or liver samples were then measured with the kits according to the manufacturer’s instructions (TAG: TG kit, Cat# A110-1-1; TC: T-CHO kit, Cat# A111-1; FFA: NEFA kit, Cat# A042-2-1; Nanjing Jiancheng Bioengineering Institute). The culture medium for cultured cells was also collected for measurements of FFA. To exclude the potential influence of OA, we replaced the culture medium to remove the extracellular OA after the 6-h induction for the medium FFA measurement. For β-oxidation measurement, Fatty Acid Oxidation Assay (Abcam, Cat# ab217602) allows the detection of endogenous Fatty Acid Oxidation (FAO) in live cells following instructions, and it is designed to be used in combination with the Extracellular Oxygen Consumption Assay (Abcam, Cat# ab197243), and the relative rate change was calculated.

### Absolute quantitative lipidomics

The experiments and data analysis were supported by Shanghai Applied Protein Technology Co., Ltd. Reagents including MS-grade methanol (Cat# A452-4-CASE), MS-grade acetonitrile (Cat# 271004), HPLC-grade 2-propanol (Cat# A461-4-CASE) were purchased from Thermo Fisher. HPLC-grade formic acid (Cat#33015) and HPLC-grade ammonium formate (Cat#70221-100G-F) were purchased from Sigma-Aldrich.

For sample preparation and lipid extraction, lipids were extracted according to MTBE method. Briefly, samples were first spiked with appropriate amounts of internal lipid standards and then homogenized with 200 μL water and 240 μL methanol. After that, 800 μL of MTBE was added and the mixture was treated with ultrasound for 20 min at 4 °C followed by sitting still for 30 min at room temperature. The solution was centrifuged at 14,000× *g* for 15 min at 10 °C and the upper layer was obtained and dried under nitrogen.

For LC-MS/MS method for lipid analysis, reverse phase chromatography was selected for LC separation using CSH C18 column (1.7 μm, 2.1 mm × 100 mm, Waters). The lipid extracts were re-dissolved in 200 μL of 90% isopropanol/acetonitrile, centrifuged at 14,000× *g* for 15 min, and finally 3 μL of sample was injected. Solvent A was acetonitrile–water (6:4, vol/vol) with 0.1% formic acid and 0.1 mM ammonium formate, and solvent B was acetonitrile–isopropanol (1:9, vol/vol) with 0.1% formic acid and 0.1 mM ammonium formate. The initial mobile phase was 30% solvent B at a flow rate of 300 μL/min. It was held for 2 min, and then linearly increased to 100% solvent B in 23 min, followed by equilibrating in 5% solvent B for 10 min. Mass spectra was acquired by Q-Exactive Plus in positive and negative modes, respectively. ESI (Electron Spray Ionization) parameters were optimized and preset for all measurements as follows: source temperature, 300 °C; capillary temperature, 350 °C; the ion spray voltage was set at 3000 V, S-Lens RF Level was set at 50% and the scan range of the instruments was set at 200–1800 m/z.

Identification of lipids was performed by LipidSearch^TM^ software (Thermo Fisher Scientic), which is a widely used search engine for the identification of lipid species based on MS/MS math. LipidSearch^TM^ contains more than 30 lipid classes and more than 1,500,000 fragment ions in the database. Both mass tolerance for precursor and fragment were set to 5 ppm.

### Relative quantitative proteomic analysis

Samples were analyzed on Orbitrap Fusion Lumos mass spectrometers (Thermo Fisher Scientific, Rockford, IL, USA) coupled with an Easy-nLC 1000 nanoflow LC system (Thermo Fisher Scientific). Dried peptide samples were re-dissolved in Solvent A (0.1% formic acid in water) and loaded to a trap column (100 μm × 2 cm; particle size, 3 μm; pore size, 120 Å; SunChrom, USA) with a max pressure of 280 bar using Solvent A, then separated on a 150 μm × 15 cm silica microcolumn (particle size, 1.9 μm; pore size, 120 Å; SunChrom, USA) with a gradient of 5%–35% mobile phase B (acetonitrile and 0.1% formic acid) at a flow rate of 600 nL/min for 75 min. The FAIMS device was placed before the mass spectrometer. FAIMS separation was performed with the following settings: inner electrode temperature = 100 °C, outer electrode temperature = 100 °C, carrier gas flow = 4.6 L/min, dispersion voltage = −5000 V, entrance plate voltage = 250 V. The FAIMS carrier gas is N2 only. The noted CVs were applied to the FAIMS electrodes. Each of the selected CVs was applied to sequential survey scans and MS/MS cycles (1 s); the MS/MS CV was always paired with the appropriate CV from the corresponding survey scan. For detection with Fusion or Fusion Lumos mass spectrometry, a precursor scan was carried out in the Orbitrap by scanning at 300−1400 m/z with a resolution of 120,000. The most intense ions selected under top speed mode were isolated in Quadrupole with a 1.6 m/z window and fragmented by higher energy collisional dissociation (HCD) with normalized collision energy of 30%, then measured in the linear ion trap using the rapid ion trap scan rate. Automatic gain control targets were 5 × 10^5^ ions with a max injection time of 50 ms for full scans and 1 × 10^4^ ions with 35 ms for MS/MS scans. Dynamic exclusion time was set at 18 s. Data were acquired using the Xcalibur software (Thermo Scientific Scientific).

Raw files were searched against the human National Center for Biotechnology Information (NCBI) Refseq protein database (updated on 04-07-2013, 32,015 entries) by Mascot 2.3 (Matrix Science Inc) implemented on Proteome Discoverer 2.2 (Thermo Scientific). The mass tolerances were 20 ppm for precursor and 0.5 Da for product ions for Fusion Lumos. Up to two missed cleavages were allowed. The search engine set cysteine carbamidomethylation as a fixed modification and N-acetylation, oxidation of methionine as variable modifications. Precursor ion score charges were limited to +2, +3, and +4. The data were also searched against a decoy database so that protein identifications were accepted at a false discovery rate (FDR) of 1%. Label-free protein quantifications were performed using a label-free, intensity-based absolute quantification (iBAQ) approach.

Proteins with at least 2 unique peptides with 1% FDR at the peptide level and Mascot ion score greater than 20 were selected for further analysis. The file used for protein inference and protein FDR calculation was derived from Mascot search results, and the peptide spectrum match (PSM) was filtered via Percolator and customized parameters, and then the proteins were assembled. The protein FDR was calculated depending on the ratio of NPD (the number of assembled proteins from decoy database searches) and NPT (the number of assembled proteins from target database searches). The FOT was used to represent the normalized abundance of a particular protein across samples. FOT was defined as a protein’s iBAQ divided by the total iBAQ of all identified proteins within one sample. The FOT was multiplied by 10^5^ for the ease of presentation.

### Statistics

To ensure to reach a statistical power > 0.8, power analyses were performed for each assay based on estimated values by PASS 16 (https://www.ncss.com/software/pass/) before experiments. Estimation was based on our previously published results on similar experiments and preliminary experiments. The effect size was also estimated by Cohen’s d, two means divided by the standard deviation for the data. The power analysis suggested *n* ≥ 5 for LD measurements. In all the experiments we performed, we have used a larger *n* than this. For animal experiments, we used ~8 mice per group based on similar studies published previously, which used 5–7 mice per group.^44^ Unless elsewhere stated, bars represent means ± SEM. Statistical comparisons between two groups were conducted by the unpaired two-tailed *t*-tests. Statistical comparisons among multiple groups were conducted by one-way ANOVA tests and post hoc tests for the indicated comparisons. Statistical comparisons for a series of data collected at different time points were conducted by two-way ANOVA tests. The similarity of variances between groups to be compared was tested when performing statistics in GraphPad Prism 8 and Microsoft Excel 2016. Significance was established at *P* < 0.05.

## Supplementary information

Supplementary information, Fig. S1

Supplementary information, Fig. S2

Supplementary information, Fig. S3

Supplementary information, Fig. S4

Supplementary information, Fig. S5

Supplementary information, Fig. S6

Supplementary information, Fig. S7

Supplementary information, Fig. S8

Supplementary information, Fig. S9

Supplementary information, Fig. S10

Supplementary information, Data S1

Supplementary information, Data S2

Supplementary information, Data S3

Supplementary information, Data S4
