# Supplementary material for: Degradation of lipid droplets by chimeric autophagy-tethering compounds
Source: Cell Res. 2021 Jul 8;31(9):965–79. doi: 10.1038/s41422-021-00532-7 (PMC8410765; doi:10.1038/s41422-021-00532-7)
Supplement: Supplementary file 1 — Supplementary information, Fig. S1 [file 41422_2021_532_MOESM1_ESM.pdf]

Fig.S1

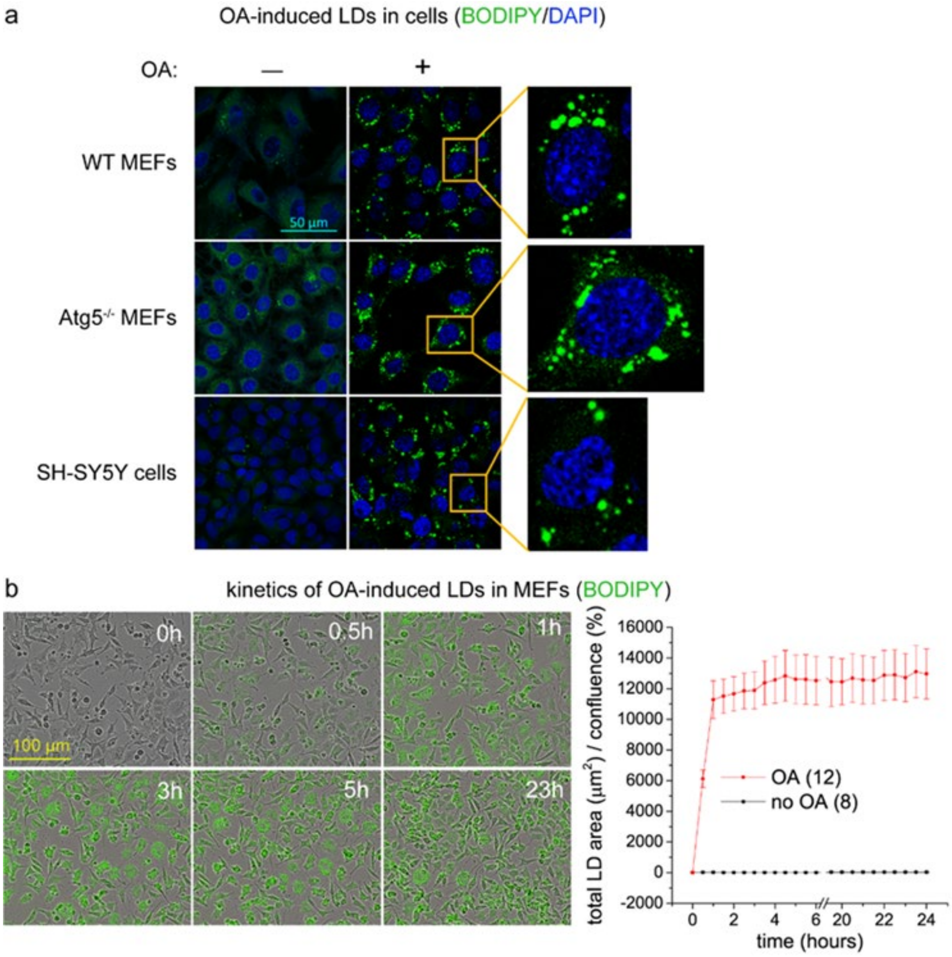

Fig. S1 Induction of lipid droplets by extracellular oleic acid (OA) treatment. a Representative images of BODIPY 493/503 staining results of indicated cells with or without OA (200 μM) induction (6 hours after adding OA versus the control). **b** Representative images and quantifications of OA-induced LDs at different time points in MEFs. LDs formed and reached plateau at ~5 hours after treatment and then remained at the plateau level for at least 24 hours, as detected by the BODIPY 493/503 staining. The images were taken by Incucyte, which is an automated imaging system inside the incubator that can take images at designated time points for each well. Images were taken every 30 minutes. The total LD area (green$^+$) normalized to the cell confluence was further quantified by the Incucyte Analyzer software, which calculated the total area with the green signal above threshold based on the fluorescent imaging and normalize the area to the cell confluence based on the phase-contrast imaging. The n numbers indicate the number of individual wells.   Error bars indicate mean and s.e.m..
