# Supplementary material for: Degradation of lipid droplets by chimeric autophagy-tethering compounds
Source: Cell Res. 2021 Jul 8;31(9):965–79. doi: 10.1038/s41422-021-00532-7 (PMC8410765; doi:10.1038/s41422-021-00532-7)
Supplement: Supplementary file 2 — Supplementary information, Fig. S2 [file 41422_2021_532_MOESM2_ESM.pdf]

Fig.S2

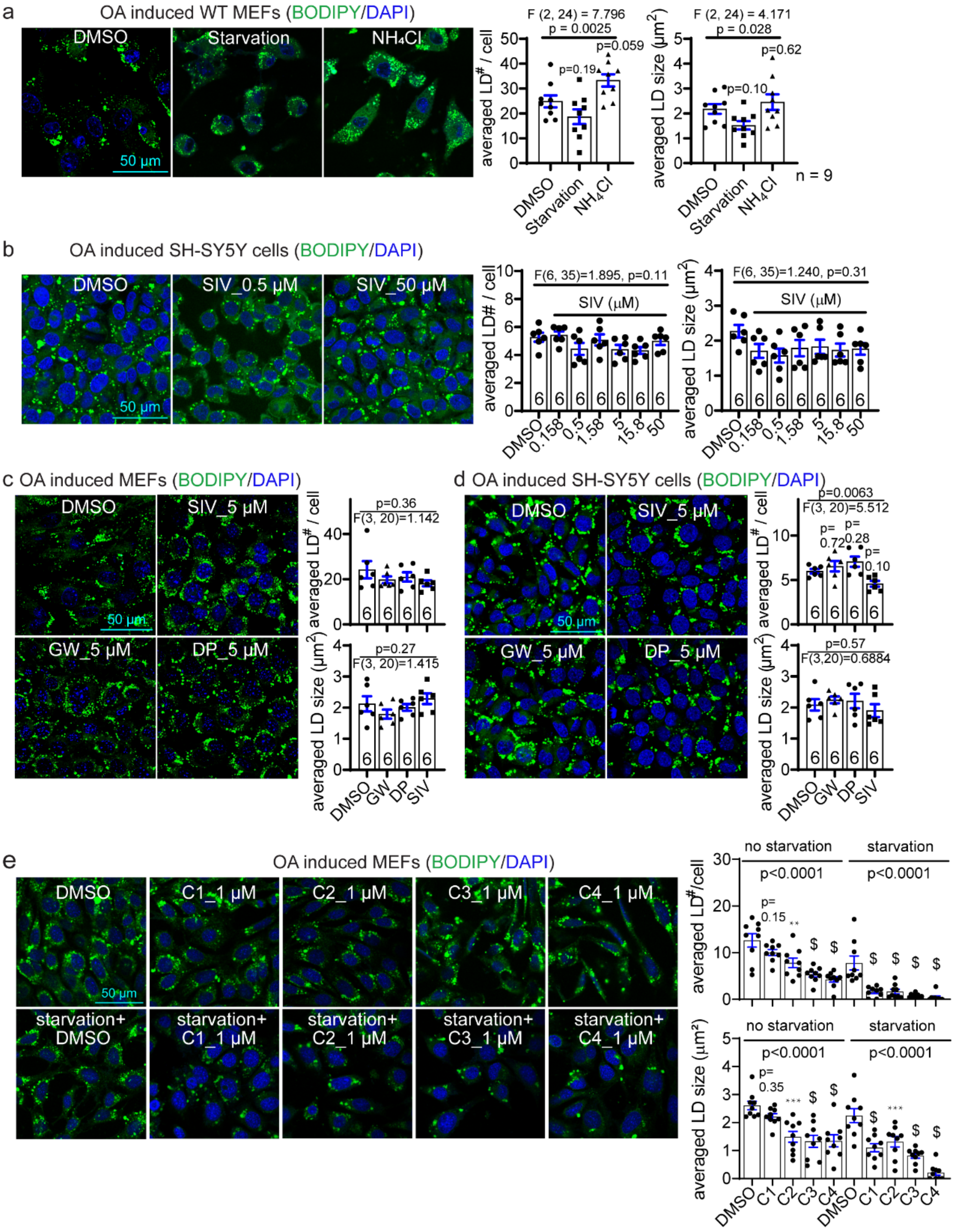
a
OA induced WT MEFs (BODIPY/DAPI)
DMSO
Starvation
NH4Cl
50 µm
F (2, 24) = 7.796
p = 0.0025
p=0.059
p=0.19
averaged LD# / cell
F (2, 24) = 4.171
p = 0.028
p=0.62
p=0.10
averaged LD size (µm2)
n = 9
b
OA induced SH-SY5Y cells (BODIPY/DAPI)
SIV_0.5 µM
SIV_50 µM
F(6, 35)=1.895, p=0.11
SIV (µM)
F(6, 35)=1.240, p=0.31
c OA induced MEFs (BODIPY/DAPI)
SIV_5 µM
GW_5 µM
DP_5 µM
p=0.36
F(3, 20)=1.142
p=0.27
F(3, 20)=1.415
d OA induced SH-SY5Y cells (BODIPY/DAPI)
p=0.0063
F(3, 20)=5.512
p=0.57
F(3,20)=0.6884
e
OA induced MEFs (BODIPY/DAPI)
C1_1 µM
C2_1 µM
C3_1 µM
C4_1 µM
starvation+ DMSO
no starvation
starvation
p<0.0001
p=0.15
p=0.35

Fig. S2 LC3-binding compounds or LD probes alone did not influence BODIPY signals. a Representative images and quantifications of the BODIPY493/503 staining of the OA-induced LDs in wild-type MEFs with the indicated treatments: starvation (medium replacement with EBSS for 4 hours and then in normal medium for 20 hours) to induce global autophagy, or treatment with 10 mM NH_4Cl to inhibit baseline autophagy flux. Note that the treatment was both performed after the 6-hour OA induction of LDs and the DMSO group were from the same ones in Fig. 2a. The ANOVA test results were significant, but the post-hoc analysis did not show significant difference compared to the DMSO group. **b** Representative images and quantifications of the BODIPY493/503 staining of the OA-induced LDs in the SH-SY5Y cells treated with the LD probe SIV at concentrations up to 50 μM, which did not lower the levels of LDs. **c-d** Representative images and quantifications of the BODIPY493/503 staining of the OA-induced LDs in the indicated cells treated LC3-binding moieties or the LD-probe alone. n = 6, independently plated wells. **e** Similar to D, but with or without starvation (medium replacement with the indicated compounds in EBSS for 4 hours, and then changed to normal medium with the same concentration of indicated compounds for 20 hours). n=9 for each group.The LD number per cell and averaged LD size in each field were quantified by ImageJ (particle analysis) in a blinded manner. The statistical analyses were performed by one-way ANOVA (F/degree of freedom/p values have been indicated for each plot) and Dunnett's post-hoc tests (the corrected p values compared to the DMSO group, if the ANOVA tests were significant). The n numbers indicate independently plated wells. **: $p<0.01$, ***: $p<0.001$, $: $p<0.0001$. The exact p values have been indicated if space allows.
