# Supplementary material for: Degradation of lipid droplets by chimeric autophagy-tethering compounds
Source: Cell Res. 2021 Jul 8;31(9):965–79. doi: 10.1038/s41422-021-00532-7 (PMC8410765; doi:10.1038/s41422-021-00532-7)
Supplement: Supplementary file 3 — Supplementary information, Fig. S3 [file 41422_2021_532_MOESM3_ESM.pdf]

Fig. S3

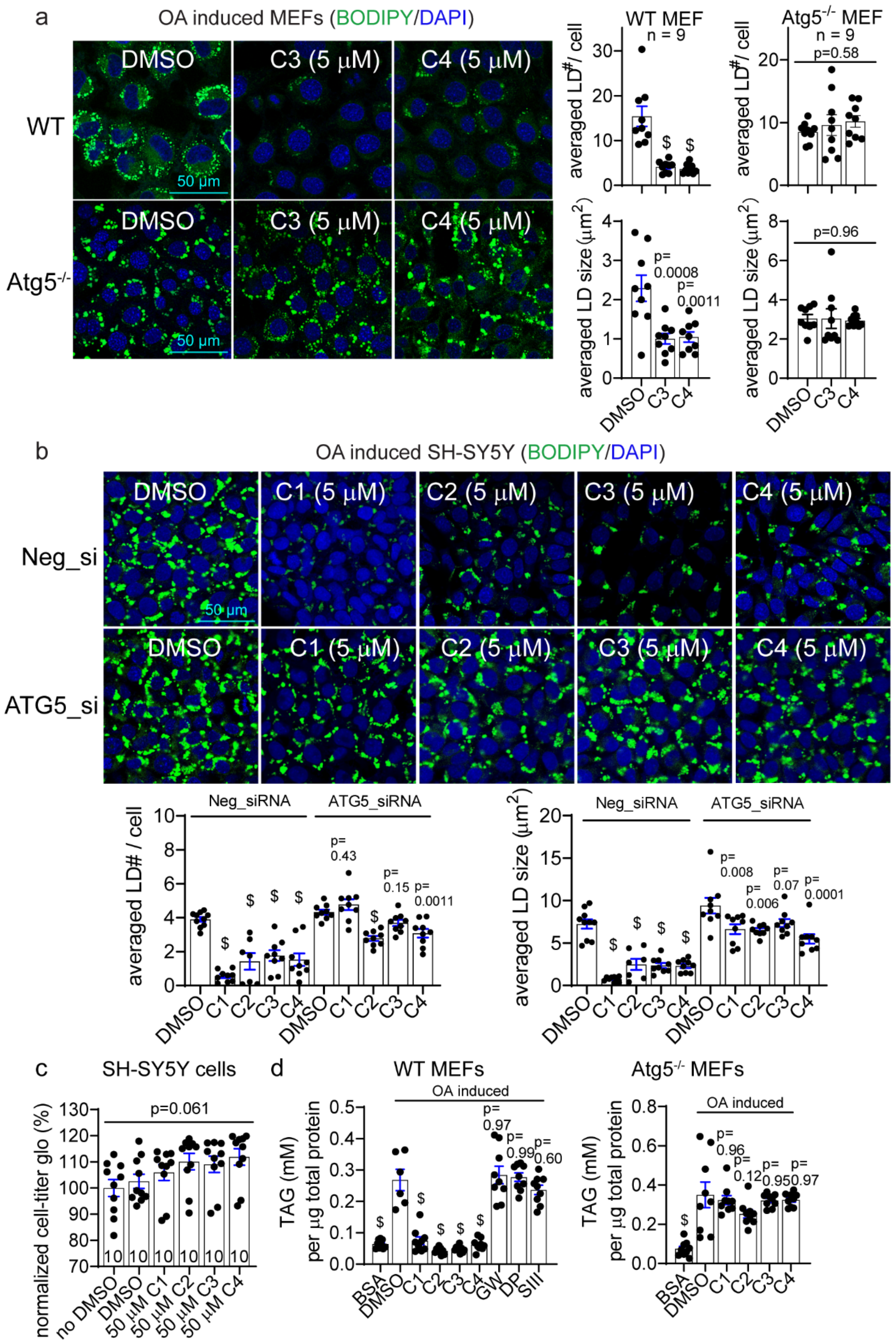
a
OA induced MEFs (BODIPY/DAPI)
DMSO
C3 (5 μM)
C4 (5 μM)
WT
Atg5-/-
50 μm
WT MEF
n = 9
Atg5-/- MEF
p=0.58
averaged LD#/ cell
averaged LD size (μm2)
p= 0.0008
p= 0.0011
p=0.96
DMSO
C3
C4
b
OA induced SH-SY5Y (BODIPY/DAPI)
C1 (5 μM)
C2 (5 μM)
Neg_si
ATG5_si
Neg_siRNA
ATG5_siRNA
averaged LD# / cell
p= 0.43
p= 0.15
p= 0.0011
p= 0.008
p= 0.006
p= 0.07
p= 0.0001
c
SH-SY5Y cells
p=0.061
normalized cell-titer glo (%)
no DMSO
50 μM C1
50 μM C2
50 μM C3
50 μM C4
d
WT MEFs
OA induced
TAG (mM) per μg total protein
p= 0.97
p= 0.99
p= 0.60
BSA
GW
DP
SIII
Atg5-/- MEFs
p= 0.96
p= 0.12
p= 0.95
p= 0.97

Fig. S3 The LD·ATTECs C3 and C4 reduced the OA-induced LDs in an autophagy dependent manner.

a Representative images and quantifications of OA-induced LDs in wild-type or Atg5$^{-/-}$ MEFs treated with indicated compounds ($n = 9$). **b** Similar to a , but in SH-SY5Y cells transfected with the non-targeting control (Neg_si) or *Atg5* targeting (Atg5_si) siRNA. **c** Cell viability measurements of compound-treated SH-SY5Y cells by cell-titer glo. Treatment of LD·ATTECs at a very high concentration (50 μM) led to no observable cytotoxicity after 3 days. **d** Measurements of cellular TAG concentrations in the WT or Atg5$^{-/-}$ MEFs. For all plots, bars indicate mean and s.e.m.. The statistical analyses were performed by one-way ANOVA and Dunnett's post-hoc tests (the corrected p values compared to the DMSO group, if the ANOVA tests were significant). The n numbers indicate independently plated wells.
