# Supplementary material for: Degradation of lipid droplets by chimeric autophagy-tethering compounds
Source: Cell Res. 2021 Jul 8;31(9):965–79. doi: 10.1038/s41422-021-00532-7 (PMC8410765; doi:10.1038/s41422-021-00532-7)
Supplement: Supplementary file 4 — Supplementary information, Fig. S4 [file 41422_2021_532_MOESM4_ESM.pdf]

Fig. S4

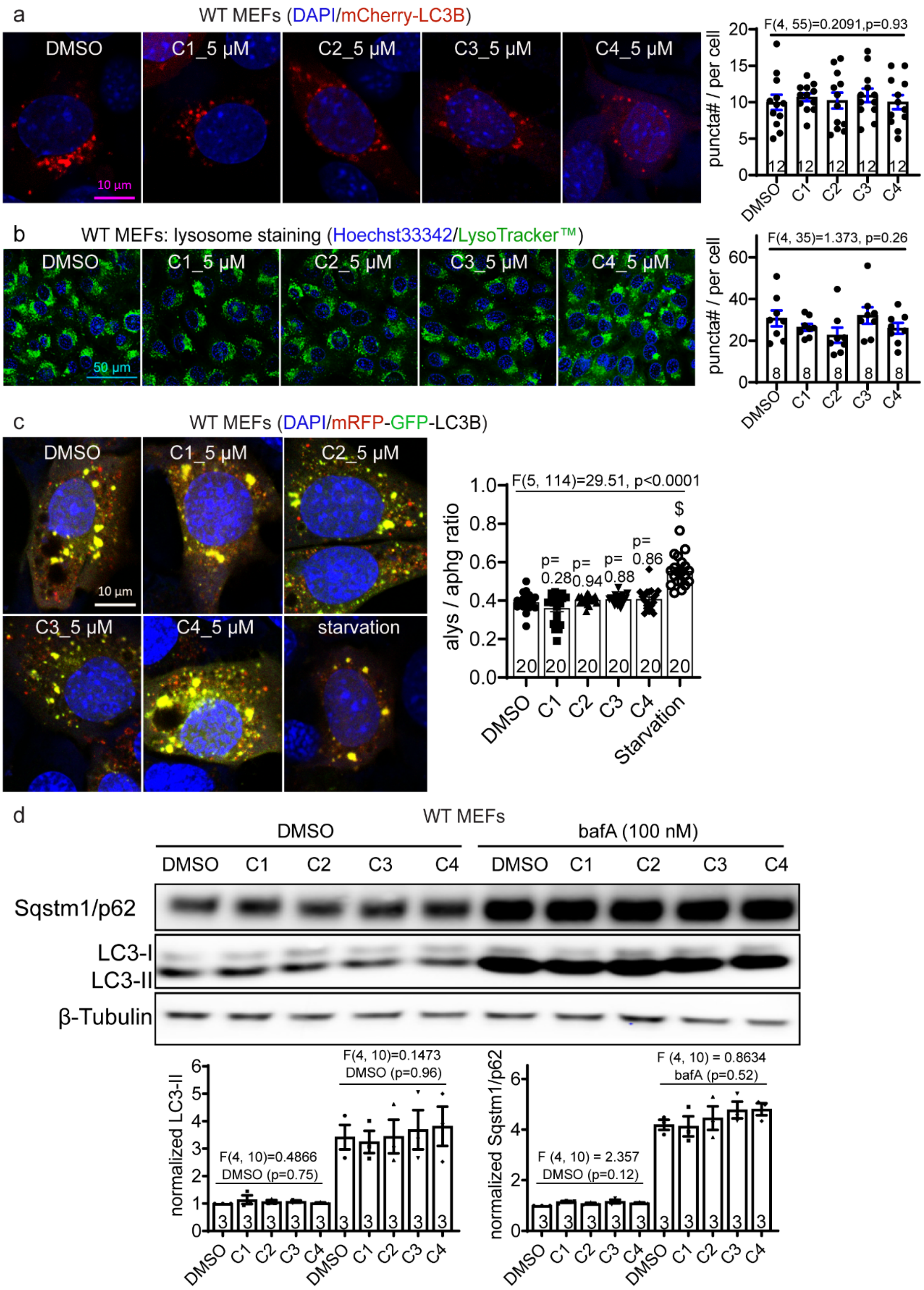
a
WT MEFs (DAPI/mCherry-LC3B)
DMSO
C1_5 μM
C2_5 μM
C3_5 μM
C4_5 μM
10 μm
F(4, 55)=0.2091,p=0.93
puncta# / per cell
DMSO
C1
C2
C3
C4
b
WT MEFs: lysosome staining (Hoechst33342/LysoTracker™)
50 μm
F(4, 35)=1.373, p=0.26
c
WT MEFs (DAPI/mRFP-GFP-LC3B)
starvation
F(5, 114)=29.51, p<0.0001
alys / aphg ratio
p= 0.28
p= 0.94
p= 0.88
p= 0.86
$
Starvation
d
WT MEFs
bafA (100 nM)
Sqstm1/p62
LC3-I
LC3-II
β-Tubulin
normalized LC3-II
F(4, 10)=0.4866
DMSO (p=0.75)
F(4, 10)=0.1473
DMSO (p=0.96)
normalized Sqstm1/p62
F (4, 10) = 2.357
DMSO (p=0.12)
F (4, 10) = 0.8634
bafA (p=0.52)

Fig. S4 LD·ATTECs did not influence cellular autophagy activity. a Representative images and quantifications of mCherry-LC3B transfected MEFs treated with the indicated compounds. Treatment of LD·ATTECs (C1, C2, C3 or C4) did not change the LC3B puncta number, suggesting no influence on autophagosomes. **b** Representative images and quantifications of lysosomes using lysotracker. Treatment of LD·ATTECs (C1, C2, C3 or C4) did not change the lysotracker puncta number, suggesting no influence on lysosomes. **c** Representative images and quantifications of mRFP-GFP-LC3B transfected MEFs treated with the indicated compounds or starvation (medium replacement with EBSS for 4 hours). Red only puncta indicate autolysosomes and yellow puncta indicate autophagosomes. Treatment of LD·ATTECs (C1, C2, C3 or C4) did not alter the autolysosome (alys; red+, green- puncta) / autophagosome (aphg; red+, green+) ratio, suggesting no influence on autophagy flux. **d** Representative images and quantifications of LC3B and SQSTM1/p62 western-blots of MEFs treated with the indicated compounds. No significant effects by treatment of LD·ATTECs (C1, C2, C3 or C4 at 5 μM) were observed in the presence or absence of bafA (bafilomycin A1). Bars indicate mean and s.e.m.. The statistical analyses were performed by one-way ANOVA (F/degree of freedom/p values have been indicated for each plot) and Dunnett's post-hoc tests (compared to the DMSO group; corrected p values on top of each bar; "$": $p<0.0001$).
