# Supplementary material for: Degradation of lipid droplets by chimeric autophagy-tethering compounds
Source: Cell Res. 2021 Jul 8;31(9):965–79. doi: 10.1038/s41422-021-00532-7 (PMC8410765; doi:10.1038/s41422-021-00532-7)
Supplement: Supplementary file 5 — Supplementary information, Fig. S5 [file 41422_2021_532_MOESM5_ESM.pdf]

Fig. S5

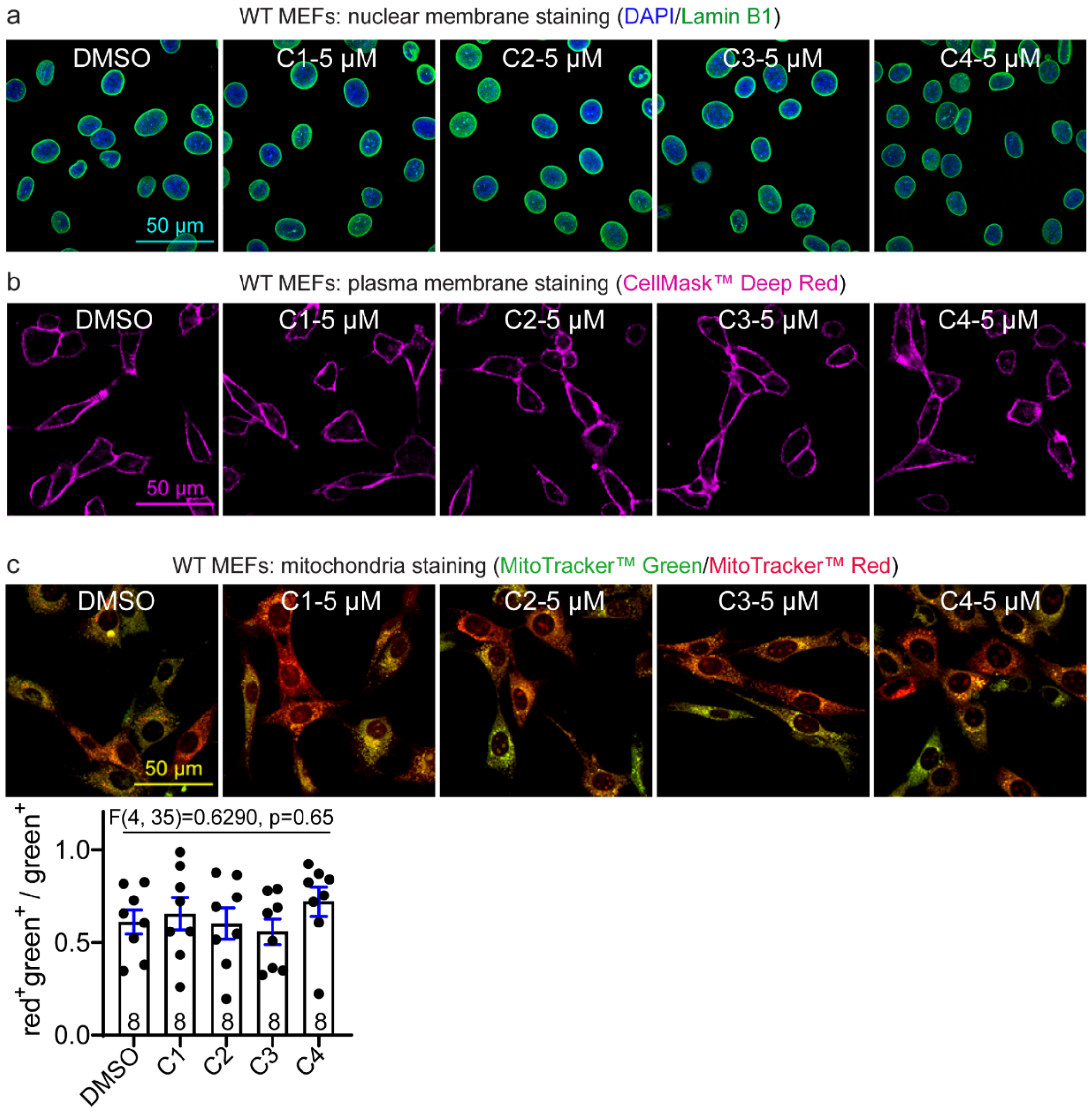

Fig. S5 LD·ATTECs did not influence the integrity of plasma or intracellular membranes.

a-b Representative staining images (from >3 biological replicates) showing that LD·ATTECs (C1, C2, C3 or C4) did not damage nuclear membranes (**a**, Lamin B1 immunostaining) or plasma membranes (**b**, CellMask^{TM} staining). **c** Representative images of quantifications of the healthy mitochondria (MitoTracker^{TM} Red) : total mitochondria (MitoTracker^{TM} Green) ratio in each well. The total red^{+}green^{+} area / green^{+} area ratio of each well indicates the ratio of healthy mitochondria, representing the integrity of mitochondria membranes. Bars indicate mean and s.e.m.. The statistical analyses were performed by one-way ANOVA (F/degree of freedom/p values have been indicated). The n numbers indicates the number of independently treated wells.
