# Supplementary material for: Degradation of lipid droplets by chimeric autophagy-tethering compounds
Source: Cell Res. 2021 Jul 8;31(9):965–79. doi: 10.1038/s41422-021-00532-7 (PMC8410765; doi:10.1038/s41422-021-00532-7)
Supplement: Supplementary file 6 — Supplementary information, Fig. S6 [file 41422_2021_532_MOESM6_ESM.pdf]

Fig. S6

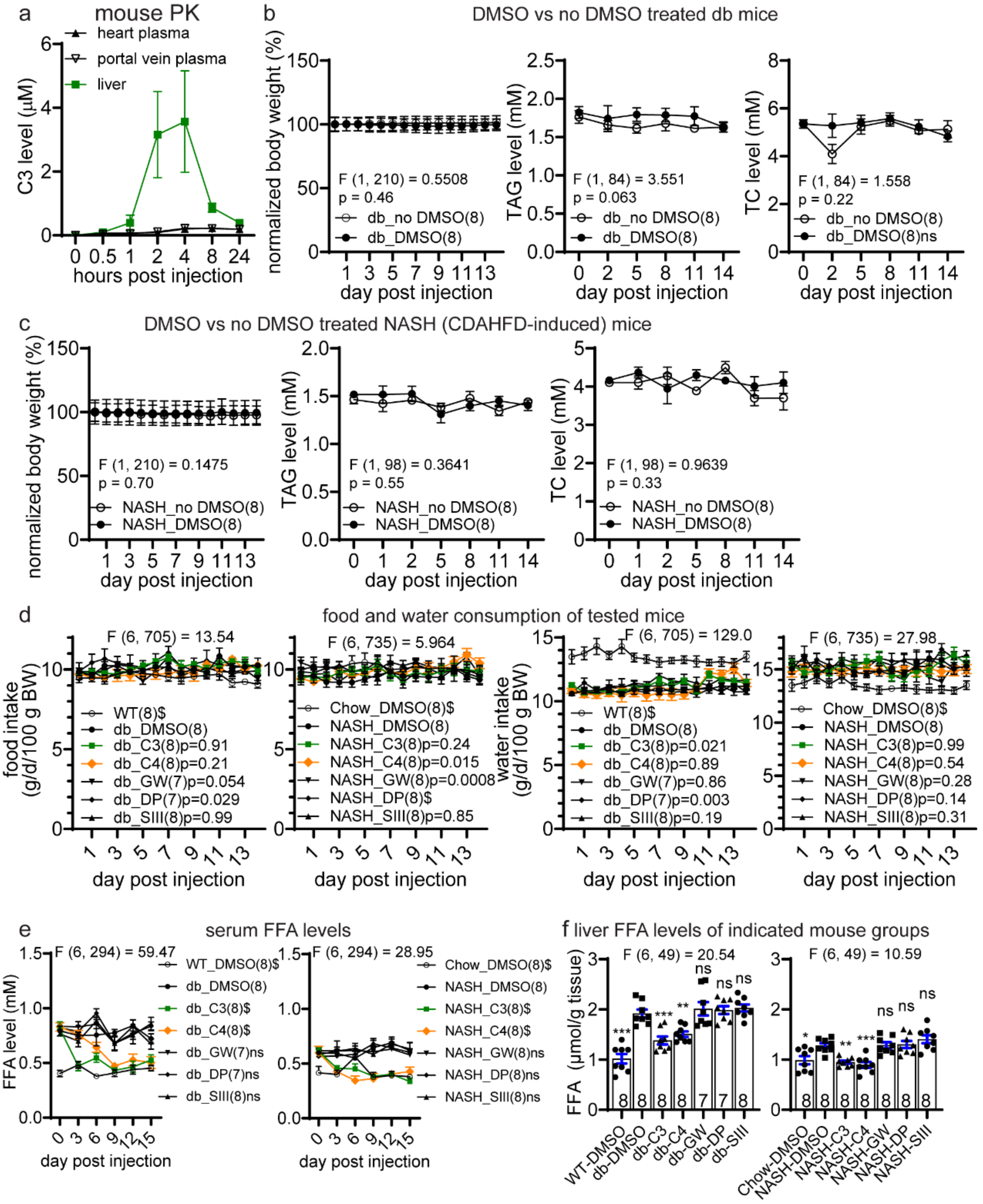
a
mouse PK
heart plasma
portal vein plasma
liver
C3 level (μM)
hours post injection
b
DMSO vs no DMSO treated db mice
normalized body weight (%)
F (1, 210) = 0.5508
p = 0.46
db_no DMSO(8)
db_DMSO(8)
day post injection
TAG level (mM)
F (1, 84) = 3.551
p = 0.063
db_no DMSO(8)
db_DMSO(8)
day post injection
TC level (mM)
F (1, 84) = 1.558
p = 0.22
db_no DMSO(8)
db_DMSO(8)ns
day post injection
c
DMSO vs no DMSO treated NASH (CDAHFD-induced) mice
normalized body weight (%)
F (1, 210) = 0.1475
p = 0.70
NASH_no DMSO(8)
NASH_DMSO(8)
day post injection
TAG level (mM)
F (1, 98) = 0.3641
p = 0.55
NASH_no DMSO(8)
NASH_DMSO(8)
day post injection
TC level (mM)
F (1, 98) = 0.9639
p = 0.33
NASH_no DMSO(8)
NASH_DMSO(8)
day post injection
d
food and water consumption of tested mice
food intake (g/d/100 g BW)
F (6, 705) = 13.54
WT(8)$
db_DMSO(8)
db_C3(8)p=0.91
db_C4(8)p=0.21
db_GW(7)p=0.054
db_DP(7)p=0.029
db_SIII(8)p=0.99
day post injection
F (6, 735) = 5.964
Chow_DMSO(8)$
NASH_DMSO(8)
NASH_C3(8)p=0.24
NASH_C4(8)p=0.015
NASH_GW(8)p=0.0008
NASH_DP(8)$
NASH_SIII(8)p=0.85
day post injection
water intake (g/d/100 g BW)
F (6, 705) = 129.0
WT(8)$
db_DMSO(8)
db_C3(8)p=0.021
db_C4(8)p=0.89
db_GW(7)p=0.86
db_DP(7)p=0.003
db_SIII(8)p=0.19
day post injection
F (6, 735) = 27.98
Chow_DMSO(8)$
NASH_DMSO(8)
NASH_C3(8)p=0.99
NASH_C4(8)p=0.54
NASH_GW(8)p=0.28
NASH_DP(8)p=0.14
NASH_SIII(8)p=0.31
day post injection
e
serum FFA levels
FFA level (mM)
F (6, 294) = 59.47
WT_DMSO(8)$
db_DMSO(8)
db_C3(8)$
db_C4(8)$
db_GW(7)ns
db_DP(7)ns
db_SIII(8)ns
day post injection
F (6, 294) = 28.95
Chow_DMSO(8)$
NASH_DMSO(8)
NASH_C3(8)$
NASH_C4(8)$
NASH_GW(8)ns
NASH_DP(8)ns
NASH_SIII(8)ns
day post injection
f liver FFA levels of indicated mouse groups
FFA (μmol/g tissue)
F (6, 49) = 20.54
WT-DMSO
db-DMSO
db-C3
db-C4
db-GW
db-DP
db-SIII
F (6, 49) = 10.59
Chow-DMSO
NASH-DMSO
NASH-C3
NASH-C4
NASH-GW
NASH-DP
NASH-SIII

Fig. S6 Control experiments of *in vivo* testing. a Measurements of C3 concentrations in liver and indicated plasma samples collected at the indicated time points after IP injection (30 mg/kg). A trial test with 10 mg/kg was performed but failed to reach desired concentrations (not shown). **b** Body weight, serum TC and TAG levels of DMSO-containing vehicle injected versus non DMSO-containing vehicle injected db/db mice. DMSO did not lower any of these parameters, but caused an insignificant increase of the serum TAG level instead. Note that the DMSO group presented here is the same group used in Fig. 5. **c** Similar to (**b**), but in NASH mice. Note that the DMSO group presented here is the same group used in Fig. 7. **d** Body-weight normalized food and water intake of indicated mouse groups, which are the same groups presented in Fig. 5-6. The WT_DMSO group is the same as Chow_DMSO group. Note that in db mice, C4 and C3 led to a marginally significant increase rather than decrease in body weight-normalized food and water intake, respectively. **e-f** Serum (**e**) and endpoint liver (**f**) free fatty acid (FFA) levels of indicated groups, which are the same groups presented in Fig. 5-6. For all plots, data were plotted as mean and s.e.m., except for the normalized body weight, which was plotted as mean and s.d. to exhibit the error bars. ns: $p>0.05$, "$": $p<0.0001$; exact p values are shown if space allows. For measurements at multiple times points, two-way ANOVA and Dunnett's post-hoc analysis (compared to the DMSO treated db or NASH group) was performed. For endpoint measurements of multiple groups, one-way ANOVA and Dunnett's post-hoc analysis (compared to the DMSO treated db or NASH group) was performed.
