# Supplementary material for: Degradation of lipid droplets by chimeric autophagy-tethering compounds
Source: Cell Res. 2021 Jul 8;31(9):965–79. doi: 10.1038/s41422-021-00532-7 (PMC8410765; doi:10.1038/s41422-021-00532-7)
Supplement: Supplementary file 7 — Supplementary information, Fig. S7 [file 41422_2021_532_MOESM7_ESM.pdf]

Fig. S7

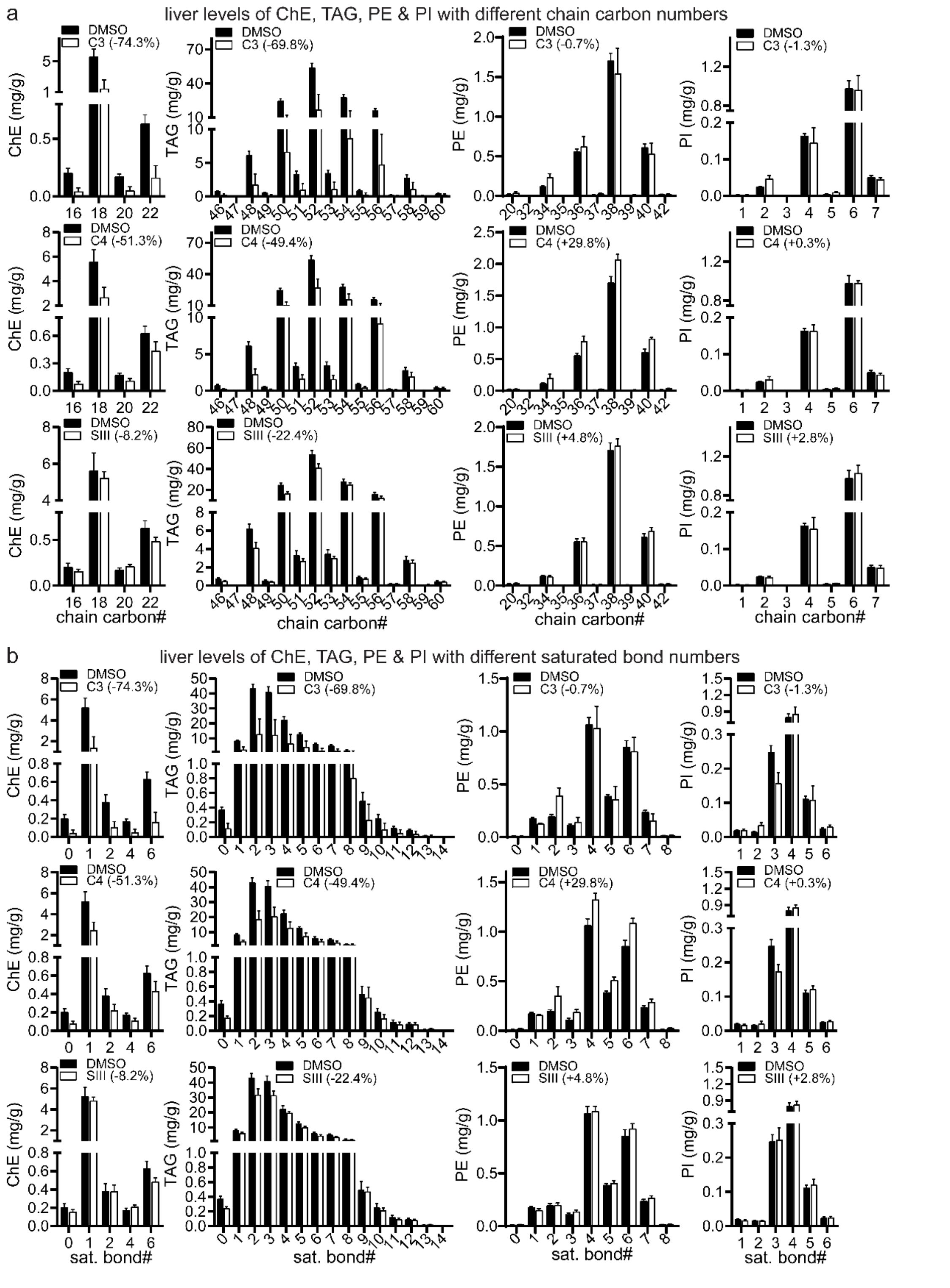

Fig. S7 Analysis of exemplar lipids of compound injected liver samples based on the lipidomics data. a levels of ChE, TAG, PE or PI with different fatty acid chain carbon numbers (chain carbon#). The numbers in the brackets indicate the overall change of the treated sample compared to the DMSO control. **b** Similar to **a**, but with different saturated bonds (sat. bond#).
