# Supplementary material for: Degradation of lipid droplets by chimeric autophagy-tethering compounds
Source: Cell Res. 2021 Jul 8;31(9):965–79. doi: 10.1038/s41422-021-00532-7 (PMC8410765; doi:10.1038/s41422-021-00532-7)
Supplement: Supplementary file 8 — Supplementary information, Fig. S8 [file 41422_2021_532_MOESM8_ESM.pdf]

## Fig. S8

a

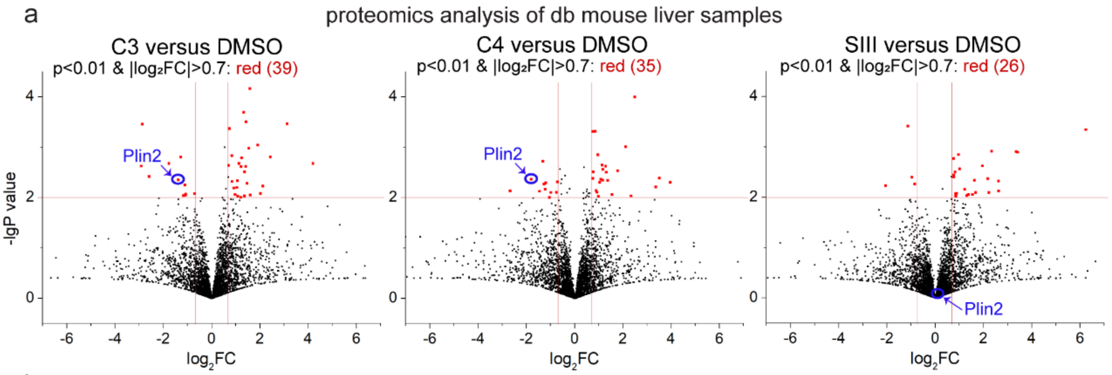


b

lists of changed proteins

| C3 versus DMSO | | | |
|---|---|---|---|
| Down proteins | | Up proteins | |
| GeneID | Symbol | GeneID | Symbol |
| 27404 | Abca8b | 14972 | H2-K1 |
| 67996 | Srsf6 | 56709 | Dnajb12 |
| 67441 | Isoc2b | 12492 | Scarb2 |
| 103172 | Chchd10 | 80285 | Parp9 |
| 11520 | Plin2 | 11843 | Arf4 |
| 66302 | Rmdn1 | 231510 | Agpat9 |
| 30055 | Timm13 | 14964 | H2-D1 |
| 18194 | Nsdhl | 13849 | Ephx1 |
| 14268 | Fn1 | 14862 | Gstm1 |
| 211389 | Suox | 14864 | Gstm3 |
| 27425 | Atp5l | 14870 | Gstp1 |
| | | 20846 | Stat1 |
| | | 59027 | Nampt |
| | | 68312 | Gstm7 |
| | | 20288 | Msr1 |
| | | 16852 | Lgals1 |
| | | 21356 | Tapbp |
| | | 14863 | Gstm2 |
| | | 26444 | Psma7 |
| | | 19192 | Psme3 |
| | | 14867 | Gstm6 |
| | | 28028 | Mrpl50 |
| | | 76408 | Abcc3 |
| | | 68631 | Cryl1 |
| | | 17069 | Ly6e |
| | | 17476 | Mpeg1 |
| | | 69550 | Bst2 |
| | | 15957 | Ifit1 |

| C4 versus DMSO | | | |
|---|---|---|---|
| Down proteins | | Up proteins | |
| GeneID | Symbol | GeneID | Symbol |
| 22596 | Xrcc5 | 229211 | Acad9 |
| 11520 | Plin2 | 14864 | Gstm3 |
| 20740 | Sptan1 | 15894 | Icam1 |
| 101488 | Slco2b1 | 16922 | Phyh |
| 27407 | Abcf2 | 29875 | Iqgap1 |
| 226105 | Cyp2c70 | 14867 | Gstm6 |
| 66302 | Rmdn1 | 21356 | Tapbp |
| 211389 | Suox | 19063 | Ppt1 |
| 11637 | Ak2 | 72479 | Hsdl2 |
| 14711 | Gnmt | 18631 | Pex11a |
| 110391 | Qdpr | 56212 | Rhog |
| | | 320024 | Nceh1 |
| | | 14225 | Fkbp1a |
| | | 14148 | Fdx1 |
| | | 16852 | Lgals1 |
| | | 14870 | Gstp1 |
| | | 11886 | Asah1 |
| | | 28028 | Mrpl50 |
| | | 69550 | Bst2 |
| | | 66234 | Sc4mol |
| | | 66845 | Mrpl33 |
| | | 70266 | Ccbl1 |
| | | 22099 | Tsn |
| | | 75552 | Paqr9 |

**Fig. S8 Proteomic analyses of compound injected liver samples. a** Volcano plots of the proteomic analyses of liver samples from mice injected with C3, C4 or SIII versus the DMSO control for 14 days (4 mice per group). Only the proteins detected in at least one sample in each group of the comparison were plotted to avoid error in calculating the $\log_2$ fold changes ($\log_2$FC) and or the $-\log_{10}$P values (-lgP values). The LD marker protein Plin2 was indicated by the blue circle and arrow in each plot. The statistical analysis was performed for two-tailed unpaired t test. **b** The tables and Venn diagram illustrating significantly changed proteins for each comparison ($|\log_2FC| > 0.8$ and $p < 0.01$). See Data S2 for detailed data used for the analysis and plots.
