# Supplementary material for: Degradation of lipid droplets by chimeric autophagy-tethering compounds
Source: Cell Res. 2021 Jul 8;31(9):965–79. doi: 10.1038/s41422-021-00532-7 (PMC8410765; doi:10.1038/s41422-021-00532-7)
Supplement: Supplementary file 9 — Supplementary information, Fig. S9 [file 41422_2021_532_MOESM9_ESM.pdf]

Fig. S9

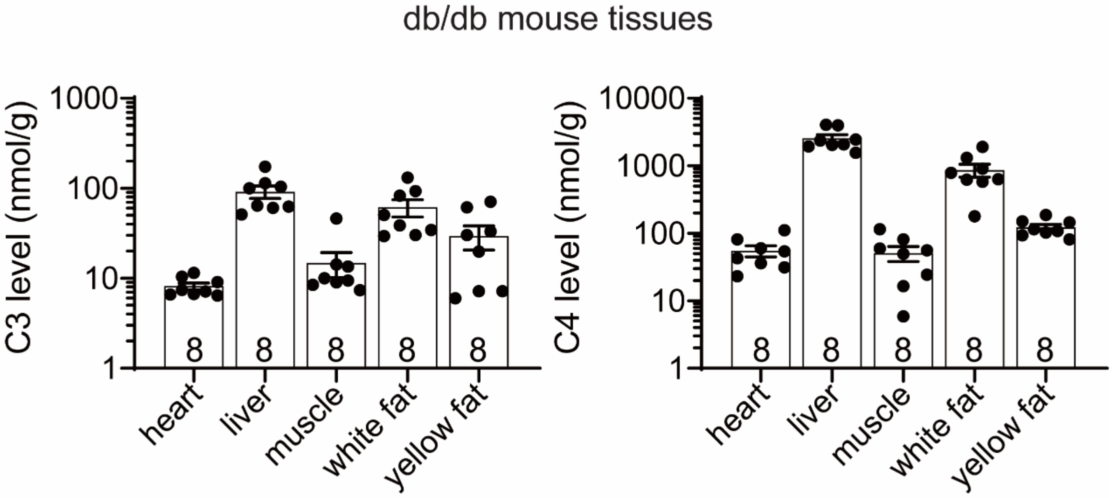

Fig S9 Compounds' distribution in db/db mouse tissue samples. Bar plots (mean and s.e.m.) of C3 or C4 concentrations in the indicated tissues from db/db mice injected with these compounds for 14 days. Significant enrichment in the liver and white fat tissues were observed.
