# Supplementary material for: Degradation of lipid droplets by chimeric autophagy-tethering compounds
Source: Cell Res. 2021 Jul 8;31(9):965–79. doi: 10.1038/s41422-021-00532-7 (PMC8410765; doi:10.1038/s41422-021-00532-7)
Supplement: Supplementary file 10 — Supplementary information, Fig. S10 [file 41422_2021_532_MOESM10_ESM.pdf]

Fig. S10

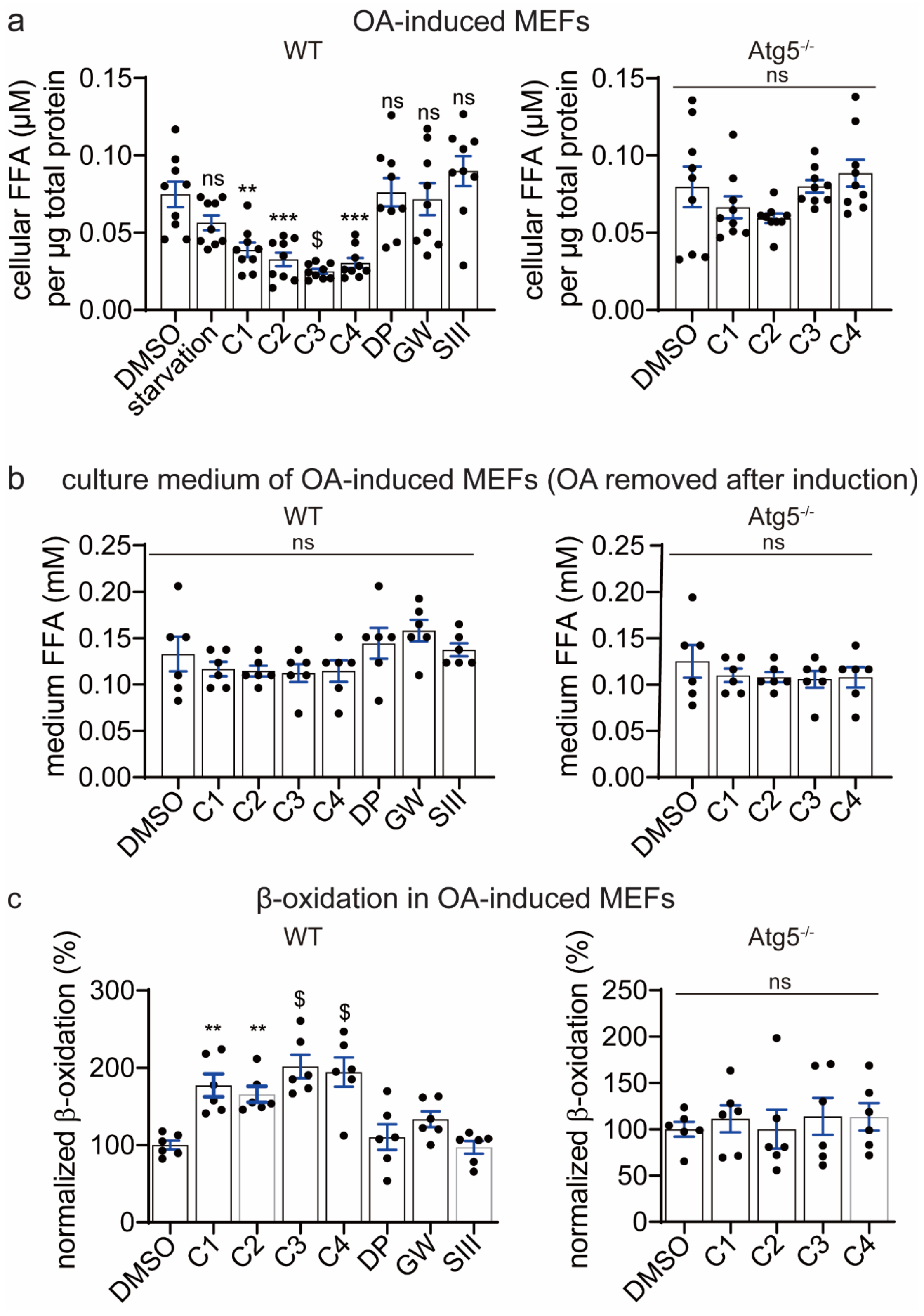
a
OA-induced MEFs
WT
Atg5-/-
cellular FFA (μM) per μg total protein
0.15
0.10
0.05
0.00
ns
**
***
$
***
ns
ns
ns
DMSO
starvation
C1
C2
C3
C4
DP
GW
SIII
ns
b
culture medium of OA-induced MEFs (OA removed after induction)
WT
Atg5-/-
medium FFA (mM)
0.25
0.20
0.15
0.10
0.05
0.00
ns
ns
c
β-oxidation in OA-induced MEFs
WT
Atg5-/-
normalized β-oxidation (%)
300
200
100
0
**
**
$
$
250
200
150
100
50
0
ns

Fig. S10 Treatment of LD·ATTECs lowered FFA levels and increased β-oxidation in the cells, but not FFA levels in the culture medium. Bar plots (mean and s.e.m.) of cellular FFA **a**, medium FFA **b**, and β-oxidation, **c** of the cells. For medium FFA measurements, medium replacement was performed to eliminate potential influence of OA for the measurement. One-way ANOVA and Dunnett's post-hoc analysis (compared to the DMSO treated db or NASH group if the ANOVA test showed significance) was performed. ns: $p<0.05$, *: $p<0.05$, **: $p<0.01$, ***: $p<0.001$, ***: $p<0.0001$. Note that some of the results did not show significance in the ANOVA tests (indicated by "ns" above the horizontal lines).
