# Supplementary material for: Degradation of lipid droplets by chimeric autophagy-tethering compounds
Source: Cell Res. 2021 Jul 8;31(9):965–79. doi: 10.1038/s41422-021-00532-7 (PMC8410765; doi:10.1038/s41422-021-00532-7)
Supplement: Supplementary file 11 — Supplementary information, Data S1 [file 41422_2021_532_MOESM11_ESM.pdf]

Supplementary information, Data S1

Synthesis and characterization of LD·ATTECs

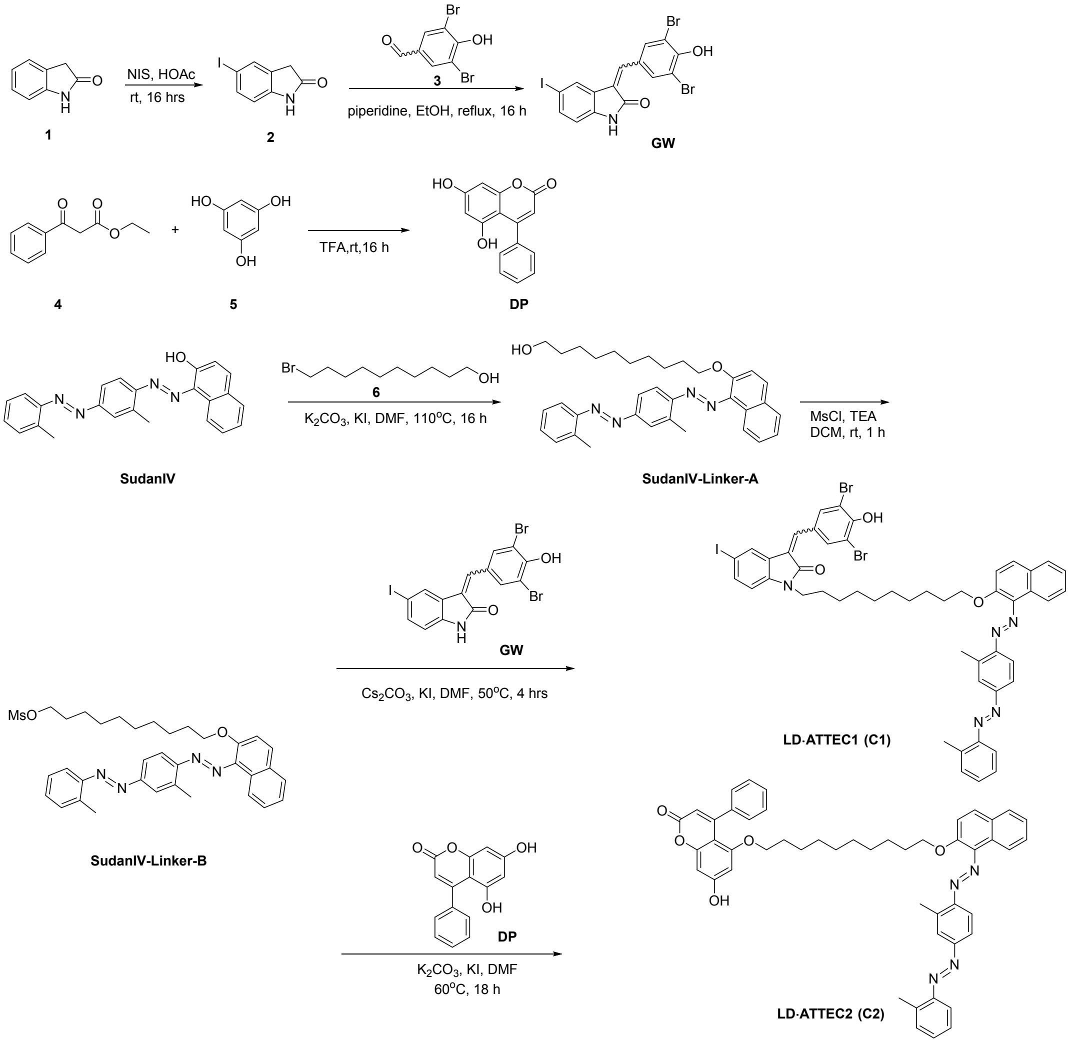

1. Synthesis of **LD·ATTEC1 (C1)**: 3-(3,5-dibromo-4-hydroxybenzylidene)-5-iodo-1-(10-((1-((E)-(4-((E)-phenyldiazenyl)phenyl)diazenyl)naphthalen-2-yl)oxy)decyl)indolin-2-one

Step 1: Preparation of intermediate **2**, 5-iodoindolin-2-one, MS m/z (ESI): 259.9 [M+H]$^+$.

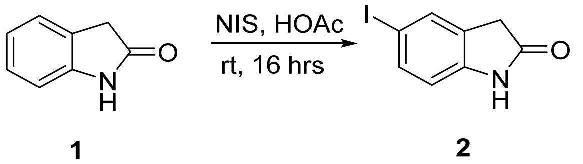

A solution of indoline-2-one (2.0 g, 15.1 mmol) and NIS (4.1 g, 18.1 mmol) in acetic acid (25 mL) was stirred at room temperature for 16 hours, and then the reaction solution was concentrated using a rotary evaporator to obtain the product.

The product was further slurred with ethyl acetate, filtered, and the filter cake was rinsed with a small amount of ethyl acetate and dried to obtain the product as a red solid 5-iodoindolin-2-one (2.8 g, yield: 71.9%).

Step 2: Preparation of **GW**, (3-(3,5-dibromo-4-hydroxybenzylidene)-5-iodoindolin-2-one), MS m/z (ESI): 521.7 [M+H]$^+$.

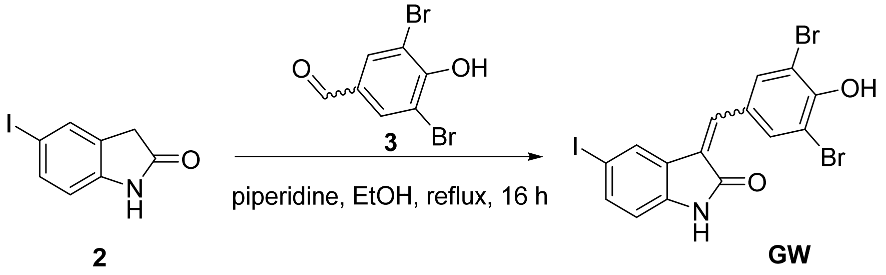

Dissolve 5-iodoindolin-2-one (2.8 g, 10.81 mmol) in ethanol (30 mL), add 3,5-dibromo-4-hydroxybenzaldehyde (3.01 g, 10.81 mmol) and piperidine (183 mg, 1.54 mmol). The mixture was reacted at 80 °C for 16 hours. The reaction solution was cooled to room temperature, filtered, and the filter cake was rinsed with diethyl ether and petroleum ether, and dried to obtain GW as a yellow solid (2.3 g, 40.8%).

Step 3: Preparation of **SudanIV-Linker-A**, 10-((1-((E)-(2-methyl-4-((E)-o-tolyldiazenyl)phenyl)diazenyl)naphthalen-2-yl)oxy)decan-1-ol, MS m/z (ESI): 537.1 [M+H]$^+$.

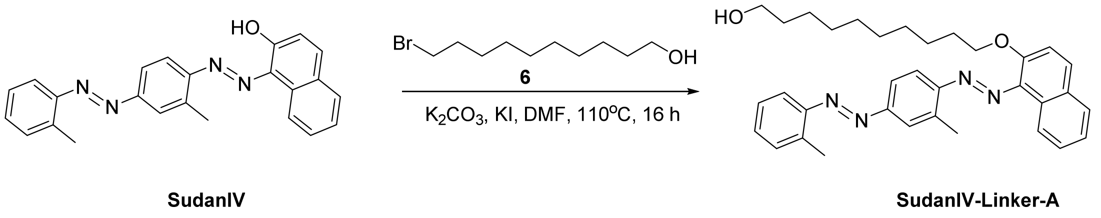

The reactants Sudan Red IV (4 g, 10.52 mmol), Compound 6 (10-bromo-1-decanol) (2.72 g, 11.56 mmol), potassium carbonate (8.72 g, 6 3.6mmol) and potassium iodide (175 mg, 1.052 mmol) were added to 250 mL DMF (30 mL) in the flask and stirred at 110 °C for 168 hours. The reaction was cooled to room temperature, water (200 mL) was added, and extracted with ethyl acetate (2 × 100 mL). The organic layers were combined and spin-dried, and the obtained product was purified by column chromatography (petroleum ether/ethyl acetate=5/1) to obtain a red oily product (2400 mg, yield: 43%).

Step 4: Preparation of **SudanIV-Linker-B**, 10-((1-((E)-(2-methyl-4-((E)-o-tolyldiazenyl)phenyl)diazenyl)naphthalen-2-yl)oxy)decyl methanesulfonate, MS m/z (ESI): 615.1 [M+H]$^+$.

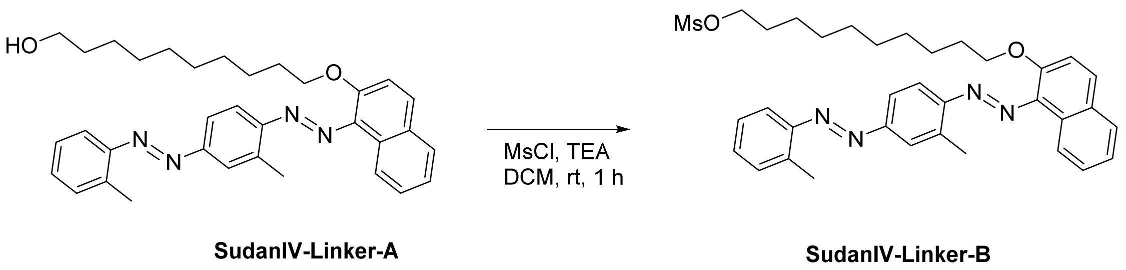

The reactant SudanIV-Linker-A (2400 mg, 4.48 mmol) and triethylamine (904 mg, 8.96 mmol) were dissolved in methylene chloride (20 mL), and methanesulfonyl chloride (616 mg, 5.38 mmol) was added dropwise. The mixture was stirred at room temperature for 1 hour. 100 mL of water was added to the reaction solution, and extracted with dichloromethane (2 × 100 mL). The organic layer was spin-dried, and the resulting product was purified by column chromatography (petroleum ether/ethyl acetate = 3/1) to obtain a red oily product (2200 mg, yield: 80%).

Step 5: Preparation of **LD·ATTEC1 (C1)**, 3-(3,5-dibromo-4-hydroxybenzylidene)-5-iodo-1-(10-((1-((E)-(2-methyl-4-((E)-o-tolyldiazenyl)phenyl)diazenyl)naphthalen-2-yl)oxy)decyl)indolin-2-one, MS m/z (ESI): 1040.1 $[M+H]^+$.

^{1}H NMR (400 MHz, $CDCl_3$): δ 8.59-8.61 (d, 2H), 8.52 (d, *J*=8.4 Hz, 1H), 7.71-7.94 (m, 7H), 7.67 (d, *J*=7.6 Hz, 1H), 7.19-7.61 (m, 9H), 6.53-6.57 (m, 1H), 4.18-4.22 (m, 1H), 3.65-3.69 (m, 2H), 2.86 (d, 3H), 2.86 (d, 3H), 1.24-1.27 (m, 6H).1.24-1.27 (m, 10H).

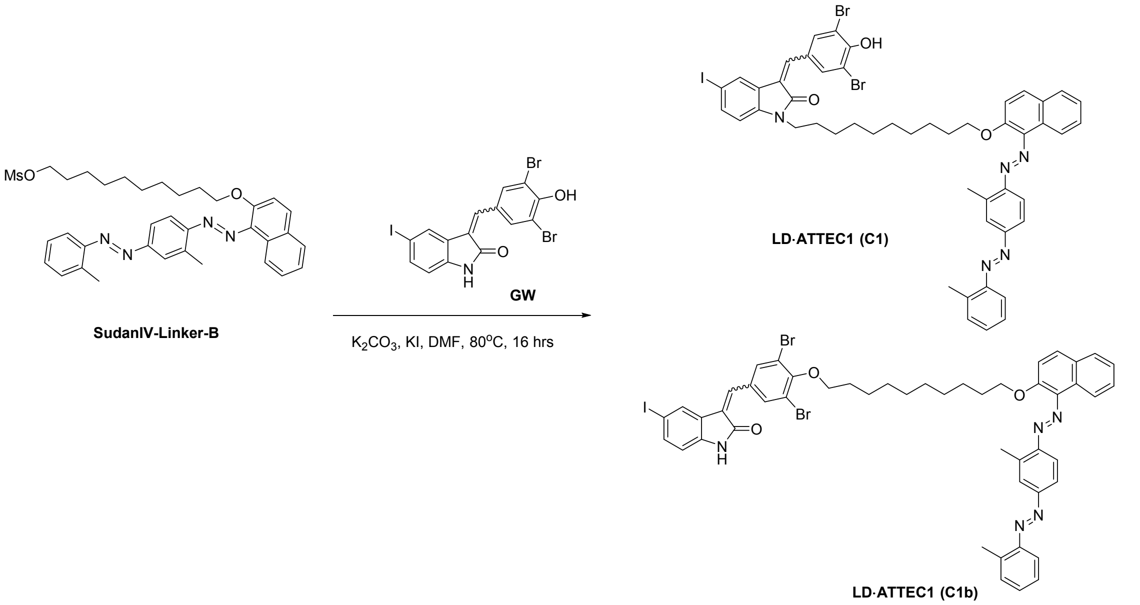

Intermediate GW (521 mg, 1.0 mmol), SudanIV-Linker-B (614 mg, 1.0 mmol), caesium carbonate (3260 mg, 10.0 mmol) and potassium iodide (166 mg, 1.0 mmol) in DMF (20 mL) were mixed and reacted at 50 °C for 4 hours. The reaction was

cooled to room temperature, quenched with water (20 mL), extracted with ethyl acetate (50 mL × 3), the organic phase was separated, and the organic phase was washed with water (20 mL × 3) and saturated sodium chloride solution (50 mL × 1), dry over anhydrous sodium sulfate, filter, and the solvent was removed under reduced pressure. The resulting residue was prepared by column chromatography (petroleum ether: ethyl acetate = 3:1) and reversed-phase (100% acetonitrile) to obtain a red solid (218 mg, 21%). There was also another low yield by-product with same molecular weight link to -OH not -NH, LD·ATTEC1b (C1b, 3-(3,5-dibromo-4-((10-((1-((E)-(2-methyl-4-((E)-o-tolyldiazenyl)phenyl)diazenyl)naphthalen-2-yl)oxy)decyl)oxy)benzylidene)-5-iodoindolin-2-one) synthesized and purified, with LC3B-binding capability as well (K_d = 21.4 ± 1.8 μM measured by MST; note that it has very poor solubility and the affinity could have been underestimated).

2. Synthesis of **LD·ATTEC2 (C2)**: LD·ATTEC-2: 7-hydroxy-5-((10-((1-((E)-(2-methyl-4-((E)-o-tolyldiazenyl)phenyl)diazenyl)naphthalen-2-yl)oxy)decyl)oxy)-4-phenyl-2H-chromen-2-one

Step 1: Preparation of intermediate **DP**, 5,7-dihydroxy-4-phenyl-2H-chromen-2-one, MS m/z (ESI): 255.1 $[M+H]^+$.

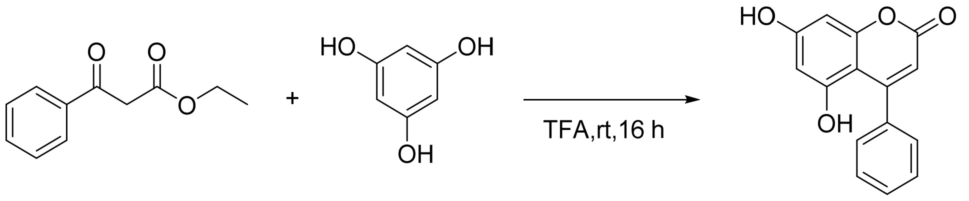

4 **5** **DP**

Add ethyl 3-oxo-3-phenylpropionate (3 g, 15.63 mmol) and 1,3,5-trihydroxybenzene (1.97 g, 15.63 mmol) to the reaction flask, add 20 mL of trifluoro acetic acid, stirred at room temperature for 16 hours. After the reaction was completed, water (150 mL) was added, the product was filtered. The obtained solid was purified by reverse phase chromatography to obtain a yellow solid (2 g, yield: 50.4%).

Step 2: Preparation of **LD·ATTEC2 (C2)**, 7-hydroxy-5-((10-((1-((E)-(2-methyl-4-((E)-o-tolyldiazenyl)phenyl)diazenyl)naphthalen-2-yl)oxy)decyl)oxy)-4-phenyl-2H-chromen-2-one, MS m/z (ESI): 773.3 $[M+H]^+$. ^1H NMR (400 MHz, $CDCl_3$) δ 8.60 (d, J = 8.4 Hz, 1H), 7.82-7.93 (m, 5H), 7.65-7.66 (d, J = 7.6 Hz, 1H), 7.52-7.58(m, 1H), 7.34-7.46 (m, 4H), 7.27-7.32 (m, 4H), 7.20-7.22 (m, 2H), 6.61-6.63 (m, 2H), 6.12 (d, J = 2.4 Hz, 1H), 5.93 (s, 1H), 4.22 (t, J = 6.4 Hz, 2H), 3.54 (t, J = 6.4 Hz, 2H), 2.86 (s, 3H), 2.76 (s, 3H), 1.82-1.86 (m, 2H), 1.45-1.49 (m, 2H), 1.27-1.33 (m, 2H), 1.18-1.25 (m, 2H), 1.08-1.12 (m, 2H), 0.98-1.03 (m, 4H), 0.74-0.78 (m, 2H).

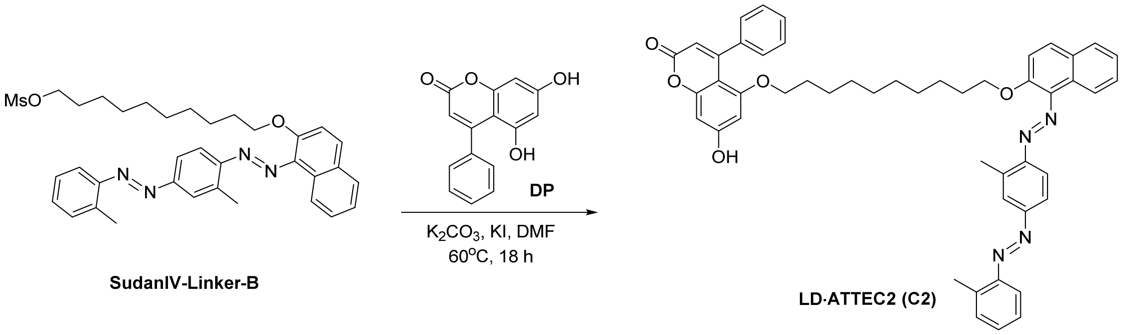

Add SudanIV-Linker-B (1200 mg, 1.95 mmol), DP (992 mg, 3.9 mmoL), potassium carbonate (1618 mg, 11.7 mmol) and potassium iodide (166 mg, 1 mmol) to a 100 mL flask and add DMF (15 mL), stirred at 60°C for 18 hours. The reaction was cooled to room temperature, water (60 mL) was added, and extracted with ethyl acetate (2x30 mL). The organic layer was combined and spin-dried, and the resulting product was purified by reverse phase chromatography (petroleum ether: ethyl acetate = 2:1~1:1) to obtain a red solid product (205 mg, yield: 13.6%).

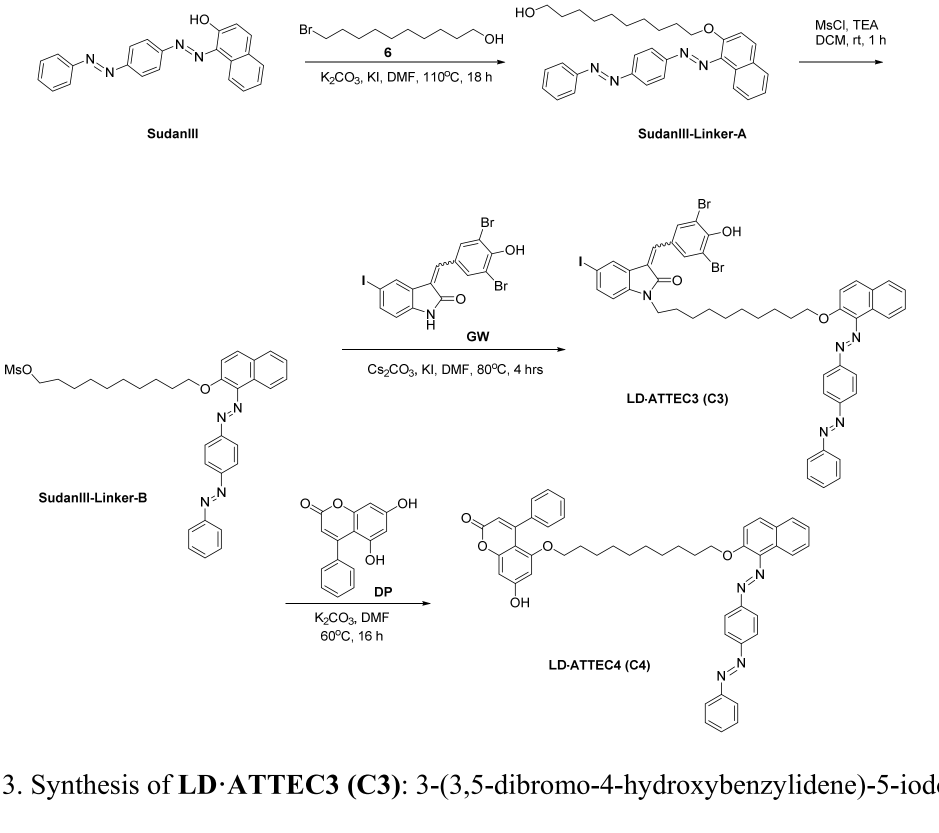

3. Synthesis of **LD·ATTEC3 (C3)**: 3-(3,5-dibromo-4-hydroxybenzylidene)-5-iodo-1-(10-((1-((E)-(4-((E)-phenyldiazenyl)phenyl)diazenyl)naphthalen-2-yl)oxy)decyl)indolin-2-one

Step 1: Preparation of the intermediate **SudanIII-Linker-A**, 10-((1-((E)-(4-((E)-phenyldiazenyl)phenyl)diazenyl)naphthalen-2-yl)oxy)decan-1-ol, MS m/z (ESI): 509.0 [M+H]$^+$.

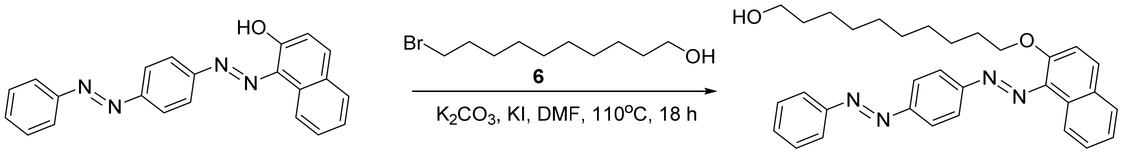

SudanIII　　SudanIII-Linker-A

Add Sudan Red III (1.056 g, 3 mmol), Compound 6 (10-bromo-1-decanol) (1.062 g, 4.5 mmol), potassium carbonate (2.07 g, 15 mmol) and potassium iodide (166 mg, 1 mmol) to a 100 mL flask, add DMF (15 mL), and stir at 110 °C for 16 hours. The reaction was cooled to room temperature, water (200 mL) was added, and extracted with ethyl acetate (80 mL × 2). The organic layers were combined and spin-dried, and the obtained product was purified by column chromatography (petroleum ether/ethyl acetate=5/1) to obtain a red oily product (1.3 g, yield: 85%).

Step 2: Preparation of **SudanIII-Linker-B**, 10-((1-((E)-(4-((E)-phenyldiazenyl)phenyl)diazenyl)naphthalen-2-yl)oxy)decyl methanesulfonate, MS m/z (ESI): 587.0 [M+H]$^+$.

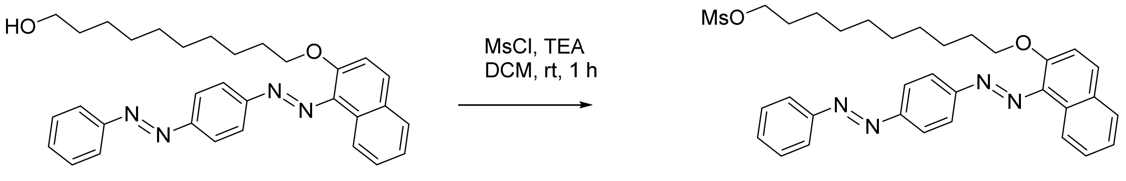

SudanIII-Linker-A　　SudanIII-Linker-B

The reactant intermediates SudanIII-Linker-A (3 g, 5.9 mmol) and triethylamine (1.2 g, 11.8 mmol) were dissolved in methylene chloride (20 mL), and methanesulfonyl chloride (808 mg, 7.08 mmol) was added dropwise. The mixture was stirred at room temperature for 1 hour. Water (50 mL) was added to the reaction liquid, and extracted with dichloromethane (50 mL×2). The organic layer was spin-dried, and the obtained product was purified by column chromatography (petroleum ether/ethyl acetate=3/1) to obtain a red oily product (1.8 g, yield: 52%).

Step 3: Preparation of **LD·ATTEC3 (C3)**, 3-(3,5-dibromo-4-hydroxybenzylidene)-5-iodo-1-(10-((1-((E)-(4-((E)-phenyldiazenyl)phenyl)diazenyl)naphthalen-2-yl)oxy)decyl)indolin-2-one

MS m/z (ESI): 1010.0 [M+H]$^+$.

^{1}H NMR (500 MHz, $CDCl_3$): δ 8.53 (s, 1H), 8.51 (d, *J*=8.0 Hz, 1H), 8.08-8.15 (m, 4H), 7.82-7.72-7.97 (m, 5H), 7.74 (s, 1H), 7.19-7.60 (m, 8H), 6.55 (t, *J*=8.4 Hz, 1H), 4.17-4.21 (m, 2H), 3.65-3.69 (m, 4H), 1.43-1.80 (m, 6H), 1.23-1.29 (m, 10H).

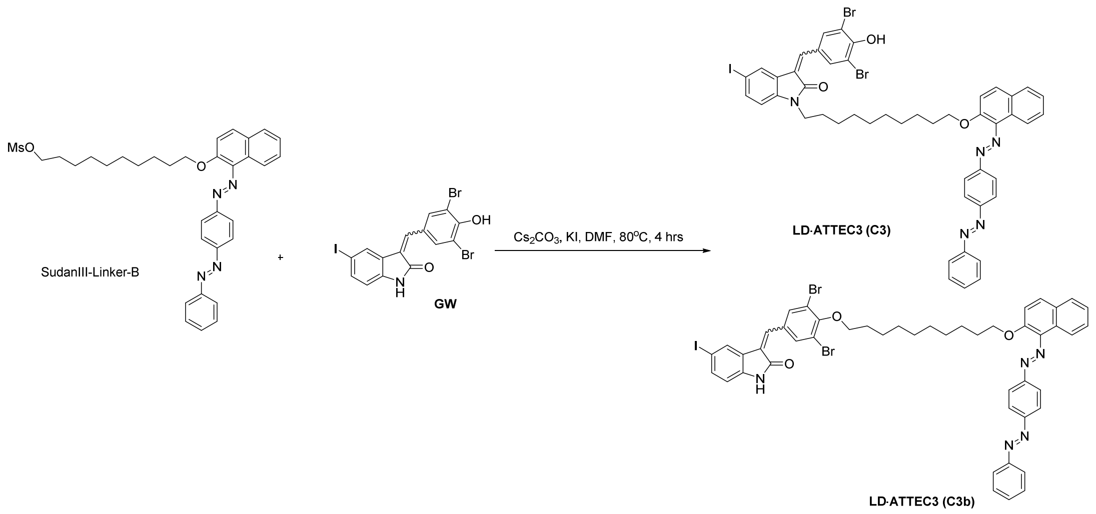

SudanIII-Linker-B 4 (587 mg, 1.0 mmol), GW (521 mg, 1.0 mmol), cesium carbonate (3.26 g, 10.0 mmol) and potassium iodide (166 mg, 1 mmol) were mixed in DMF (20 mL) solution and reacted at 50 °C for 3 hours. The reaction was cooled to room temperature, quenched with water (20 mL), extracted with ethyl acetate (80 mL × 2), the organic phase was separated, and the organic phase was washed with water (20 mL × 3) and saturated brine (50 mL × 1) , Dried over anhydrous sodium sulfate, filtered, and the filtrate was freed from the solvent under reduced pressure, and the resulting residue was purified by column chromatography (petroleum ether: ethyl acetate = 3:1) to obtain a red solid product (300 mg, 30%). There was also another low yield by-product with same molecular weight link to -OH not -NH, LD·ATTEC3b (C3b, 3-(3,5-dibromo-4-((10-((1-((E)-(4-((E)-phenyldiazenyl) phenyl)diazenyl)naphthalen-2-yl)oxy)decyl)oxy)benzylidene)-5-iodoindolin-2-one) synthesized and purified, with LC3B-binding capability as well (K_d = 14.3 ± 1.0 μM measured by MST; note that it has very poor solubility and the affinity could have been underestimated).

4. Synthesis of **LD·ATTEC4 (C4)**: 7-hydroxy-4-phenyl-5-((10-((1-((E)-(4-((E)-phenyldiazenyl)phenyl)diazenyl)naphthalen-2-yl)oxy)decyl)oxy)-2H-chromen-2-one

Step 1: Preparation of **LD·ATTEC-4 (C4)**, 7-hydroxy-4-phenyl-5-((10-((1-((E)-(4-((E)-phenyldiazenyl)phenyl)diazenyl)naphthalen-2-yl)oxy)decyl)oxy)-2H-chromen-2-one, MS m/z (ESI): 745.0 $[M+H]^+$.

^1H NMR (400 MHz, $CDCl_3$) δ 8.52 (d, J = 8.8 Hz, 1H), 8.09-8.16 (m, 4H), 7.82-7.97 (m, 4H), 7.39-7.56(m, 6H), 7.19-7.31 (m, 5H), 6.73 (bs, 1H), 6.61 (d, J = 2.4 Hz, 1H), 6.12 (d, J = 2.4 Hz, 1H), 5.93 (s, 1H), 4.21 (t, J = 6.4 Hz, 2H), 3.52 (t, J = 6.4 Hz, 2H), 1.79-1.83 (m, 2H), 0.77-1.48 (m, 14H).

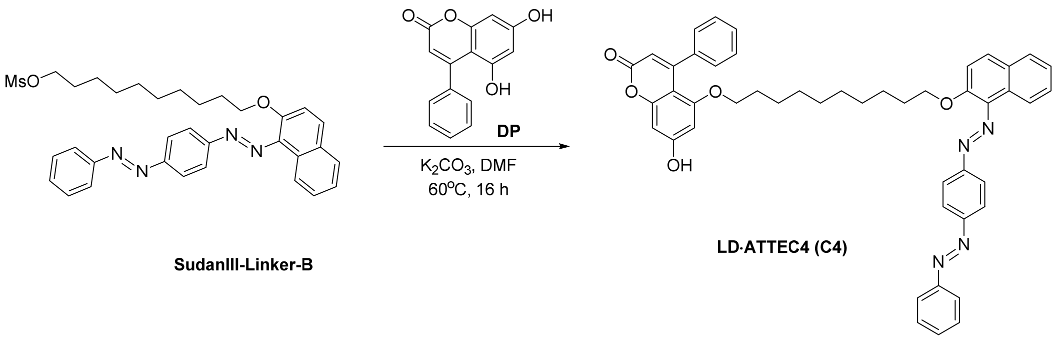

The reactant intermediate SudanIII-Linker-B (1.172 g, 2 mmol), DP (1.016 g, 4 mmoL) and potassium carbonate (1.656 g, 12 mmol) were added to a 100 mL flask, and DMF (10 mL) was added, stirred at 60 °C for 18 hours. The reaction solution was cooled to room temperature, water (50 mL) was added, and extracted with ethyl acetate (30 mL×2). The organic layer was combined and spin-dried, and the obtained product was purified by column chromatography (petroleum ether/ethyl acetate=2/1~1/1) to obtain red solid product LD·ATTEC-4 (201 mg, yield: 13%). At the same time, a red solid product LD·ATTEC-4A (mixed with disubstituted products) was obtained, and the mixture was further purified three times by preparative thin layer chromatography (prep-TLC) to obtain the pure product LD·ATTEC-4A (20 mg, yield: 1.3%).
