# Supplementary material for: Degradation of lipid droplets by chimeric autophagy-tethering compounds
Source: Cell Res. 2021 Jul 8;31(9):965–79. doi: 10.1038/s41422-021-00532-7 (PMC8410765; doi:10.1038/s41422-021-00532-7)
Supplement: Supplementary file 12 — Supplementary information, Data S2 [file 41422_2021_532_MOESM12_ESM.pdf]

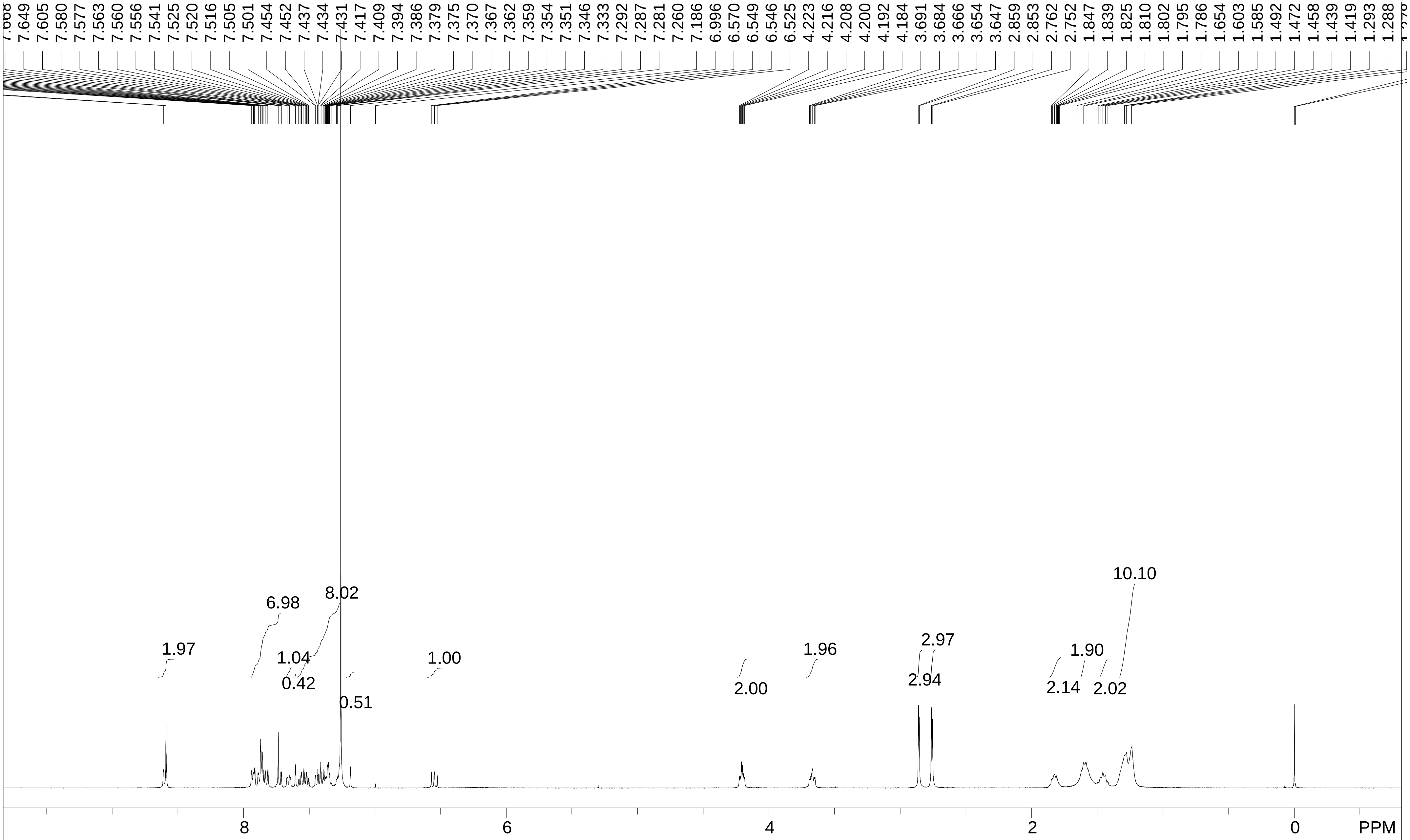

spect, CDCl3,

F1: 400.132	F2: 1.000	SW1: 8224		OF1: 2461.5		PTS1d: 65536	
EX: zg30		PW: 15.0 usec	PD: 1.0 sec	NA: 8	LB: 0.0		

LDATTEC-C1 1H NMR

NOESYPHSW CDCl3

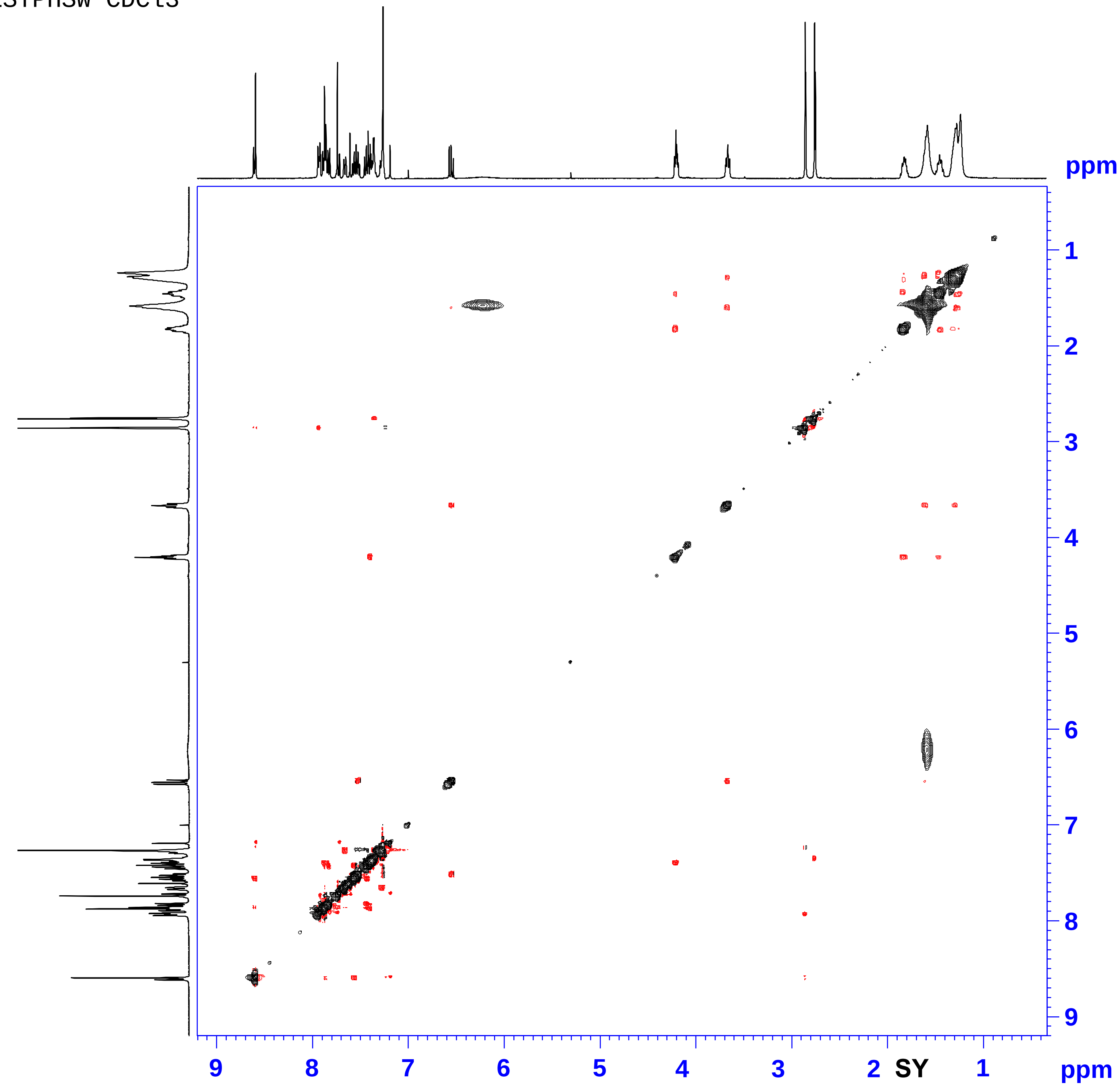

LDATTEC-C1 NOESY

NAME	CP-0027158-134-1-NOESY-CDCL3-20210326-T298	
EXPNO	10	
PROCNO	1	
Date_	20210326	
Time	21.54	
INSTRUM	nmrB005	
PROBHD	5 mm PABBO BB-	
PULPROG	noesyph	
TD	2048	
SOLVENT	CDCl3	
NS	8	
DS	4	
SWH	3546.099	Hz
FIDRES	1.731494	Hz
AQ	0.2888180	sec
RG	203	
DW	141.000	usec
DE	6.50	usec
TE	295.5	K
D0	0.00012445	sec
D1	1.96190703	sec
D8	0.80000001	sec
IN0	0.00028200	sec

======== CHANNEL f1 ========

NUC1	1H	
P1	13.00	usec
PL1	-1.00	dB
PL1W	11.89016438	W
SFO1	400.1319179	MHz
ND0	1	
TD	256	
SFO1	400.1319	MHz
FIDRES	13.851951	Hz
SW	8.862	ppm
FnMODE	States-TPPI	
SI	1024	
SF	400.1300096	MHz
WDW	QSINE	
SSB	2	
LB	0.00	Hz
GB	0	
PC	1.00	
SI	1024	
MC2	States-TPPI	
SF	400.1300096	MHz
WDW	QSINE	
SSB	2	
LB	0.00	Hz
GB	0	

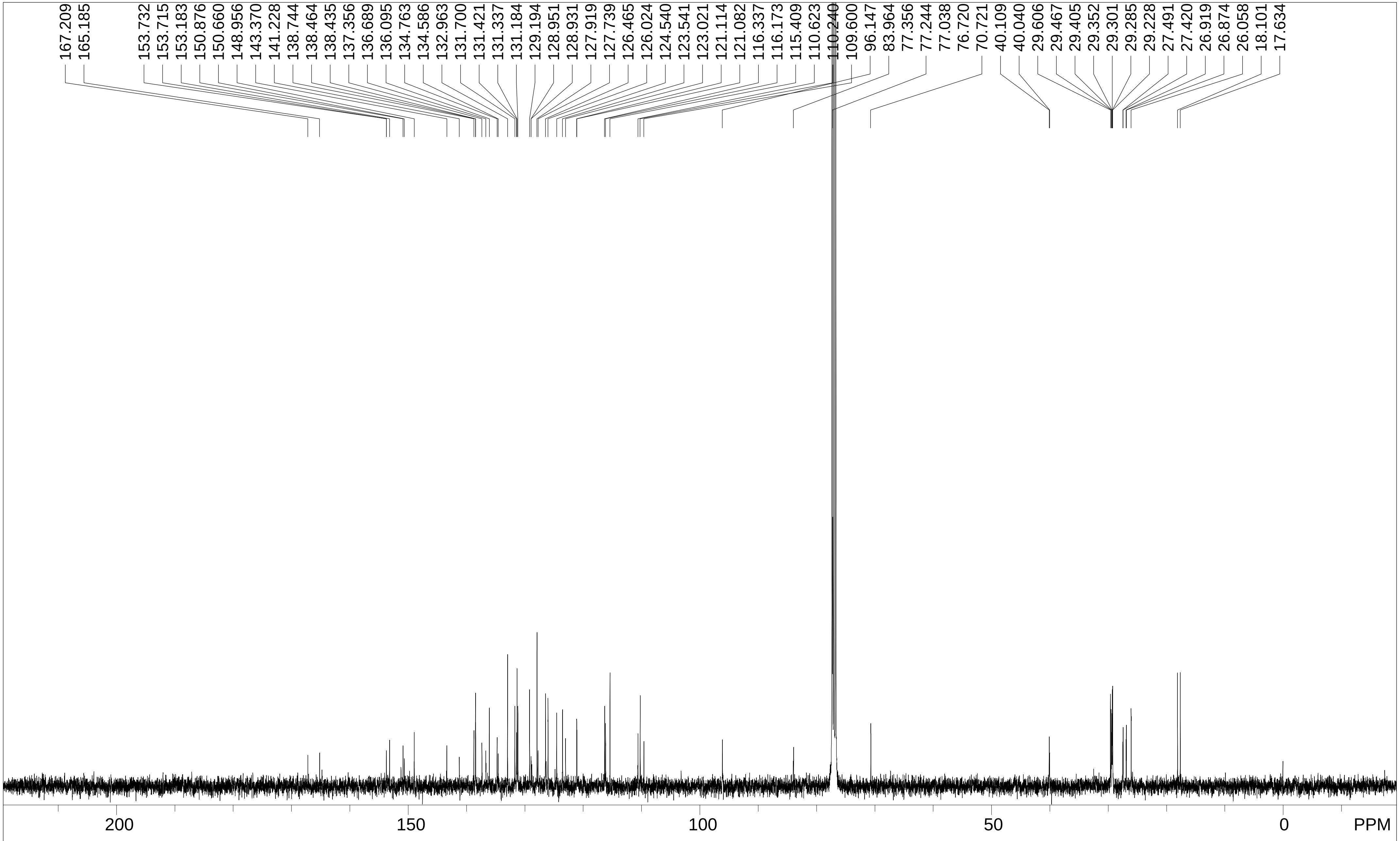

spect, CDCl3,

F1: 100.623	F2: 1.000	SW1: 24038		OF1: 10062.5		PTS1d: 32768	
EX: zgpg30		PW: 10.0 usec	PD: 2.0 sec	NA: 1024	LB: 0.0		

LDATTEC-C1 13C NMR

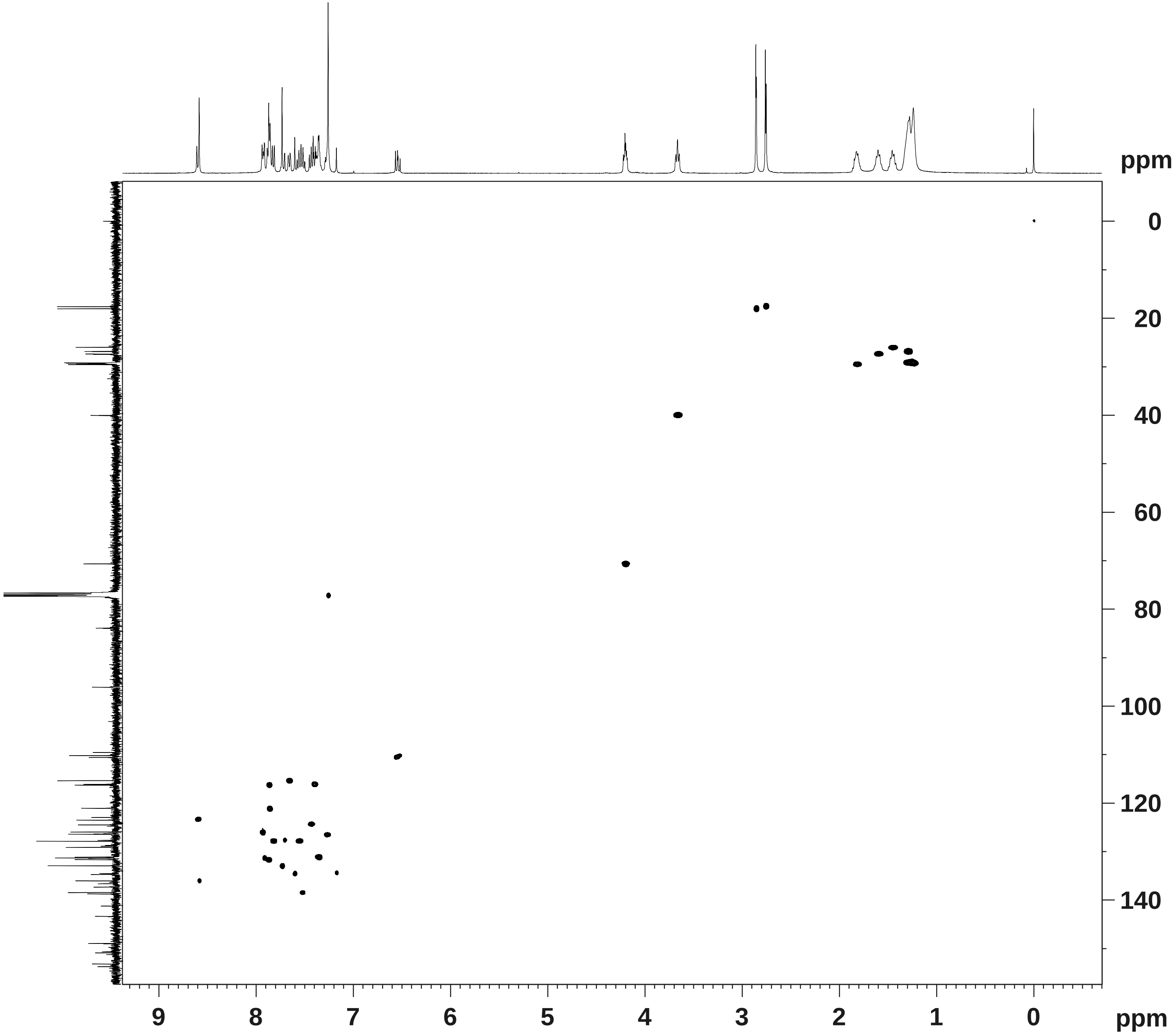


```
Current Data Parameters
NAME     CP-0027158-134-2-HSQC-CDCL3-20210331-T377
EXPNO              4
PROCNO             1

F2 - Acquisition Parameters
Date_          20210331
Time              23.26
INSTRUM           spect
PROBHD   5 mm PABBO BB/
PULPROG        hsqcetgp
TD                 1024
SOLVENT           CDCl3
NS                    4
DS                   16
SWH            4032.258 Hz
FIDRES         3.937752 Hz
AQ            0.1269760 sec
RG                  203
DW              124.000 usec
DE                 6.50 usec
TE                296.5 K
CNST2       145.0000000
D0           0.00000300 sec
D1           1.46887004 sec
D4           0.00172414 sec
D11          0.03000000 sec
D16          0.00020000 sec
IN0          0.00003000 sec
ZGOPTNS

======== CHANNEL f1 ========
SFO1        400.1317457 MHz
NUC1                 1H
P1                15.00 usec
P2                30.00 usec
P28             1000.00 usec
PLW1        11.00000000 W

======== CHANNEL f2 ========
SFO2        100.6202727 MHz
NUC2                13C
CPDPRG[2           garp
P3                10.00 usec
P4                20.00 usec
PCPD2             80.00 usec
PLW2        50.00000000 W
PLW12        0.78125000 W

====== GRADIENT CHANNEL =====
GPNAM[1]     SMSQ10.100
GPNAM[2]     SMSQ10.100
GPZ1              80.00 %
GPZ2              20.10 %
P16             1000.00 usec

F1 - Acquisition parameters
TD                  256
SFO1           100.6203 MHz
FIDRES        65.104164 Hz
SW              165.639 ppm
FnMODE    Echo-Antiecho

F2 - Processing parameters
SI                 1024
SF          400.1300111 MHz
WDW               QSINE
SSB                   2
LB       0 Hz
GB       0
PC                 1.40

F1 - Processing parameters
SI                 1024
MC2       echo-antiecho
SF          100.6127690 MHz
WDW               QSINE
SSB                   2
LB       0 Hz
GB       0
```

LDATTEC-C1 HSQC

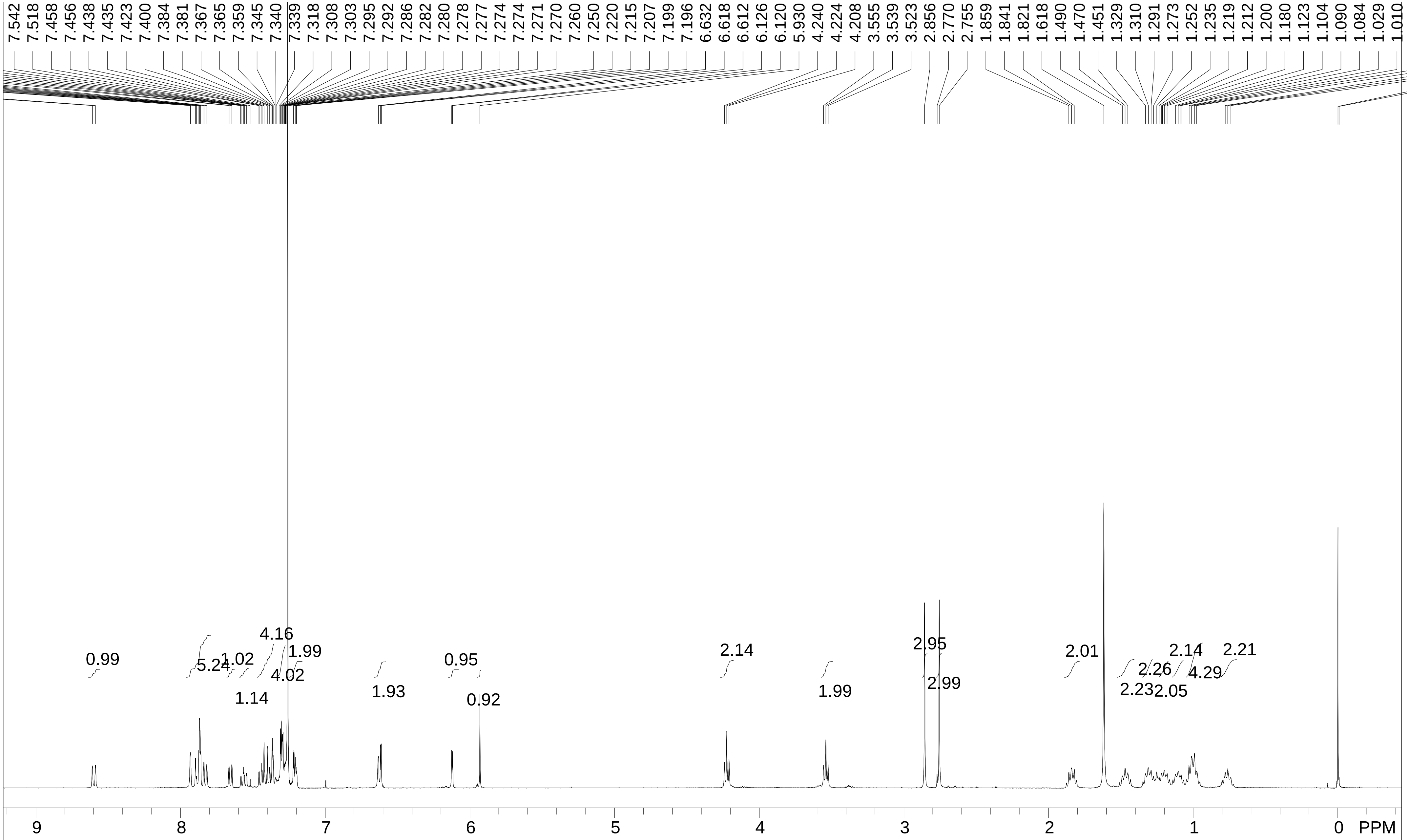

nmrB005, CDCl3,

F1: 400.132	F2: 1.000	SW1: 8224		OF1: 2461.3		PTS1d: 32768	
EX: zg30		PW: 13.0 usec	PD: 1.0 sec	NA: 32	LB: 0.0		

LDATTEC-C2 1H NMR

NOESYPHSW CDCl3

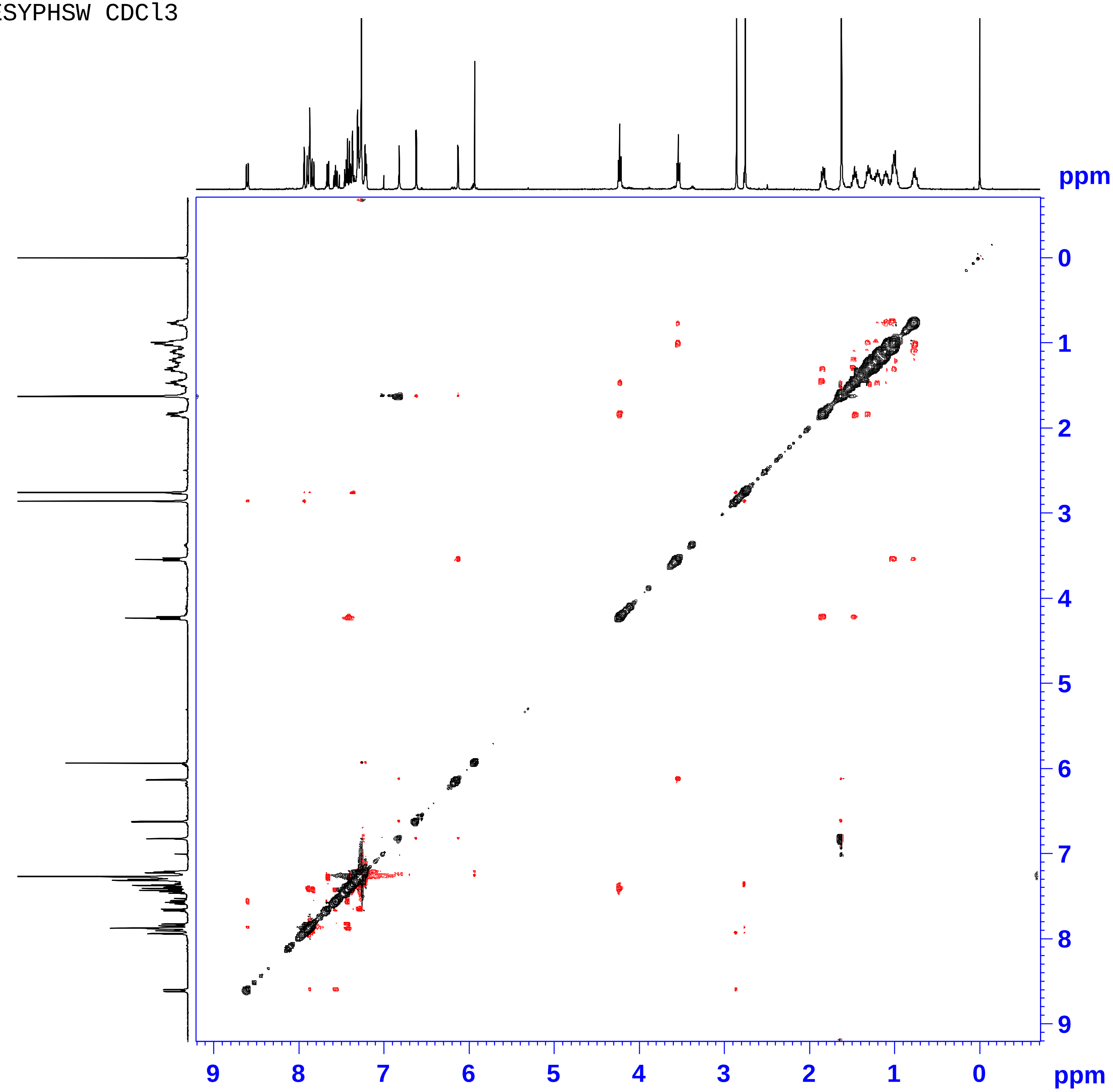

LDATTEC-C2 NOESY

```
NAME      CP-0027158-135-4-NOESY-CDCL3-20210326-T298
EXPNO                  10
PROCNO                  1
Date_            20210327
Time                 5.35
INSTRUM           nmrB005
PROBHD   5 mm PABBO BB-
PULPROG           noesyph
TD                   2048
SOLVENT             CDCl3
NS                      8
DS                      4
SWH              3968.254 Hz
FIDRES           1.937624 Hz
AQ              0.2580980 sec
RG                    203
DW                126.000 usec
DE                   6.50 usec
TE                  294.7 K
D0             0.00010945 sec
D1             1.99262702 sec
D8             0.80000001 sec
IN0            0.00025200 sec

======== CHANNEL f1 ========
NUC1                   1H
P1                  13.00 usec
PL1                 -1.00 dB
PL1W          11.89016438 W
SFO1          400.1317089 MHz
ND0                     1
TD                    256
SFO1             400.1317 MHz
FIDRES          15.500992 Hz
SW                  9.917 ppm
FnMODE        States-TPPI
SI                   1024
SF            400.1300091 MHz
WDW                 QSINE
SSB                     2
LB                   0.00 Hz
GB                      0
PC                   1.00
SI                   1024
MC2           States-TPPI
SF            400.1300091 MHz
WDW                 QSINE
SSB                     2
LB                   0.00 Hz
GB                      0
```

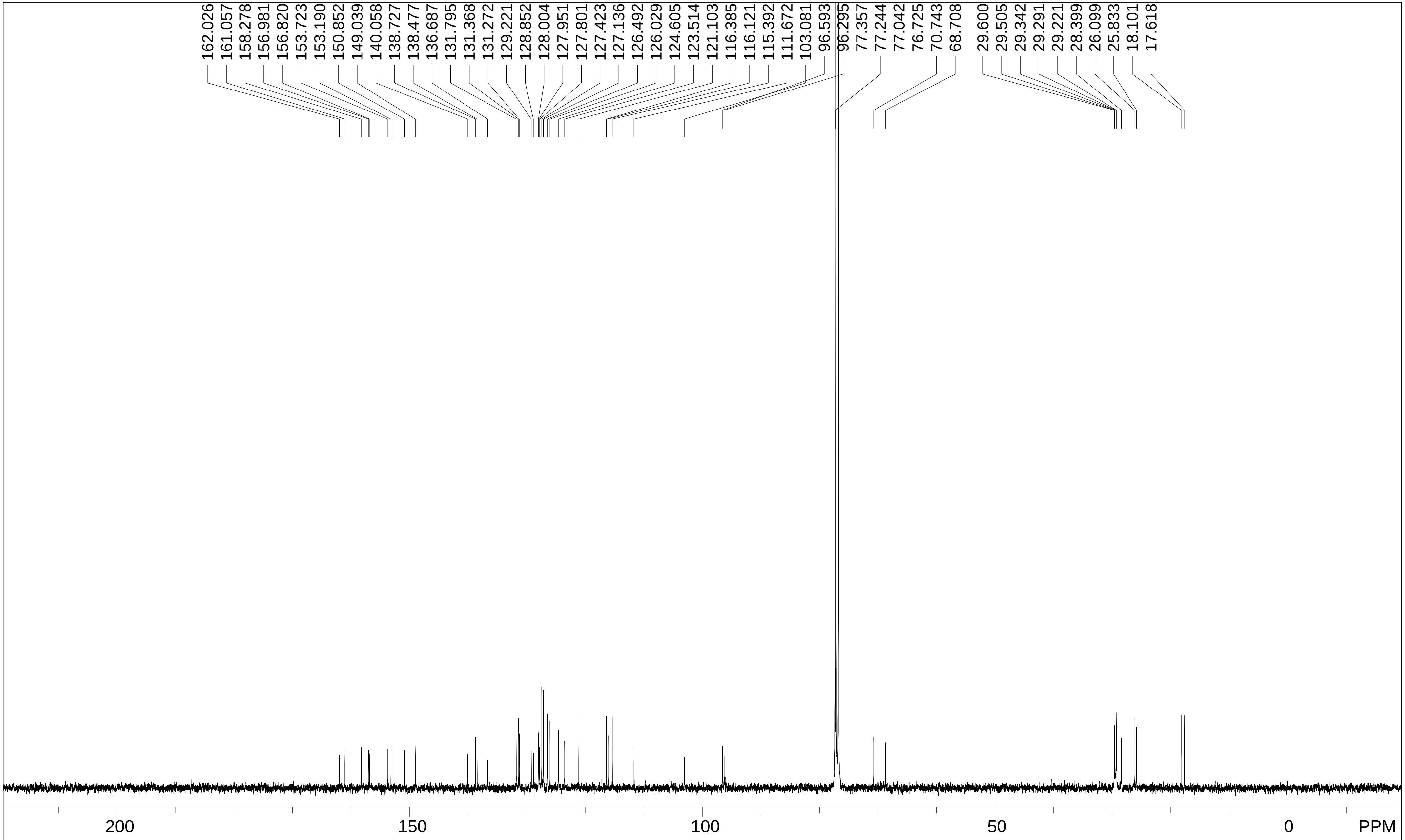

spect, CDCl3,

F1: 100.623	F2: 1.000	SW1: 24038		OF1: 10062.5		PTS1d: 32768	
EX: zgpg30		PW: 10.0 usec	PD: 2.0 sec	NA: 1024	LB: 0.0		

LDATTEC-C2 13C NMR

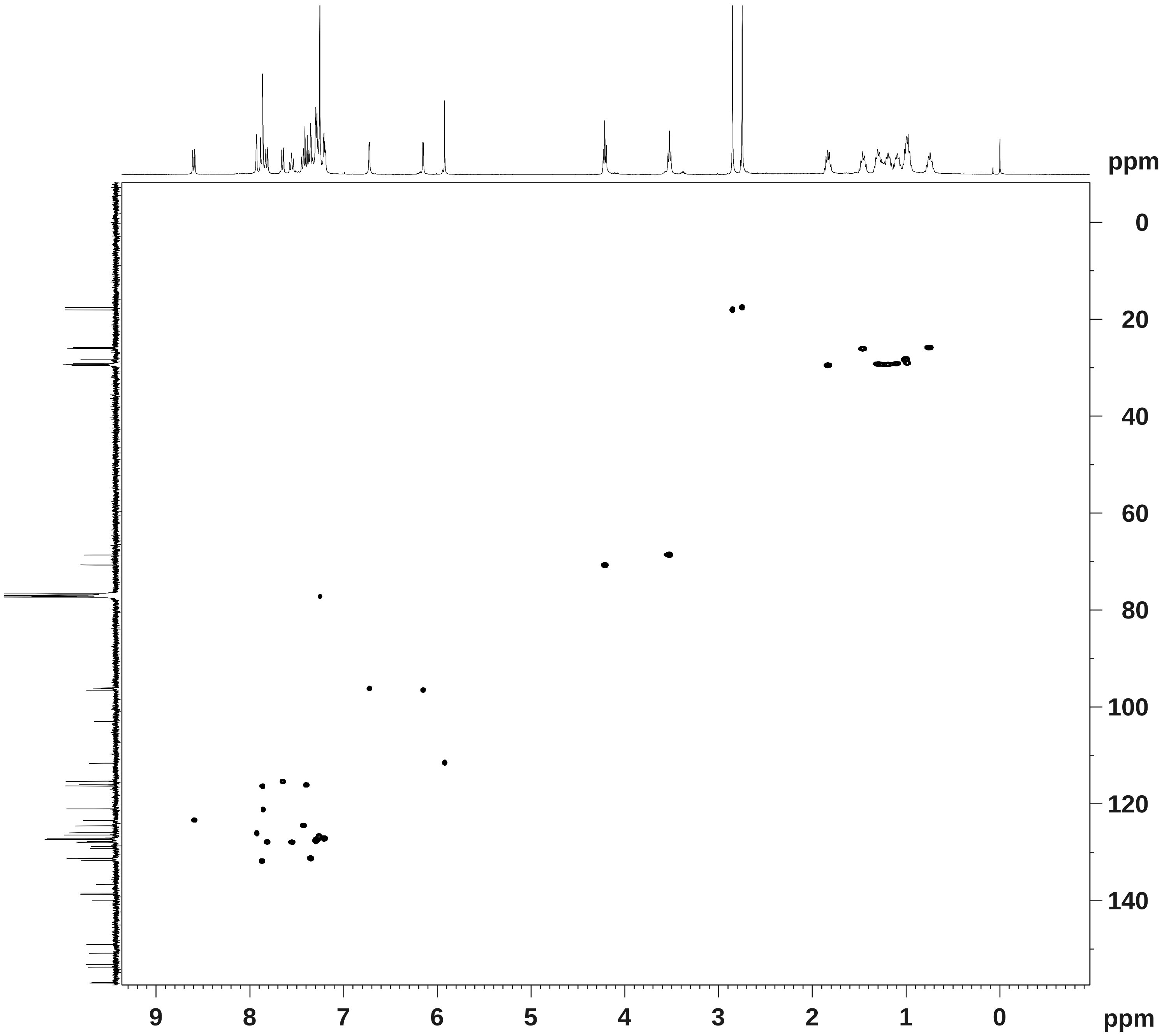

LDATTEC-C2 HSQC

```
Current Data Parameters
NAME     CP-0027158-135-2-HSQC-CDCL3-20210331-T377
EXPNO                 3
PROCNO                1

F2 - Acquisition Parameters
Date_          20210331
Time              21.54
INSTRUM           spect
PROBHD   5 mm PABBO BB/
PULPROG        hsqcetgp
TD                 1024
SOLVENT            CDCl3
NS                    4
DS                   16
SWH            4132.231 Hz
FIDRES         4.035382 Hz
AQ            0.1239040 sec
RG                  203
DW              121.000 usec
DE                 6.50 usec
TE                296.5 K
CNST2       145.0000000
D0           0.00000300 sec
D1           1.47194195 sec
D4           0.00172414 sec
D11          0.03000000 sec
D16          0.00020000 sec
IN0          0.00003000 sec
ZGOPTNS

======== CHANNEL f1 ========
SFO1        400.1316931 MHz
NUC1                 1H
P1                15.00 usec
P2                30.00 usec
P28             1000.00 usec
PLW1        11.00000000 W

======== CHANNEL f2 ========
SFO2        100.6202727 MHz
NUC2                13C
CPDPRG[2           garp
P3                10.00 usec
P4                20.00 usec
PCPD2             80.00 usec
PLW2        50.00000000 W
PLW12        0.78125000 W

====== GRADIENT CHANNEL =====
GPNAM[1]     SMSQ10.100
GPNAM[2]     SMSQ10.100
GPZ1              80.00 %
GPZ2              20.10 %
P16             1000.00 usec

F1 - Acquisition parameters
TD                  256
SFO1           100.6203 MHz
FIDRES        65.104164 Hz
SW              165.639 ppm
FnMODE    Echo-Antiecho

F2 - Processing parameters
SI                 1024
SF          400.1300118 MHz
WDW               QSINE
SSB                   2
LB       0 Hz
GB       0
PC                 1.40

F1 - Processing parameters
SI                 1024
MC2        echo-antiecho
SF          100.6127690 MHz
WDW               QSINE
SSB                   2
LB       0 Hz
GB       0
```

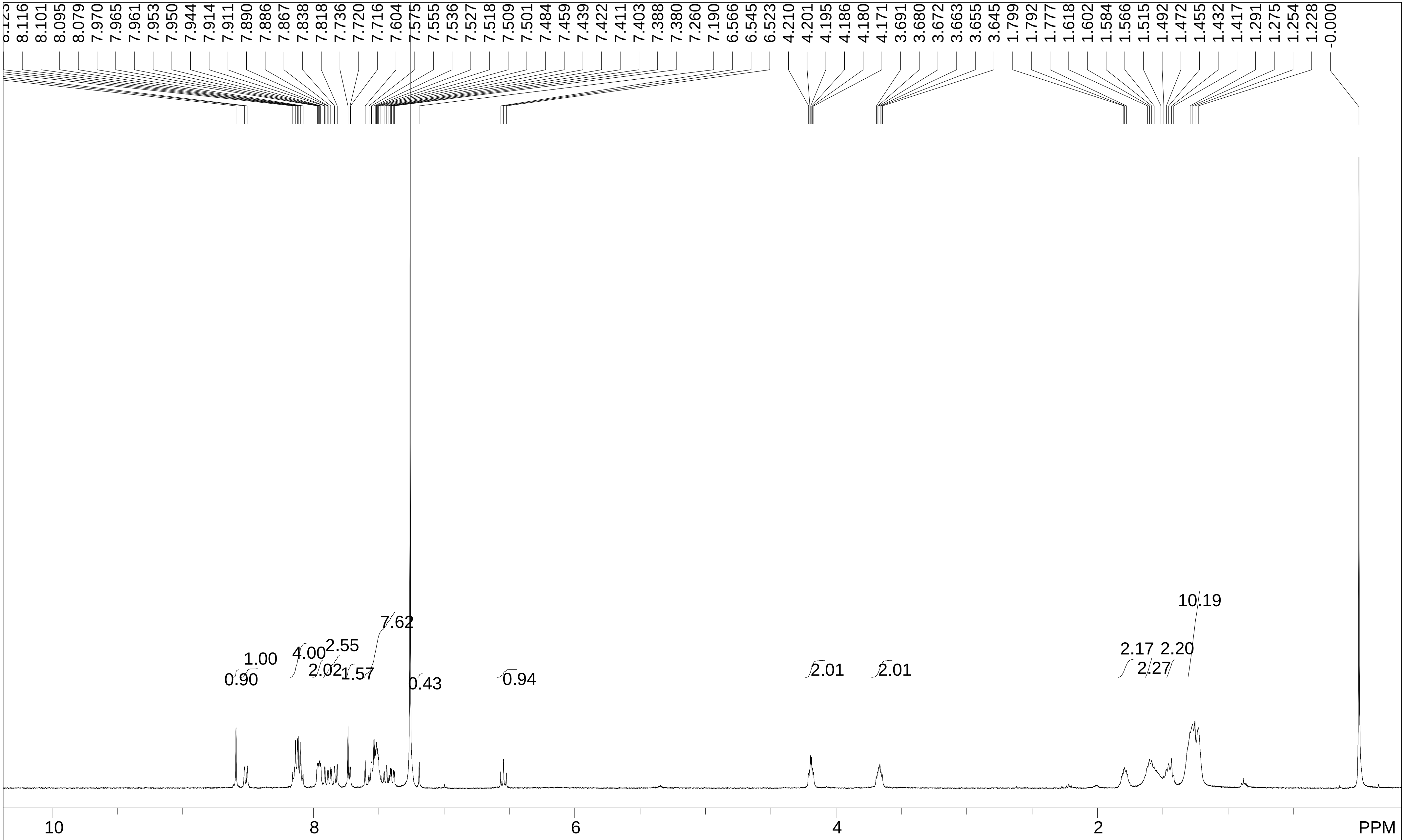

spect, CDCl3,

F1: 400.132	F2: 1.000	SW1: 8224		OF1: 2460.9		PTS1d: 65536	
EX: zg30		PW: 15.0 usec	PD: 1.0 sec	NA: 8	LB: 0.0		

LDATTEC-C3 1H NMR

NOESYPHSW CDCl3

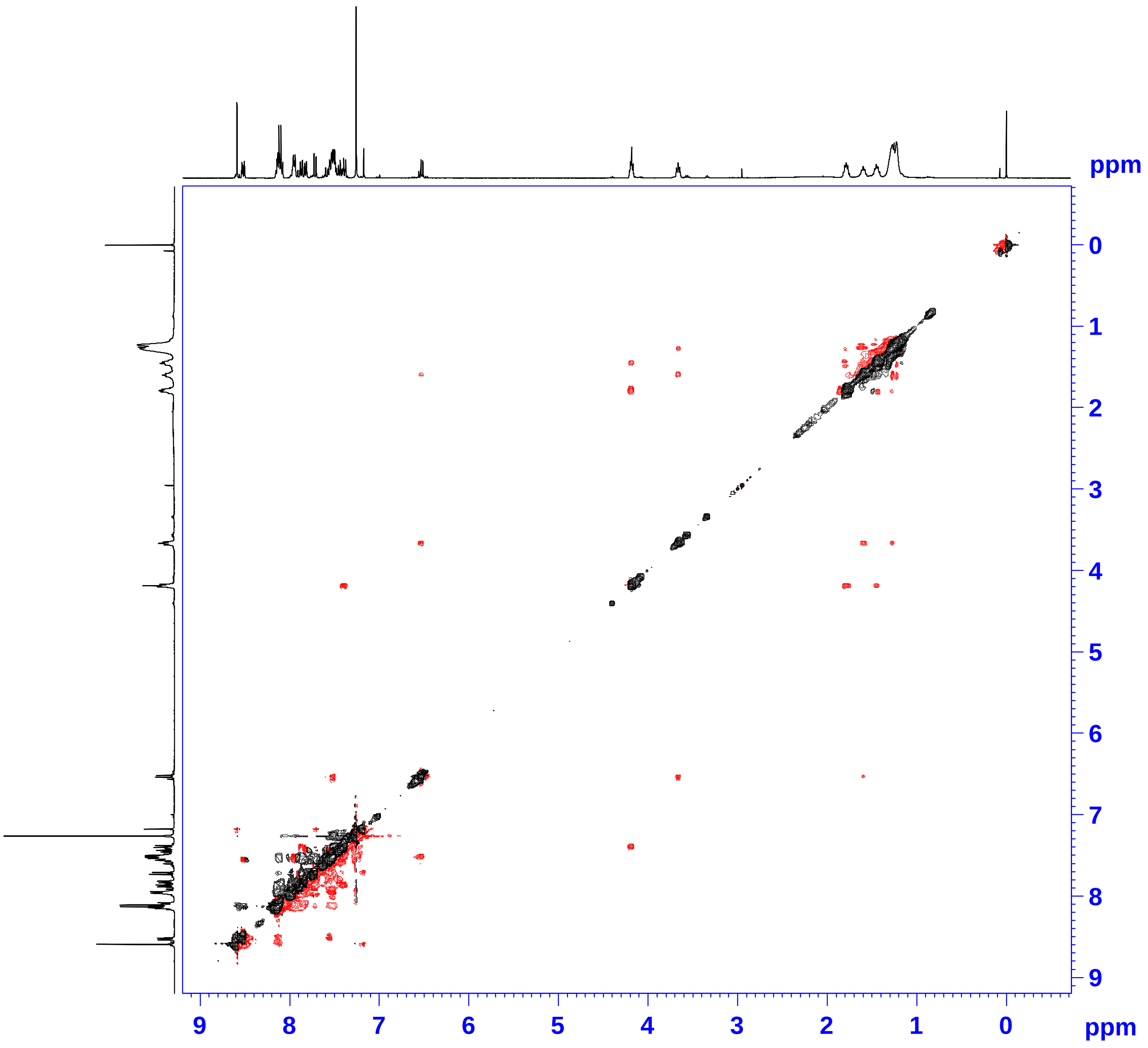


```
NAME     CP-0027158-057-3-NOESY-CDCL3-20210318-T298
EXPNO                 10
PROCNO                 1
Date_           20210318
Time               19.09
INSTRUM          nmrB005
PROBHD   5 mm PABBO BB-
PULPROG          noesyph
TD                  2048
SOLVENT            CDCl3
NS                     8
DS                     4
SWH             3968.254 Hz
FIDRES          1.937624 Hz
AQ             0.2580980 sec
RG                   161
DW               126.000 usec
DE                  6.50 usec
TE                 295.3 K
D0            0.00010945 sec
D1            1.99262702 sec
D8            0.80000001 sec
IN0           0.00025200 sec

======== CHANNEL f1 ========
NUC1                  1H
P1                 13.00 usec
PL1                -1.00 dB
PL1W         11.89016438 W
SFO1         400.1317047 MHz
ND0                    1
TD                   256
SFO1            400.1317 MHz
FIDRES         15.500992 Hz
SW                 9.917 ppm
FnMODE       States-TPPI
SI                  1024
SF           400.1300098 MHz
WDW                QSINE
SSB                    2
LB                  0.00 Hz
GB                     0
PC                  1.00
SI                  1024
MC2          States-TPPI
SF           400.1300098 MHz
WDW                QSINE
SSB                    2
LB                  0.00 Hz
GB                     0
```

LDATTEC-C3 NOESY

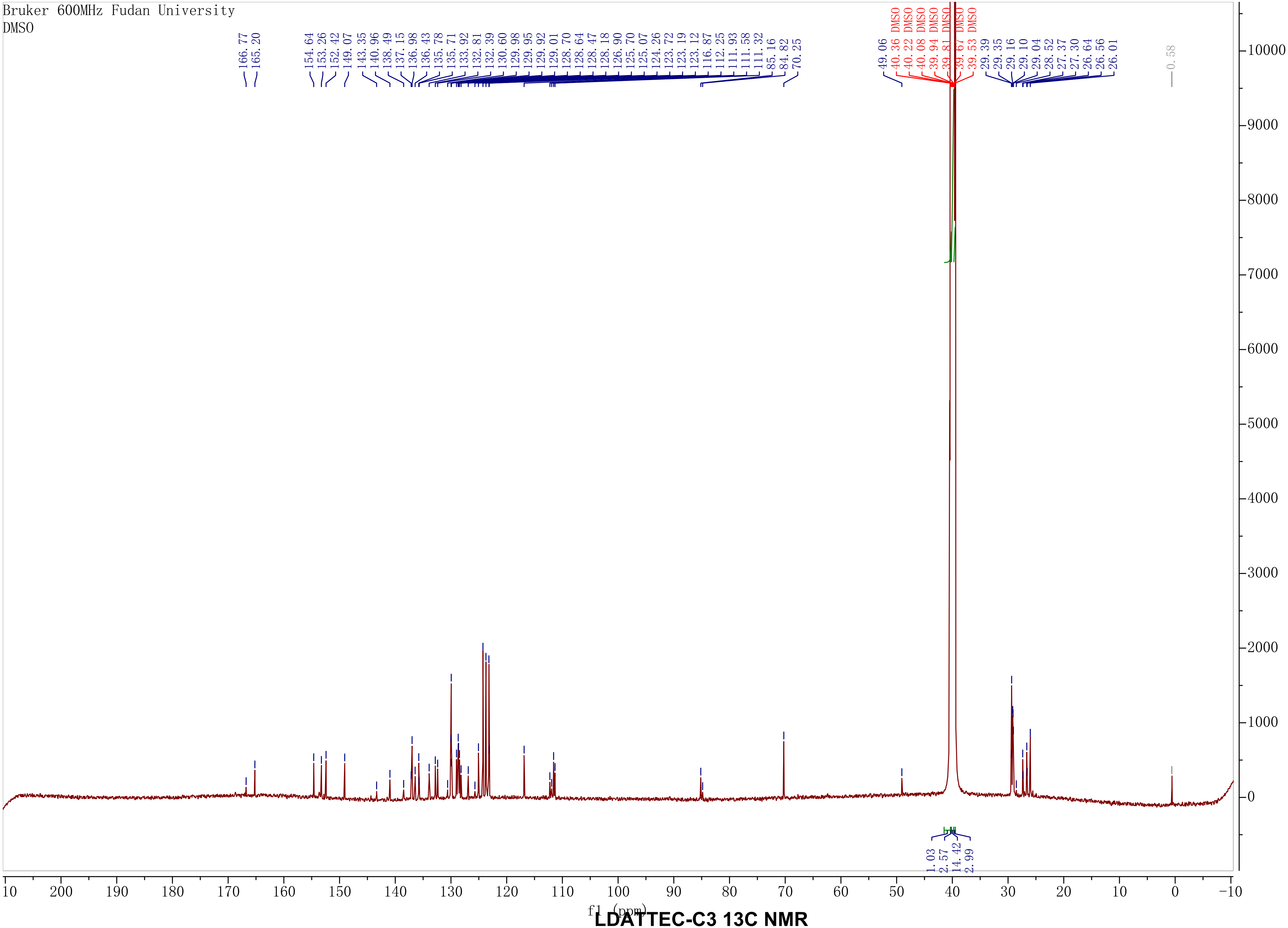

LDATTEC-C3 13C NMR

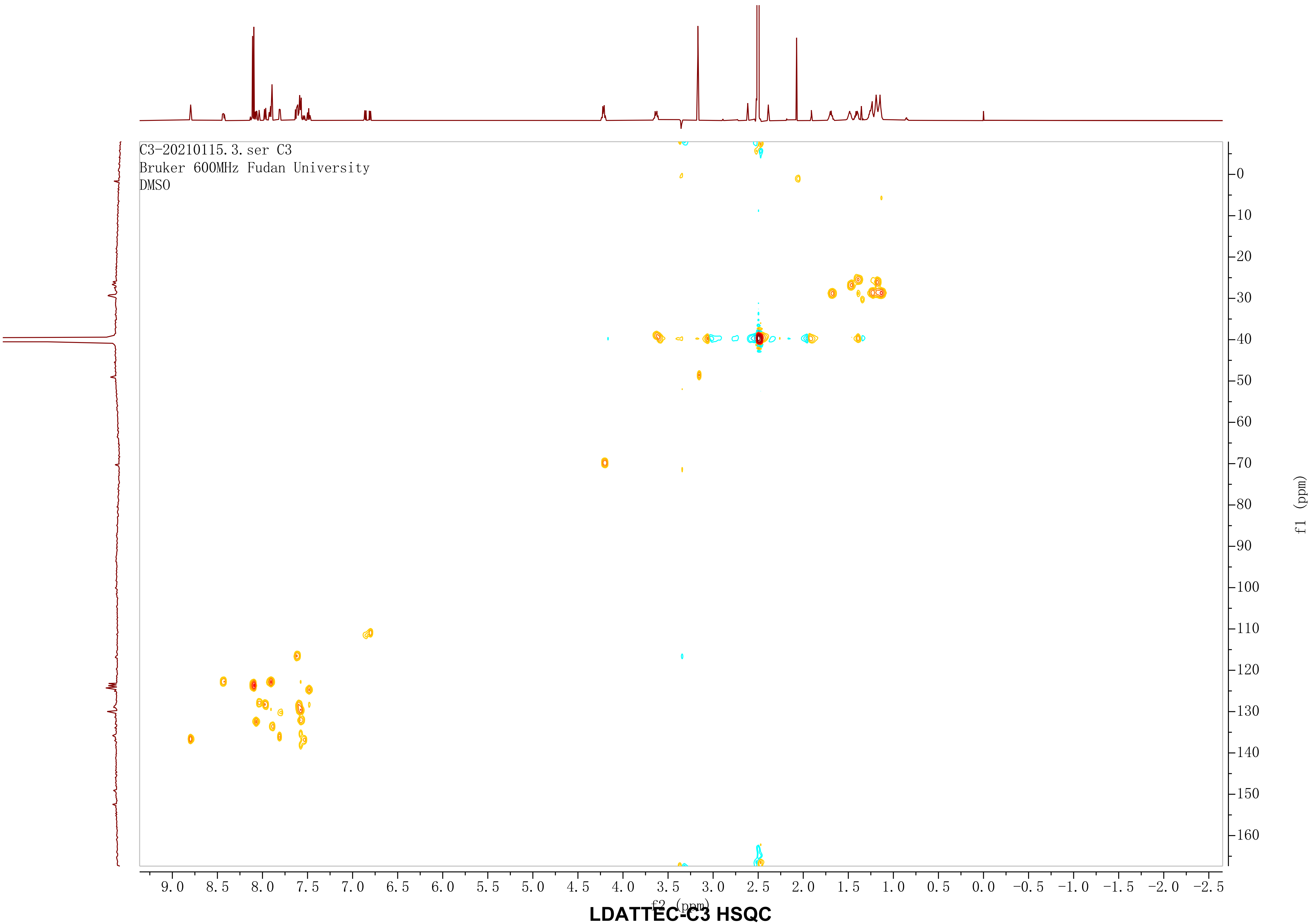

LDATTEC-C3 HSQC

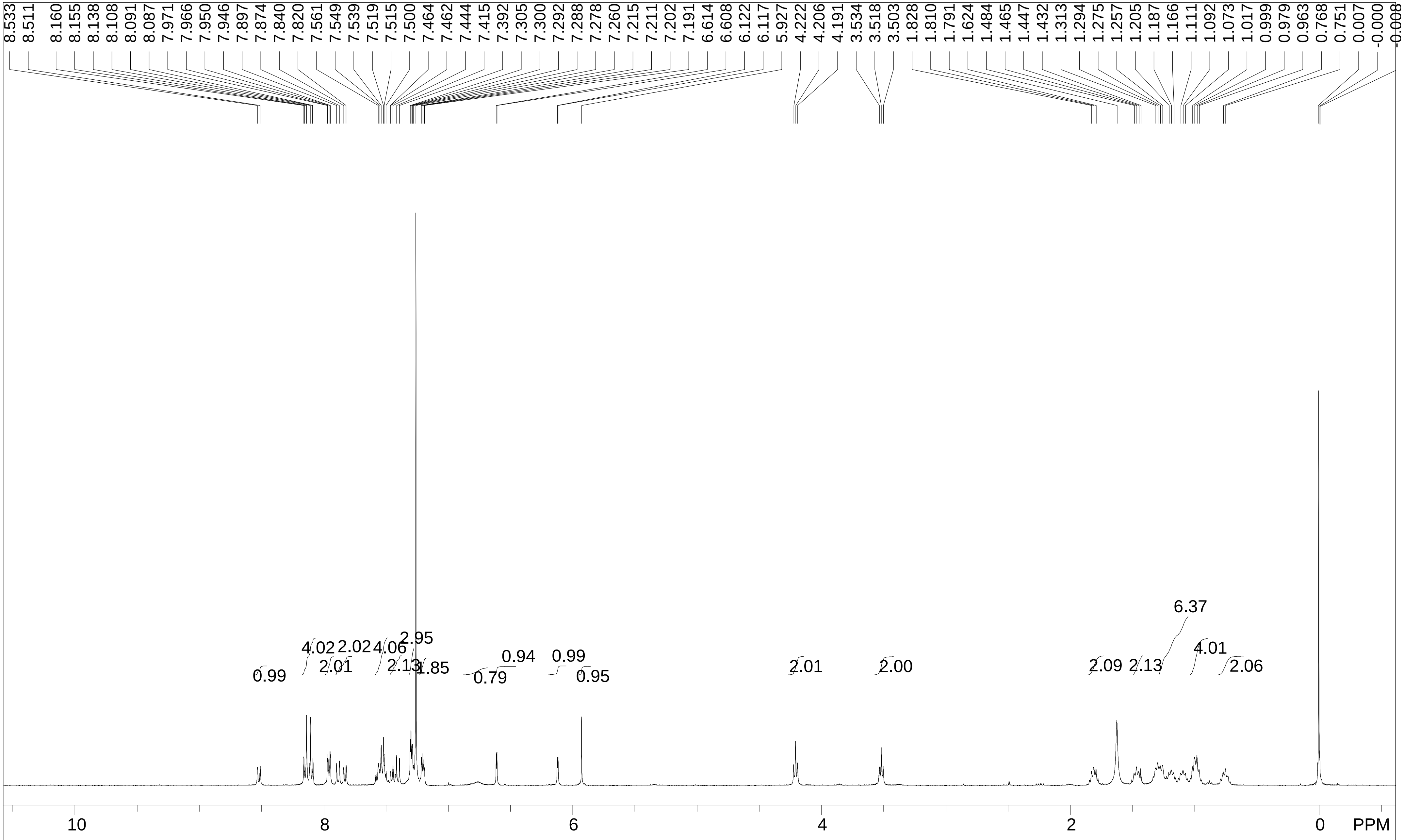

spect, CDCl3,

F1: 400.132	F2: 1.000	SW1: 8224		OF1: 2461.2		PTS1d: 65536
EX: zg30		PW: 15.0 usec	PD: 1.0 sec	NA: 8	LB: 0.0	

LDATTEC-C4 1H NMR

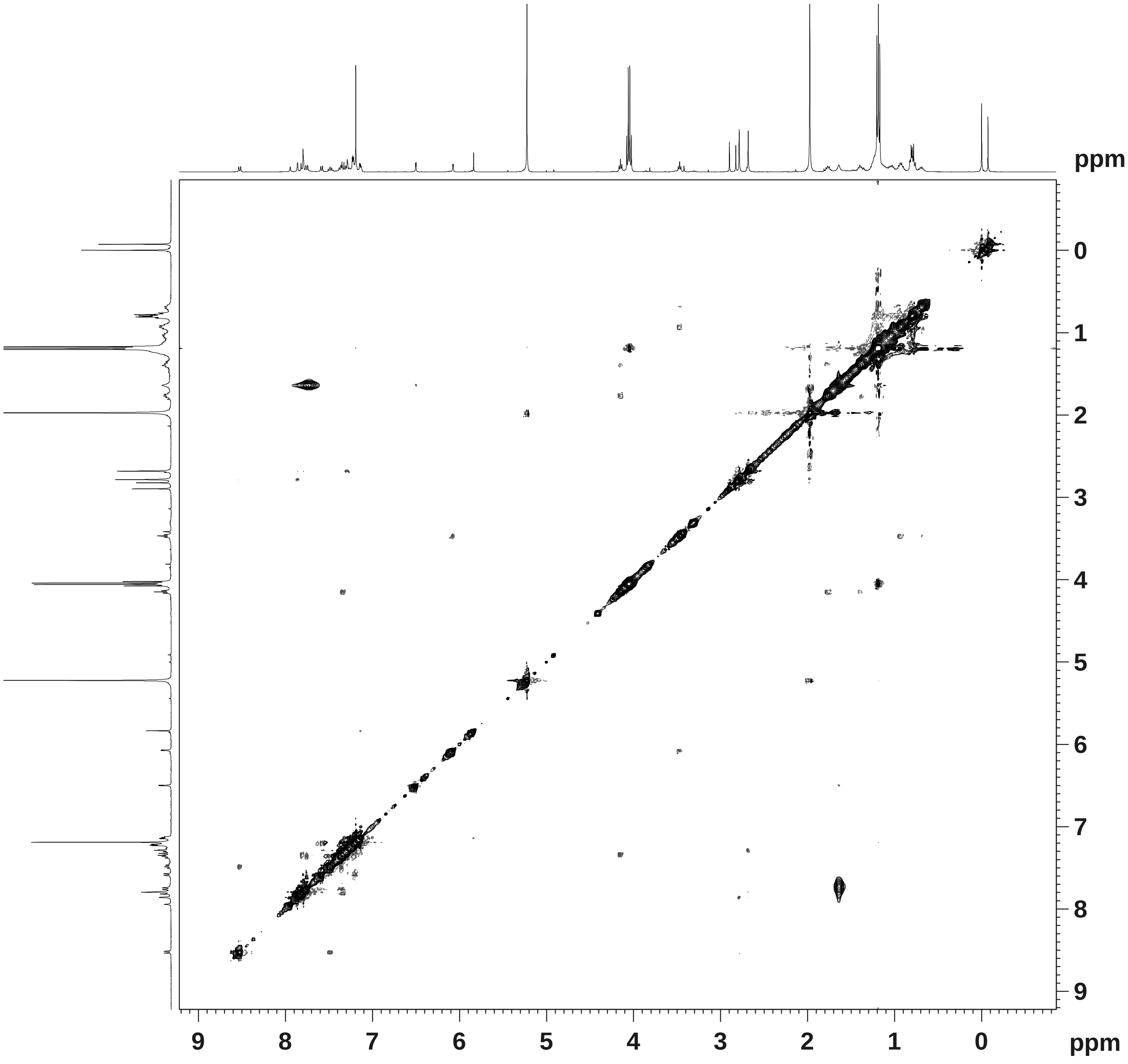

F2 - Acquisition Parameters
Date_ 20200422
Time 4.58
INSTRUM spect
PROBHD 5 mm PABBO BB/
PULPROG noesygpphpp
TD 2048
SOLVENT CDCl3
NS 4
DS 32
SWH 4032.258 Hz
FIDRES 1.968876 Hz
AQ 0.2539520 sec
RG 57
DW 124.000 usec
DE 6.50 usec
TE 296.3 K
D0 0.00010490 sec
D1 2.00204802 sec
D8 0.80000001 sec
D11 0.03000000 sec
D12 0.00002000 sec
D16 0.00020000 sec
IN0 0.00024800 sec

======== CHANNEL f1 ========
SFO1 400.1317106 MHz
NUC1 1H
P1 15.00 usec
P2 30.00 usec
P17 2500.00 usec
PLW1 11.00000000 W
PLW10 3.66120005 W

====== GRADIENT CHANNEL =====
GPNAM[1] SMSQ10.100
GPZ1 40.00 %
P16 1000.00 usec

F1 - Acquisition parameters
TD 256
SFO1 400.1317 MHz
FIDRES 15.751008 Hz
SW 10.077 ppm
FnMODE States-TPPI

F2 - Processing parameters
SI 1024
SF 400.1300373 MHz
WDW QSINE
SSB 2
LB 0 Hz
GB 0
PC 1.00

F1 - Processing parameters
SI 1024
MC2 States-TPPI
SF 400.1300373 MHz
WDW QSINE
SSB 2
LB 0 Hz
GB 0

LDATTEC-C4 NOESY

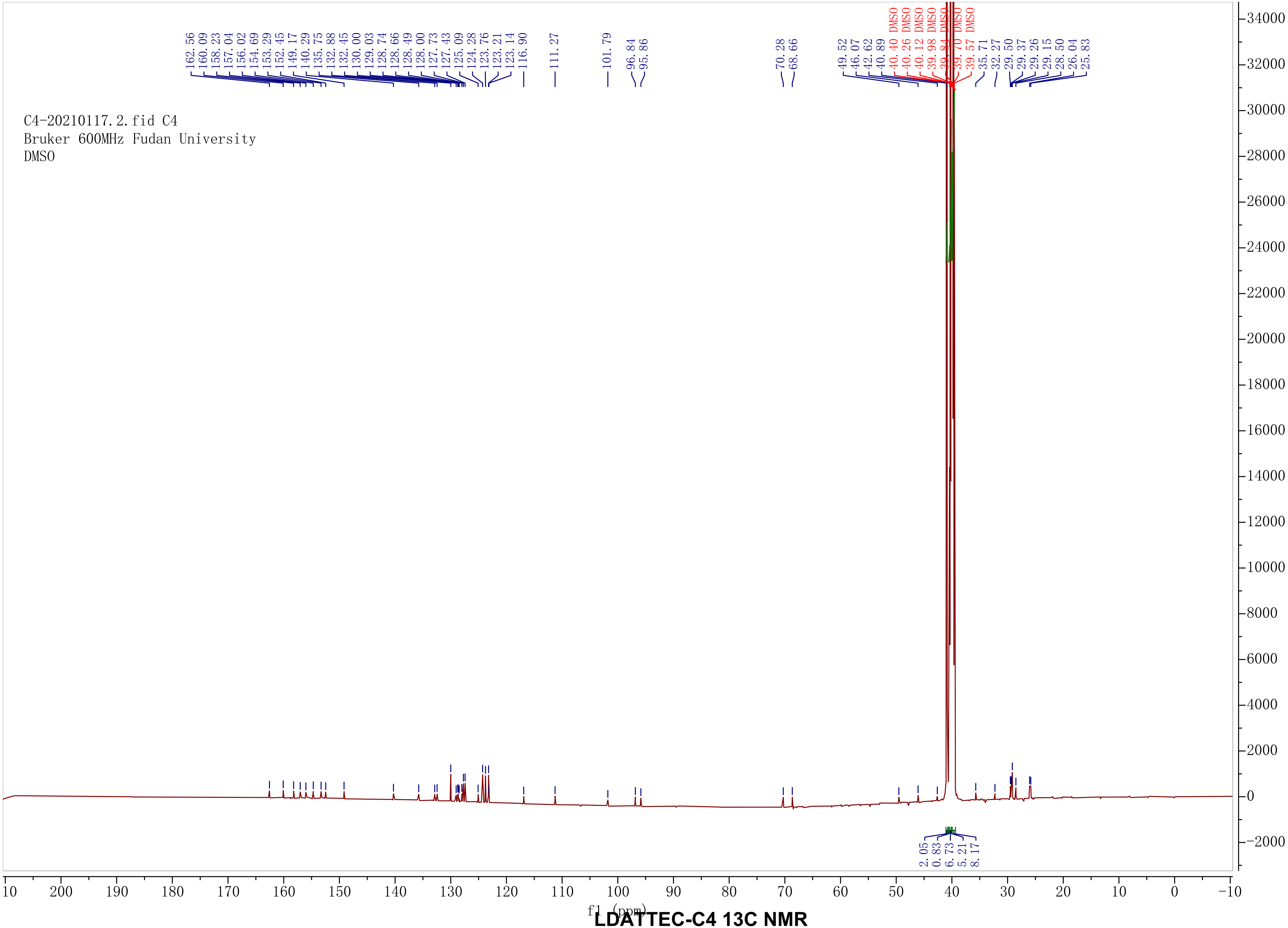

LDATTEC-C4 13C NMR

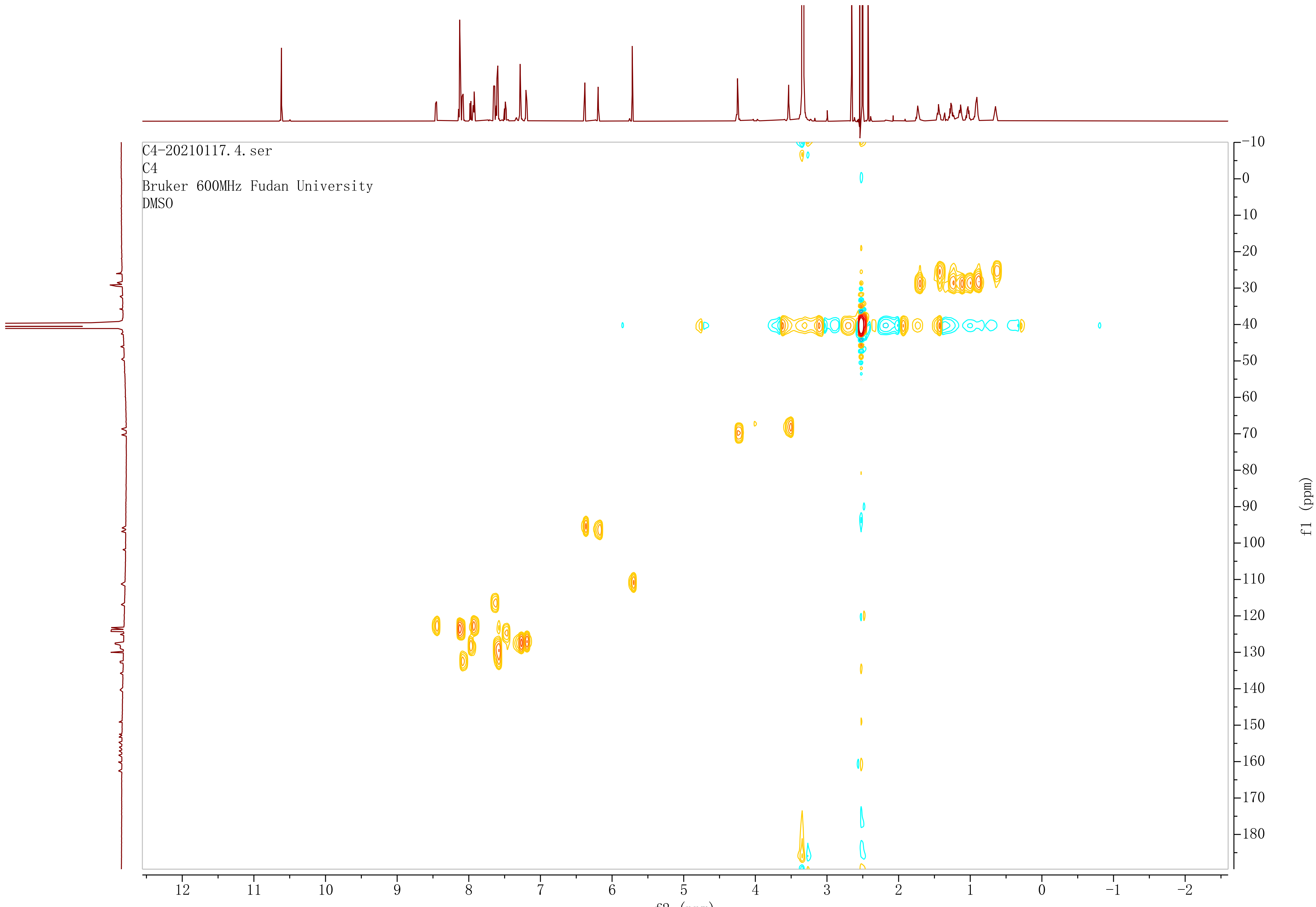

LDATTEC-C4 HSQC

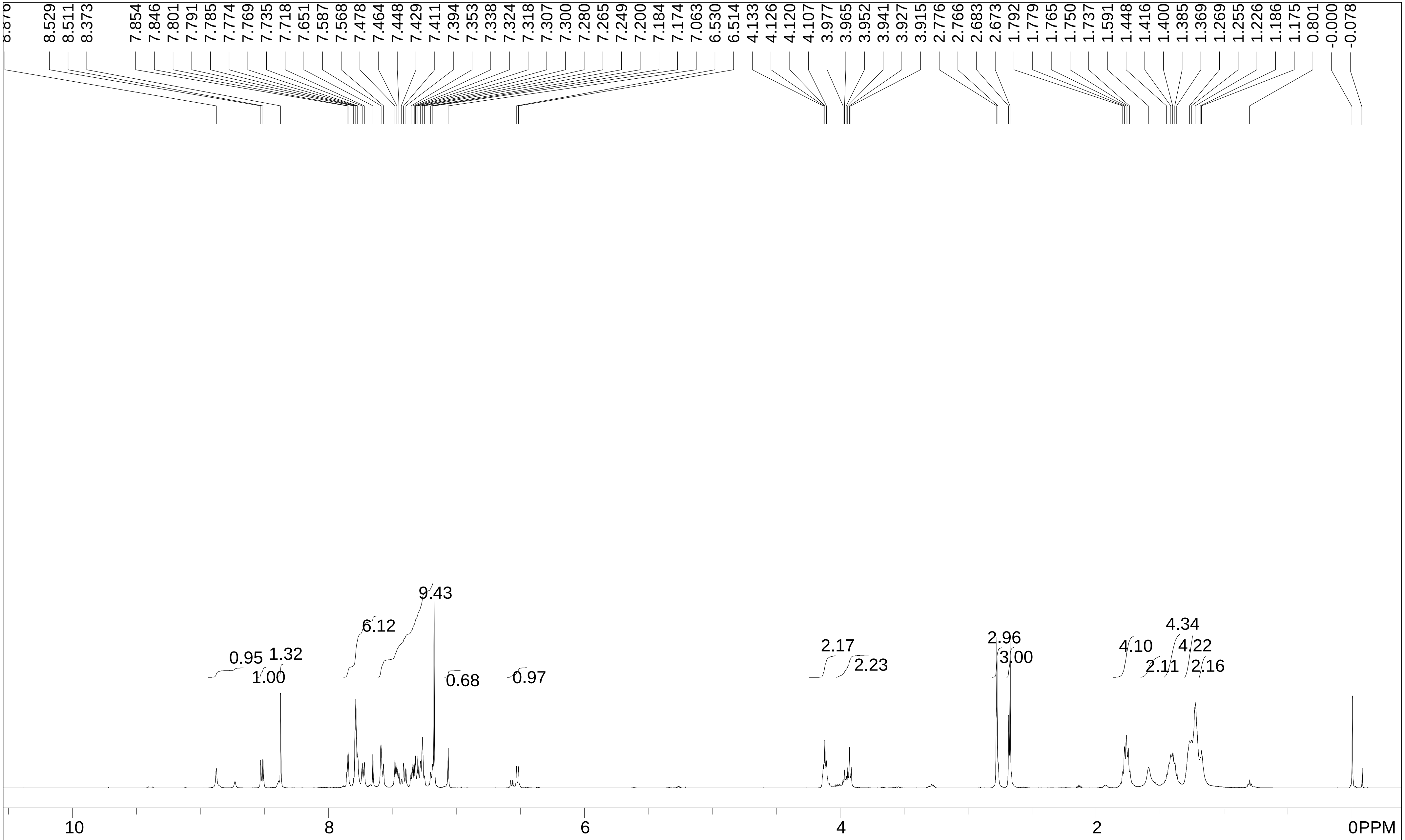

nmrB004, CDCl3,

USER: nmr -- DATE: Tue Apr 13 01:23:38 2021

F1: 500.133	F2: 1.000	SW1: 10331		OF1: 3032.9		PTS1d: 32768	
EX: zg30		PW: 13.7 usec	PD: 1.0 sec	NA: 8	LB: 0.0		

LDATTEC-C1b 1H NMR

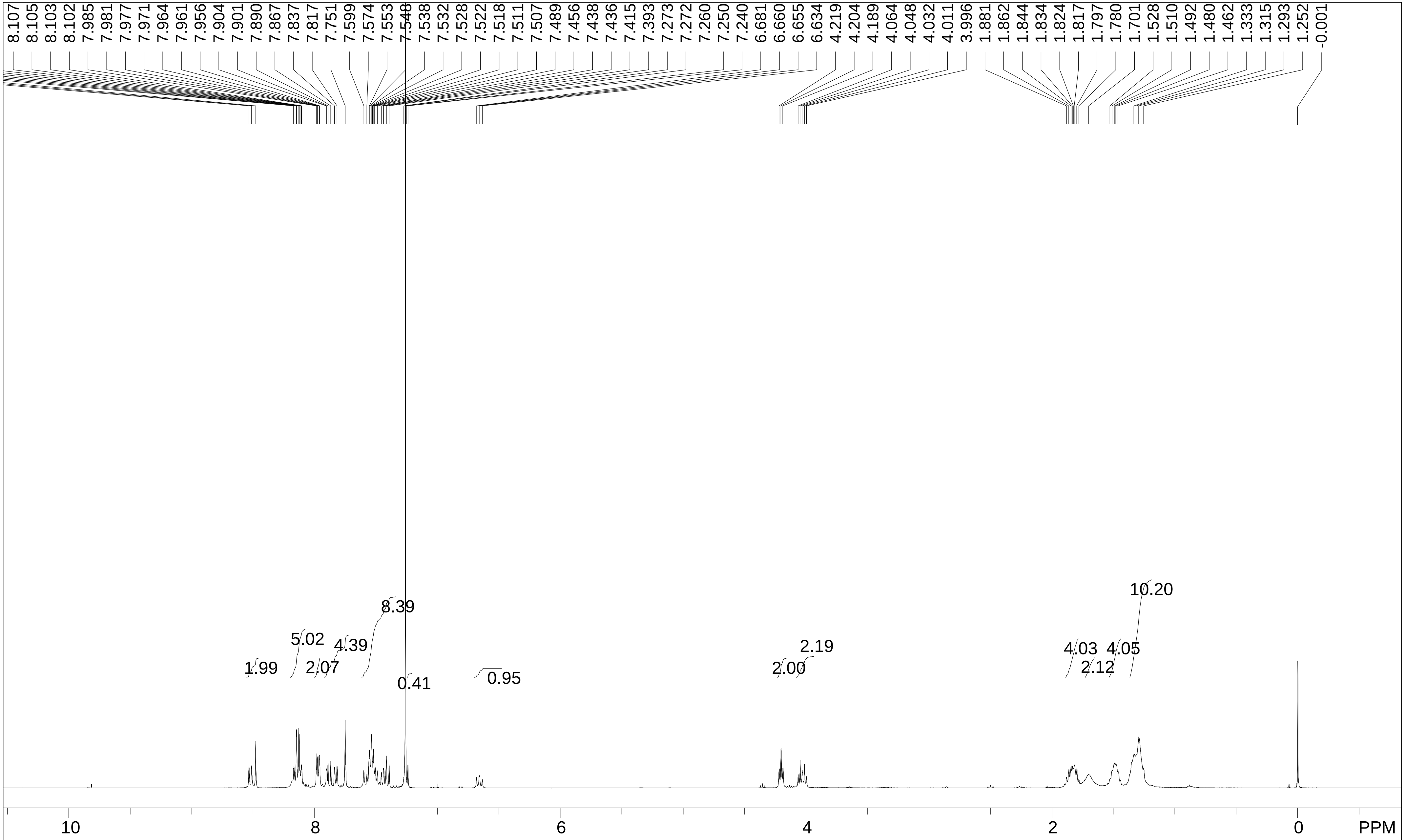

nmrB005, CDCl3,

USER: nmrb301 -- DATE: Wed Mar 31 01:09:58 2021

F1: 400.132	F2: 1.000	SW1: 8224		OF1: 2461.3		PTS1d: 32768	
EX: zg30		PW: 13.0 usec	PD: 1.0 sec	NA: 32	LB: 0.0		

LDATTEC-C3b 1H NMR
